# Dipeptide analogues of fluorinated aminophosphonic acid sodium salts as moderate competitive inhibitors of cathepsin C

**DOI:** 10.3762/bjoc.19.33

**Published:** 2023-04-12

**Authors:** Karolina Wątroba, Małgorzata Pawełczak, Marcin Kaźmierczak

**Affiliations:** 1 Faculty of Chemistry, Adam Mickiewicz University in Poznań, Uniwersytetu Poznańskiego 8, 61-614 Poznań, Poland; 2 Institute of Chemistry, Opole University, 45-052 Opole, Poland; 3 Centre for Advanced Technologies, Adam Mickiewicz University in Poznań, Uniwersytetu Poznańskiego 10, 61-614 Poznań, Poland

**Keywords:** aminophosphonates, cathepsin C, dipeptide, fluorine, solvolysis

## Abstract

In this paper, we present the solvolysis reaction of dipeptide analogues of fluorinated aminophosphonates with simultaneous quantitative deprotection of the amino group. To the best of our knowledge, this work is the first reported example of the application of fluorinated aminophosphonates in cathepsin C inhibition studies. The new molecules show moderate inhibition of the cathepsin C enzyme, which opens the door to consider them as potential therapeutic agents. Overall, our findings provide a new avenue for the development of fluorinated aminophosphonate-based inhibitors.

## Introduction

Cathepsin C, also known as dipeptidyl peptidase I (DPPI) belongs to the family of lysosomal cysteine proteases encompassing 11 human enzymes (cathepsins B, C, F, H, K, L, O, S, V, W, and X) [1]. Cathepsin C is considered a good target for designing new anticancer agents with broad substrate specificity [2].

Cathepsin C, which affects the processing of keratin, is of great importance in maintaining the structural organization of the epidermis, primarily the extremities, and the integrity of the teeth' tissues. Genetic studies have shown that a mutation in the gene encoding DPPI leads to early periodontitis, premature tooth loss, and keratosis of the palms and soles [3–4]. These conditions occur in Papillon–Lefevre syndrome and Haim–Munk syndrome [1]. Cathepsin C is an emerging pharmacological target due to its involvement in inflammatory and autoimmune diseases. Cathepsin C is upregulated in immune-related cells. DPPI also plays a role in the development of cancer – particularly in the liver and breast, hence the potential contribution of its inhibitors in chemotherapies to support traditional anticancer drugs. Moreover, there is a growing interest in the topic of cathepsin C inhibition, which directly affects serine protease activity [5–6]. Inhibitors of cathepsin C can be cystatins that show activity against a large group of cysteine proteases [7]. Other inhibitors are dipeptide derivatives showing substrate-like sequences. One of the most effective inhibitors is the dipeptide Gly-Phe-CHN_2_ (glycylphenylalanine-diazomethane), which, however, has not been used as a therapeutic substance due to the instability of the diazomethylketone group [8–9]. Based on its structure, many other inhibitors have been developed, such as vinyl sulfones, fluoromethyl ketones, and semicarbazides [8–9]. These inhibitors covalently bind to the nucleophilic thiol group of Cys234 in the active site of cathepsin C via a thioether bond.

Phosphonates have been identified as potential inhibitors of cathepsins. The phosphorus atom by default should mimic the tetrahedral intermediate, but this role may also be played by the hydroxy group present in hydroxyphosphonates, which mimic the carbonyl carbon in the peptide bond by forming a hydrogen bond with the amino group of the catalytic cysteine Cys234 [10]. Phosphonates, as well as their analogues phosphonic acids, can be modified in a number of ways, one of which is the introduction of a fluorine atom into their molecules by fluorination or alkylfluorination [11–14]. However, the reaction of β-aminoalcohols with nucleophilic deoxyfluorinating reagents often does not lead to the expected products with a fluorine atom in place of the –OH group. They usually undergo rearrangement, and intramolecular cyclization leading to products that are constitutional isomers [15].

The solvolysis reaction of phosphonates to the corresponding phosphonic acids or their salts is often a necessary step to measure activity in enzyme inhibition bioassays [16–22]. Therefore, our goal was to determine the best conditions for carrying out the solvolysis reaction of synthesized dipeptide analogues of fluorinated aminophosphonates [23–24] with the simultaneous deprotection of the amino group. The free amines were subjected to kinetic studies to investigate their interaction with cathepsin C. The required steps should be simple and fast, and the conditions of the reactions should be as mild as possible. The reactions should proceed with high yields, and any byproducts should be easily removeable.

## Results and Discussion

**Scheme 1:**
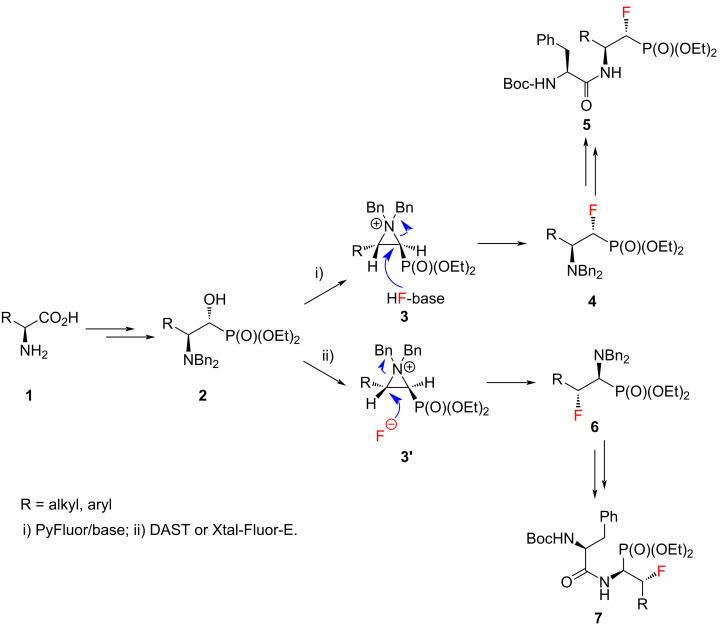
Synthetic strategy towards **5** and **7**.

Dipeptide analogues of α- and β-fluorinated aminophosphonates **5** and **7** were obtained from the corresponding ʟ-amino acids [23–24] **1**. In the key step of the synthesis fluorine was introduced to the corresponding α- and β-fluorinated aminophosphonates **4**, **6** (Scheme 1) by regioselective deoxyfluorination reactions of α-hydroxy-β-aminophosphonates **2** [24–26].

**Scheme 2:**
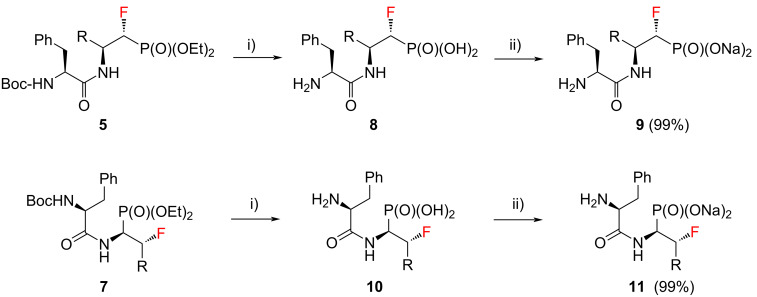
Synthesis of **9** and **11**. (**a**) R = -CH_3_; (**b**) R = -CH(CH_3_)_2_; (**c**) R = -CH_2_CH(CH_3_)_2_; (**d**) R = -CH(CH_3_)CH_2_CH_3_, (**e**) R = -CH_2_Ph; i) (a) **5** or **7** (1 equiv), TMSBr (8 equiv), CH_2_Cl_2_, rt, 20 h, (b) MeOH, 30 min.; ii) 8 or 10 (1 equiv), 1 M NaOH (2 equiv), H_2_O, 15 min.

Next, the conditions for the solvolysis were carefully assembled (Scheme 2). The optimized reaction conditions included 8 equiv of trimethylsilyl bromide (TMSBr) and freshly distilled methylene chloride as a solvent. In each case the reactions were carried out at room temperature overnight under an argon atmosphere. The next day, the solvent, volatile byproducts, and TMSBr residues were thoroughly evaporated. Time of solvolysis reactions varies in the literature, ranging from 10 minutes to several hours [27–29]. In our case the alcoholysis was carried out for 30 minutes. During this process, disappearance of the brownish or yellowish color of the compounds was observed. According to the literature, addition of diethyl ether in the next step should make precipitation more efficient [10]. This was done for compound **8a**, but no improvement was observed. Much better results were obtained with additional double wash of the precipitate with methanol combined with evaporation of the solvent under reduced pressure. As a result of the reactions carried out, the dipeptide analogues of α- and β-fluorinated aminophosphonic acids **8** and **10** were obtained. All the samples were solids, with very poor solubility in water and organic solvents such as DMSO and MeOH.

The final step of the synthesis was the reaction of the resulting phosphonic acids **8** and **10** with a 1 M aqueous NaOH solution. Based on the literature data, alternatively to this method [30–32], phosphonic acids can be passed through an ion exchange column [33]. The reactions of compounds **8** and **10** with 1 M NaOH were carried out at room temperature (Scheme 2). When a clear solutions were obtained, the reaction was carried out for another 15 minutes. The solutions were then concentrated under reduced pressure. The precipitated salts were washed with methanol [31] and the solvent evaporation procedure was repeated. Sublimation drying (lyophilization) was carried out, obtaining white powders with a yield of 99% in each case. The resulting sodium salts of phosphonic acids **9** and **11** were subjected to ^1^H, ^13^C, ^19^F, and ^31^P NMR spectroscopic analysis as well as mass spectrometry (MS) confirming their purity. The spectroscopic data of **9** and **11** are in agreement with the literature data of the starting esters **5** and **7** literature data [23–24]. A very good correlation of chemical shifts was also observed in the ^13^C NMR spectra for the key signals from the C1 and C2 atoms (Table 1). Each sample was pure; no byproducts were present.

**Table 1:** The ^13^C NMR chemical shifts of C1 and C2 carbon atoms.

	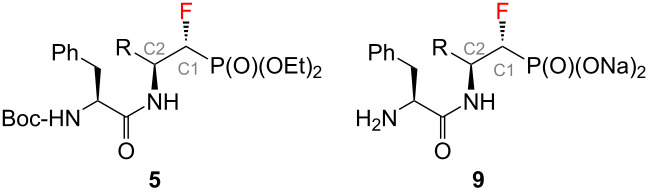			

R =	**5** C1 [ppm]	**9** C1 [ppm]	**5** C2 [ppm]	**9** C2 [ppm]

(**a**) -CH_3_	89.56	94.72	45.66	47.77
(**b**) -CH(CH_3_)_2_	88.97	93.30	54.77	57.29
(**c**) -CH_2_CH(CH_3_)_2_	91.33	95.36	48.62	50.22
(**d**) -CH(CH_3_)CH_2_CH_3_	88.89	92.17	54.45	56.33
(**e**) -CH_2_Ph	88.05	94.79	51.39	52.93

	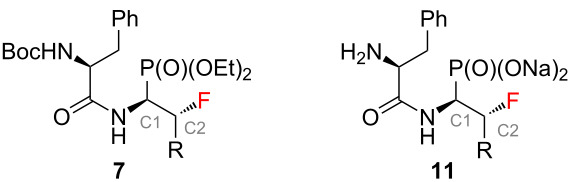			

R =	**7** C1 [ppm]	**11** C1 [ppm]	**7** C2 [ppm]	**11** C2 [ppm]

(**a**) -CH_3_	49.51	54.13	89.00	92.58
(**b**) -CH(CH_3_)_2_	47.55	50.45	98.03	99.23
(**c**) -CH_2_CH(CH_3_)_2_	50.00	54.09	92.21	94.76
(**d**) -CH(CH_3_)CH_2_CH_3_	47.16	49.91	95.16	96.08
(**e**) -CH_2_Ph	48.69	53.55	93.24	96.21

### Kinetic studies

Due to the homology and similar structural requirements, bovine cathepsin C is often used in research as a model for human cathepsin C as it was well documented by Poręba et al. [34] in the study of the substrate specificity of these two mammalian cathepsins. They showed the best fit of amino acids with larger side to the S1 pocket of the enzyme. In contrast, the S2 pocket preferably accommodates amino acids having short aliphatic side-chains, but also recognizes aromatic amino acids, preferably phenylalanine. To study the structural requirements of the S1 binding site of the enzyme, we synthesized a series of ten dipeptide analogues of fluorinated aminophosphonic acid sodium salts **9**, **11** with phenylalanine at the *N*-terminus and evaluated their inhibitory activity against bovine cathepsin C. Inhibition kinetics were carried out at 37 °C for 10 minutes in acetate buffer at pH 5. Changes in product concentration versus time were monitored spectrophotometrically at λ = 405 nm. Seven of the tested compounds were moderate competitive inhibitors with millimolar inhibitory activity (Table 2). Three of them at higher concentrations precipitated from 0.1 M acetate buffer at pH 5.0. Dipeptide analogues of α-fluorinated aminophosphonic acid sodium salts **9** were more active against cathepsin C than β-fluorinated analogues **11**.

**Table 2:** Inhibitory constants of the studied of α- and β-fluorinated aminophosphonic acid sodium salts towards bovine cathepsin C.

Dipeptide	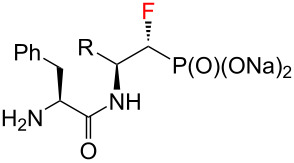 **9** *K*_I_ ± SD [mM]	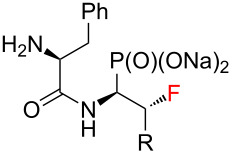 **11** *K*_I_ ± SD [mM]

Phe-Ala (**a**) -CH_3_	0.603 ± 0.1	0.733 ± 0.087
Phe-Val (**b**) -CH(CH_3_)_2_	0.0951 ± 0.05	1.869 ± 0.171
Phe-Leu (**c**) -CH_2_CH(CH_3_)_2_	0.309 ± 0.066	0.847 ± 0.38
Phe-Ile (**d**) -CH(CH_3_)CH_2_CH_3_	0.273 ± 0.15	up to a concentration of 0.37 mM, it does not inhibit activity; at a higher concentration, it precipitates
Phe-Phe (**e**) -CH_2_Ph	precipitates in a buffer	precipitates in buffer

**Figure 1:**
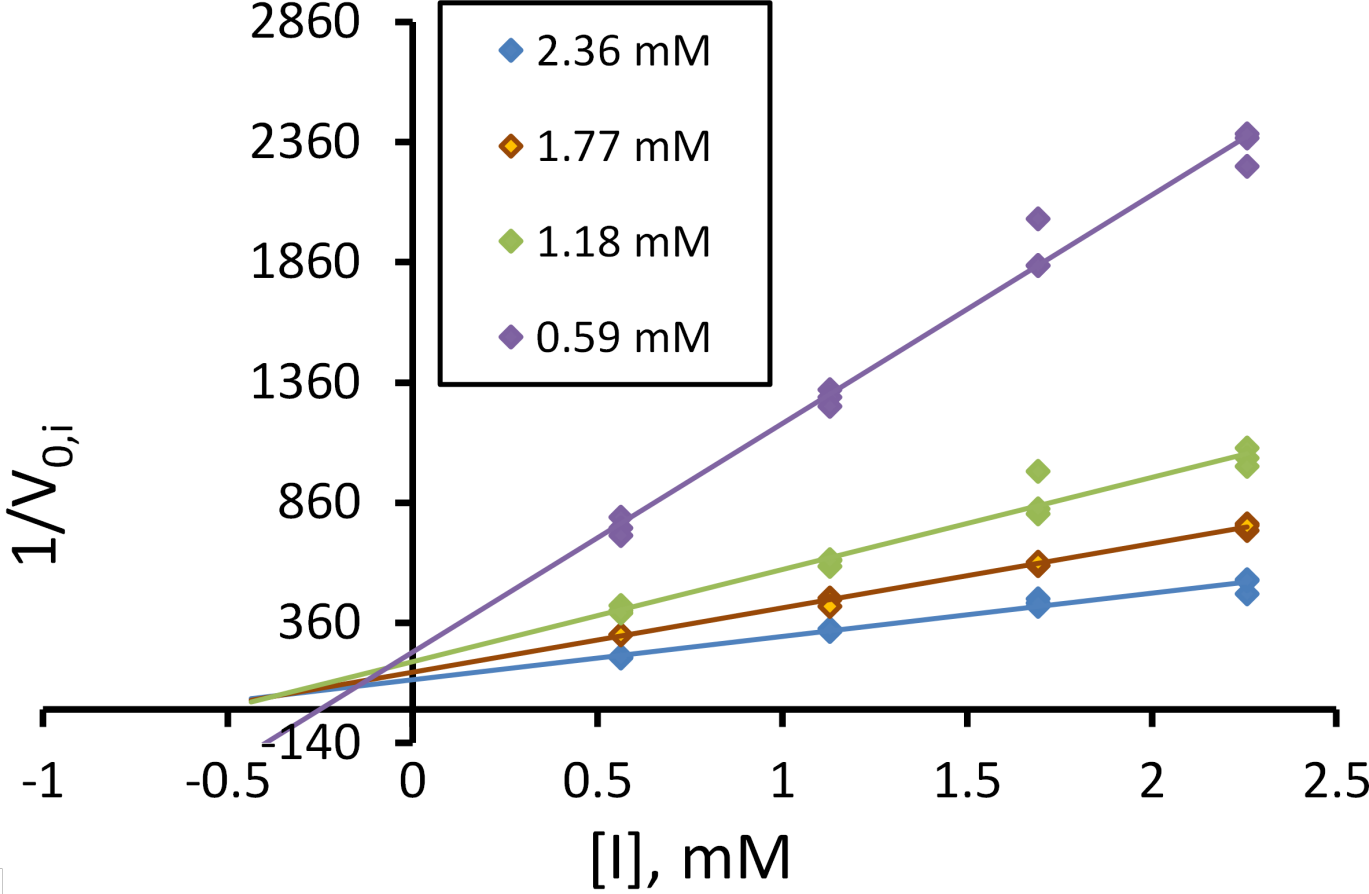
Dixon plot for the hydrolysis of Gly-Phe-*p*NA substrate catalyzed by bovine cathepsin C in the presence of **9b** (*T* = 37 °C, pH 5.0).


The dipeptide analogue of α-fluorinated aminophosphonic acid sodium salt bearing the valine residue as a second amino acid in the chain (**9b**) showed the greatest inhibitory power (Figure 1). The type of inhibition and the inhibition constant were determined from the Dixon-type linearization of Equation 1 [35]. For each of the simple data, Equation 1 determines the slope factor *a*, whereby *a* weighted fit was used. The statistical weight for each point in the above-mentioned transformation 1/*V*_0,i_ = *f*(*I*) is equal to 
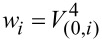
.

[1] $$V_{\text{0,i}}=\frac{V_{\max}\cdot [\text{S}]}{K_{\text{M}}\cdot \left(1+\frac{\left[\text{I}\right]}{K_{\text{ic}}}\right)+[\text{S}]\cdot \left(1+\frac{\left[\text{I}\right]}{K_{\text{iu}}}\right)}$$

where:

*V*_max_ = maximum reaction velocity, *K*_M_ = Michaelis constant, *K*_ic_ = competitive inhibitory constant, *K*_iu_ = uncompetitive inhibitory constant, [S] = concentration of the substrate, [I] = concentration of the inhibitor.

## Conclusion

In conclusion, we demonstrated the solvolysis reaction of dipeptide analogues of fluorinated aminophosphonates with the simultaneous deprotection of the amino group. The resulting acids were converted into the corresponding salts. All the reactions proceeded quantitatively. Obtained compounds were subjected to kinetic studies against cathepsin C, and the results indicated that they are moderate competitive inhibitors of this enzyme. This study presented the first kinetic investigation of fluorinated dipeptide derivatives of aminophosphonic acid salts against cathepsin C, thus contributing to the development of the novel cathepsin C inhibitors. We are currently working on the development of more effective fluorinated inhibitors of cathepsin C in our laboratory.

## Supporting Information

File 1 Experimental procedures, biological protocol, NMR data.
