# Supplementary material for: Dipeptide analogues of fluorinated aminophosphonic acid sodium salts as moderate competitive inhibitors of cathepsin C
Source: Beilstein J Org Chem. 2023 Apr 12;19:434–9. doi: 10.3762/bjoc.19.33 (PMC10113521; doi:10.3762/bjoc.19.33)
Supplement: File 1 — Experimental procedures, biological protocol, NMR data. [file Beilstein_J_Org_Chem-19-434-s001.pdf]

BEILSTEIN JOURNAL OF ORGANIC CHEMISTRY

Supporting Information

for

Dipeptide analogues of fluorinated aminophosphonic acid sodium salts as moderate competitive inhibitors of cathepsin C

Karolina Wątroba, Małgorzata Pawełczak and Marcin Kaźmierczak

Beilstein J. Org. Chem. **2023,** *19,* 434–439. doi:10.3762/bjoc.19.33

Experimental procedures, biological protocol, NMR data

License and Terms: This is a supporting information file under the terms of the Creative Commons Attribution License (https://creativecommons.org/licenses/by/4.0). Please note that the reuse, redistribution and reproduction in particular requires that the author(s) and source are credited and that individual graphics may be subject to special legal provisions.
The license is subject to the *Beilstein Journal of Organic Chemistry* terms and conditions: (https://www.beilstein-journals.org/bjoc/terms)

1.General information……………………………………………………………..……………..S2

2.General synthetic procedure and analytical data of **9** and **11**..……………….…………….....S2

3.Enzyme kinetics study…………………………………….……………………………….……S7

4. NMR data……………………...…………………………………………………………..…..S8

1. General information

^1H NMR, ^{13}C NMR, ^{19}F NMR and ^{31}P NMR spectra were performed on Bruker ASCEND 400 (400 MHz), Bruker ASCEND 600 (600 MHz) spectrometers. Chemical shifts of ^1H, ^{13}C NMR were expressed in parts per million downfield from 1,4-dioxane as an internal standard in D_2O. Chemical shifts of ^{19}F, ^{31}P NMR were expressed in parts per million. High-resolution mass spectra were recorded by electron spray (MS-ESI) techniques using QToF Impact HD Bruker spectrometer. CH_2Cl_2 was dried by distillation with CaH_2 under argon atmosphere. All moisture-sensitive reactions were carried out under argon atmosphere using oven-dried glassware. TLC was performed on Merck Kieselgel 60-F254 with EtOAc/hexane and MeOH/$CHCl_3$ as developing systems, and products were detected by inspection under UV light (254 nm) and with a solution of potassium permanganate.

Kinetic assays: Cathepsin C from bovine spleen was purchased from Sigma Aldrich. Gly-*L*-Phe-pNA·TFA was synthesized by Dr. Maciej Makowski. The remaining reagents were purchased pure for analytical testing.

2. General synthetic procedure and analytical data of 9 and 11:

To a solution of *N*-protected dipeptide analogs of fluorinated aminophosphonates **5** or **7** (1 equiv) in freshly distilled CH_2Cl_2 (1.2 mL), TMSBr (8 equiv) was added at room temperature under argon atmosphere. It was left stirring overnight (about 20 hours). After this time, the reaction mixtures were concentrated to dryness under reduced pressure. Methanol (1.5 mL) was then added and stirred for 30 minutes. The mixtures were concentrated under reduced pressure. The crude products were washed with methanol two more times, each time concentrated to dryness under reduced pressure. Phosphonic acids **8** and **10** were obtained.

H_2O (1.5 mL) and 1 M NaOH solution (2 equiv) were added to phosphonic acids **8** or **10**. The mixtures were stirred for 15 minutes after the solids were completely dissolved. Solutions were evaporated under reduced pressure. The crude products were washed with methanol two more times, each time concentrated to dryness under reduced pressure. The obtained salts **9** and **11** were lyophilized obtaining white solids in each case.

Sodium ((1*R*,2*S*)-2-((*S*)-2-amino-3-phenylpropanamido)-1-fluoropropyl)phosphonate (**9a**, white solid, yield 99%). **^{1}H NMR** (600 MHz, D_2O) δ = 7.58 – 7.05 (m, 5H, Ar*H*), 4.36 (ddd, *J* = 46.2, 6.7, 2.2 Hz, 1H, C*H*FP), 4.30 – 4.18 (m, 1H NC*H*CH_3), 3.63 (t, *J* = 6.4 Hz, 1H, PhCH_2C*H*), 3.00 – 2.84 (m, 2H, PhC*H_2*), 1.22 (d, *J* = 7.0 Hz, 3H, C*H_3*). **^{13}C NMR** (151 MHz, D_2O) δ = 175.86 (s, *C*=O), 137.63, 130.05, 129.33, 127.60 (4 x s, Ar), 94.72 (dd, *J* = 178.2, 146.9 Hz, *C*HFP), 56.82 (s, PhCH_2*C*HCO), 47.77 (dd, *J* = 19.6, 5.8 Hz, CH_3*C*HNH), 40.92 (s, Ph*C*H_2CHCO), 14.98 (d, *J* = 6.1 Hz, *C*H_3CHNH). **^{19}F NMR** (565 MHz, D_2O) δ = -213.76 (ddd, *J* = 61.9, 46.3, 30.0 Hz). **^{19}F {/^{1}H} NMR** (565 MHz, D_2O) δ = -213.69 (d, *J* = 63.5 Hz). **^{31}P {/^{1}H} NMR** (243 MHz, D_2O) δ = 9.10 (d, *J* = 61.9 Hz). **HRMS (ESI)** calcd. for $C_{12}H_{16}FN_2O_4P$ ($[M+3H]^+$): 305.1066, found: 305,1075.

Sodium ((1*R*,2*S*)-2-((*S*)-2-amino-3-phenylpropanamido)-1-fluoro-3-methylbutyl)-phosphonate (**9b**, white solid, yield 99%). **^{1}H NMR** (600 MHz, D_2O) δ = 7.51 – 7.11 (m, 5H, Ar*H*), 4.71 – 4.51 (m, 1H, C*H*FP), 4.15 – 3.91 (m, 1H, $(CH_3)_2$CHN*H*), 3.74 – 3.70 (m, 1H, PhCH_2C*H*CO), 3.22 – 2.73 (m, 2H, PhC*H_2*CHCO), 2.34 – 2.06 (m, 1H, $(CH_3)_2$C*H*), 1.03 – 0.72 (m, 6H, (C*H_3*)(C*H_3*)CH). **^{13}C NMR** (151 MHz, D_2O) δ = 177.18 (s, *C*=O), 138.01, 130.15, 129.35, 127.54 (4 x s, Ar), 93.30 (dd, *J* = 176.6, 146.7 Hz, *C*HFP), 57.29 (m, PhCH_2*C*HCO, $(CH_3)_2$CH*C*HN), 40.75 (s, Ph*C*H_2CHCO), 29.32 (d, *J* = 4.8 Hz, $(CH_3)_2$*C*H), 20.83 (s, *C*H_3), 18.88 (s, *C*H_3). **^{19}F NMR** (565 MHz, D_2O) δ = -203.82 (ddd, *J* = 65.8, 44.8, 22.9 Hz). **^{19}F NMR {/^{1}H}** (565 MHz, D_2O) δ = -203.78 (d, *J* = 63.1 Hz). **^{31}P NMR {/^{1}H}** (243 MHz, D_2O) δ = 9.65 (d, *J* = 62.9 Hz). **HRMS (ESI)** calcd. for $C_{14}H_{20}FN_2O_4P$ ($[M+3H]^+$): 333,1379, found: 333,1374.

Sodium ((1*R*,2*S*)-2-((*S*)-2-amino-3-phenylpropanamido)-1-fluoro-4-methylpentyl)phosphonate (**9c**, white solid, yield 99%). **^{1}H NMR** (600 MHz, D_2O) δ = 7.52 – 7.16 (m, 5H, Ar*H*), 4.40 (ddd, *J* = 45.7, 6.6, 2.7 Hz, 1H, C*H*FP), 4.35 – 4.23 (m, 1H, $(CH_3)_2$CHCH_2C*H*NH), 3.66 (t, *J* = 6.7 Hz, 1H, PhCH_2C*H*CO), 2.96 (qd, *J* = 13.7, 6.7 Hz, 2H, PhC*H_2*CHCO), 1.64 (ddd, *J* = 13.5, 10.2, 2.5 Hz, 1H, $(CH_3)_2$CHC*H*H), 1.52 (ddd, *J* = 14.5, 11.5, 4.2 Hz, 1H, $(CH_3)_2$CHCH*H*), 1.40 (dqt, *J* = 12.5, 9.6, 4.9 Hz, 1H, $(CH_3)_2$C*H*), 0.90 – 0.85 (m, 6H, (C*H_3*)(C*H_3*)CH). **^{13}C NMR** (151 MHz, D_2O) δ = 176.47 (s, *C*=O), 137.70, 130.07, 129.35, 127.57 (4 x s, Ar), 95.36 (dd, *J* = 178.0, 146.6 Hz, *C*HFP), 56.96 (s, PhCH_2*C*HCO), 50.22 (dd, *J* = 19.1, 5.0 Hz, $(CH_3)_2$CHCH_2*C*H), 40.76 (s, Ph*C*H_2CHCO), 37.46 (d, *J* = 5.1 Hz $(CH_3)_2$CH*C*H_2CH), 24.78 (s, $(CH_3)_2$*C*H), 23.52

(s, CH_3), 21.35 (s, CH_3). **^{19}F NMR** (376 MHz, D_2O) δ = -210.77 (ddd, *J* = 62.9, 45.8, 28.3 Hz). **^{19}F NMR {/^{1}H}** (565 MHz, D_2O) δ = -210.77 (d, *J* = 62.5 Hz). **^{31}P NMR {/^{1}H}** (243 MHz, D_2O) δ = 9.17 (d, *J* = 62.6 Hz). **HRMS (ESI)** calcd. for $C_{15}H_{22}FN_2O_4P$ ($[M+3H]^+$): 347,1536, found: 347,1539.

Sodium ((1*R*,2*S*,3*S*)-2-((*S*)-2-amino-3-phenylpropanamido)-1-fluoro-3-methyl-pentyl)phosphonate (**9d**, white solid, yield 99%). **^{1}H NMR** (600 MHz, D_2O) δ = 7.78 – 7.32 (m, 5H, Ar*H*), 4.95 – 4.77 (m, 1H, C*H*FP), 4.38 – 4.21 (m, 1H, $CH_3CH_2CH(CH_3)C\mathit{H}NH$), 3.89 (dd, *J* = 7.7, 5.1 Hz, 1H, $PhCH_2C\mathit{H}CO$), 3.26 (dd, *J* = 13.8, 5.3 Hz, 1H, PhC*H*HCHCO), 3.07 (dd, *J* = 14.1, 8.0 Hz, 1H, PhCH*H*CHCO), 2.13 – 2.03 (m, 1H, $CH_3CH_2C\mathit{H}(CH_3)CHNH$), 1.72 – 1.56 (m, 1H, $CH_3C\mathit{H}HCH(CH_3)CHNH$), 1.14 – 1.07 (m, 4H, $CH_3CH_2CH(C\mathit{H_3})CHNH$, $CH_3CH\mathit{H}CH(CH_3)CHNH$), 1.03 – 0.96 (m, 3H, $C\mathit{H_3}CH_2CH(CH_3)CHNH$). **^{13}C NMR** (151 MHz, D_2O) δ = 176.60 (s, *C*=O), 137.34 (s, Ar), 129.60 (s, Ar), 128.74 (d, *J* = 2.3 Hz, Ar), 126.94 (s, Ar), 92.17 (dd, *J* = 175.7, 147.0 Hz, *C*HFP), 56.53 (s, $PhCH_2\mathit{C}HCO$), 56.33 (d, *J* = 18.2 Hz, $CH_3CH_2CH(CH_3)\mathit{C}HNH$), 39.87 (s, $Ph\mathit{C}H_2$), 34.94 (d, *J* = 5.0 Hz, $CH_3CH_2\mathit{C}H(CH_3)CHNH$), 24.74 (d, *J* = 2.1 Hz, $CH_3\mathit{C}H_2$), 15.98 (s, $\mathit{C}H_3CH$), 10.41 (s, $\mathit{C}H_3CH_2$). **^{19}F NMR** (565 MHz, D_2O) δ = -201.20 (ddd, *J* = 64.0, 44.2, 20.4 Hz). **^{19}F NMR {/^{1}H}** (565 MHz, D_2O) δ = -201.16 (d, *J* = 63.3 Hz). **^{31}P NMR {/^{1}H}** (243 MHz, D_2O) δ = 9.80 (d, *J* = 63.4 Hz). **HRMS (ESI)** calcd. for $C_{15}H_{22}FN_2O_4P$ ($[M+3H]^+$): 347,1536, found: 347,1535.

Sodium ((1*R*,2*S*)-2-((*S*)-2-amino-3-phenylpropanamido)-1-fluoro-3-phenyl-propyl)phosphonate (**9e**, white solid, yield 99%). **^{1}H NMR** (400 MHz, D_2O) δ = 7.51 – 6.98 (m, 10H, Ar*H*), 4.66 – 4.36 (m, 2H, C*H*FP, $PhCH_2C\mathit{H}NH$), 3.48 – 3.38 (m, 2H, PhC*H*HCHNH, $PhCH_2C\mathit{H}CO$), 2.87 – 2.69 (m, 2H, PhCH*H*CHNH, PhC*H*HCHCO), 2.60 (dd, *J* = 13.5, 7.5 Hz, 1H, PhCH*H*CHCO). **^{13}C NMR** (101 MHz, D_2O) δ = 176.36 (s, *C*=O), 139.57, 137.98, 130.16, 129.86, 129.31, 129.05, 127.49, 127.06 (8 x s, Ar), 94.79 (dd, *J* = 178.8, 146.2 Hz, *C*HFP), 57.10 (s, $PhCH_2\mathit{C}HCO$), 52.93 (dd, *J* = 19.0, 5.6 Hz, $PhCH_2\mathit{C}HNH$), 40.75 (s, $Ph\mathit{C}H_2CHCO$), 35.17 (d, *J* = 5.7 Hz, $Ph\mathit{C}H_2CHNH$). **^{19}F NMR** (376 MHz, D_2O) δ = -212.26 (ddd, *J* = 62.0, 45.8, 29.1 Hz). **^{19}F NMR {/^{1}H}** (376 MHz, D_2O) δ = -212.23 (d, *J* = 61.7 Hz). **^{31}P NMR {/^{1}H}** (162 MHz, D_2O) δ = 8.85 (d, *J* = 61.7 Hz). **HRMS (ESI)** calcd. for $C_{18}H_{20}FN_2O_4P$ ($[M+3H]^+$): 381,1379, found: 381,1379.

Sodium ((1*S*,2*R*)-1-((*S*)-2-amino-3-phenylpropanamido)-2-fluoropropyl)phosphonate (**11a**, white solid, yield 99%). **^{1}H NMR** (600 MHz, D_2O) δ = 7.51 – 7.14 (m, 5H, Ar*H*), 5.00 (dqd, *J* = 46.6, 6.4, 3.2 Hz, 1H, C*H*F), 4.27 (td, *J* = 19.1, 3.0 Hz, 1H, C*H*P), 3.89 (t, *J* = 7.5 Hz, 1H, $PhCH_2C$*H*CO), 3.14 – 2.97 (m, 2H, PhCH_2CHCO), 1.00 (dd, *J* = 26.0, 6.4 Hz, 3H, CH_3). **^{13}C NMR** (151 MHz, D_2O) δ = 174.58 (d, *J* = 5.6 Hz, *C*=O), 136.75, 130.04, 129.50, 127.78 (4 x s, Ar), 92.58 (dd, *J* = 168.4, 11.9 Hz, *C*HF), 56.96 (s, $PhCH_2$*C*HCO), 54.13 (dd, *J* = 129.9, 15.9 Hz, *C*HP), 39.91 (s, Ph*C*H_2CHCO), 16.03 (d, *J* = 22.8 Hz, *C*H_3). **^{19}F NMR** (565 MHz, D_2O) δ = -166.26 – -167.15 (m). **^{19}F NMR {/^{1}H}** (565 MHz, D_2O) δ = -166.65 (d, *J* = 44.9 Hz). **^{31}P NMR {/^{1}H}** (243 MHz, D_2O) δ = 10.48 (d, *J* = 44.4 Hz). **HRMS (ESI)** calcd. for $C_{12}H_{16}FN_2O_4P$ ($[M+3H]^+$): 305,1066, found: 305,1068.

Sodium ((1*S*,2*R*)-1-((*S*)-2-amino-3-phenylpropanamido)-2-fluoro-3-methylbutyl)-phosphonate (**11b**, white solid, yield 99%). **^{1}H NMR** (600 MHz, D_2O) δ = 7.66 – 7.12 (m, 5H, Ar*H*), 4.13 – 3.93 (m, 2H, C*H*F, C*H*P), 3.66 (dd, *J* = 9.2, 6.2 Hz, 1H, $PhCH_2C$*H*CO), 2.94 (dd, *J* = 13.4, 6.2 Hz, 1H, PhC*H*HCHCO), 2.84 (dd, *J* = 13.4, 9.3 Hz, 1H, PhCH*H*CHCO), 1.34 – 1.14 (m, 1H, $(CH_3)(CH_3)C$*H*), 0.80 (d, *J* = 6.9 Hz, 3H, (CH_3)(CH_3)CH), 0.71 (d, *J* = 6.7 Hz, 3H, (CH_3)(CH_3)CH). **^{13}C NMR** (151 MHz, D_2O) δ = 176.00 (d, *J* = 5.0 Hz, *C*=O), 137.85, 129.87, 129.32, 127.56 (4 x s, Ar), 99.23 (d, *J* = 175.4 Hz, *C*HF), 57.48 (s, $PhCH_2$*C*HCO), 50.45 (dd, *J* = 134.4, 27.3 Hz, *C*HP), 40.94 (s, Ph*C*H_2CHCO), 28.88 (dd, *J* = 19.9, 5.1 Hz, $(CH_3)_2$*C*H), 19.87 (d, *J* = 4.7 Hz, (*C*H_3)(CH_3)CH), 15.87 (d, *J* = 5.4 Hz, (CH_3)(*C*H_3)CH). **^{19}F NMR** (565 MHz, D_2O) δ = -194.75 – -196.20 (m). **^{19}F NMR {/^{1}H}** (565 MHz, D_2O) δ = -195.56 (br.s). **^{31}P NMR {/^{1}H}** (243 MHz, D_2O) δ = 12.40 (d, *J* = 8.7 Hz). **HRMS (ESI)** calcd. for $C_{14}H_{20}FN_2O_4P$ ($[M+3H]^+$): 333,1379, found: 333,1386.

Sodium ((1*S*,2*R*)-1-((*S*)-2-amino-3-phenylpropanamido)-2-fluoro-4-methyl-pentyl)phosphonate (**11c**, white solid, yield 99%). **^{1}H NMR** (400 MHz, D_2O) δ = 7.57 – 6.91 (m, 5H, Ar*H*), 5.04 – 4.81 (m, 1H, C*H*F), 4.21 (td, *J* = 19.7, 2.9 Hz, 1H, C*H*P), 3.79 – 3.70 (m, 1H, $PhCH_2C$*H*CO), 2.99 (dd, *J* = 13.3, 5.8 Hz, 1H, PhC*H*HCHCO), 2.94 – 2.82 (m, 1H, PhCH*H*CHCO), 1.69 – 1.47 (m, 1H, $(CH_3)_2C$*H*), 1.39 – 1.11 (m, 1H, $(CH_3)_2CHC$*H*H), 1.01 – 0.87 (m, 1H, $(CH_3)_2CHCH$*H*), 0.86 (d, *J* = 2.0 Hz, 3H, (CH_3)(CH_3)CH), 0.84 (d, *J* = 2.1 Hz, 3H, (CH_3)(CH_3)CH). **^{13}C NMR** (101 MHz, D_2O) δ = 175.42 (s, *C*=O), 137.14, 129.95, 129.42, 127.78 (4 x s, Ar), 94.76 (dd, *J* = 171.3,

11.7 Hz, *C*HF), 57.41 (s, PhCH2*C*HCO), 54.09 (dd, *J* = 130.3, 16.7 Hz, *C*HP), 40.36 (s, Ph*C*H2CHCO), 38.35 (d, *J* = 20.9 Hz, (CH3)(CH3)CH*C*H2), 24.67 (d, *J* = 1.7 Hz, (CH3)(CH3)*C*H), 23.34 (s, (*C*H3)(CH3)CH), 21.26 (s, (CH3)(*C*H3)CH). **^{19}F NMR** (376 MHz, D_2O) δ = -176.51 (tddd, *J* = 48.1, 44.1, 20.2, 14.4 Hz). **^{19}F NMR {/^{1}H}** (376 MHz, D_2O) δ = -176.50 (d, *J* = 44.0 Hz). **^{31}P NMR {/^{1}H}** (162 MHz, D_2O) δ = 10.44 (d, *J* = 44.0 Hz). **HRMS (ESI)** calcd. for $C_{15}H_{22}FN_2O_4P$ ($[M+3H]^+$): 347,1536, found: 347,1539.

Sodium ((1*S*,2*R*,3*S*)-1-((*S*)-2-amino-3-phenylpropanamido)-2-fluoro-3-methyl-pentyl)phosphonate (**11d**, white solid, yield 99%). **^{1}H NMR** (400 MHz, D_2O) δ = 7.45 – 7.12 (m, 5H, Ar*H*), 4.33 (dtd, *J* = 46.6, 8.4, 2.7 Hz, 1H, C*H*F), 4.03 (ddd, *J* = 14.2, 11.7, 8.3 Hz, 1H, C*H*P), 3.68 (dd, *J* = 9.3, 6.1 Hz, 1H, PhCH2C*H*CO), 3.01 – 2.80 (m, 2H, PhC*H2*CHCO), 1.27 (dq, *J* = 14.4, 7.2 Hz, 1H, CH3C*H*HCH(CH3)), 1.12 (dp, *J* = 14.4, 7.4 Hz, 1H, CH3CH*H*CH(CH3)), 0.96 – 0.81 (m, 1H, CH3CH2C*H*(CH3)), 0.77 (t, *J* = 7.4 Hz, 3H, C*H3*CH2CH(CH3)), 0.68 (d, *J* = 6.7 Hz, 3H, CH3CH2CH(C*H3*)). **^{13}C NMR** (101 MHz, D_2O) δ = 175.10 (d, *J* = 4.4 Hz, *C*=O), 137.56, 129.81, 129.39, 127.70 (4 x s, Ar), 96.08 (d, *J* = 174.2 Hz, *C*HF), 57.34 (s, PhCH2*C*HCO), 49.91 (dd, *J* = 135.0, 28.7 Hz, *C*HP), 40.58 (s, Ph*C*H2CHCO), 35.08 (dd, *J* = 19.8, 5.9 Hz, CH3CH2*C*H(CH3)), 26.55 (d, *J* = 3.3 Hz, CH3*C*H2CH(CH3)), 12.92 (d, *J* = 5.8 Hz, CH3CH2CH(*C*H3), 11.32 (s, *C*H3CH2CH(CH3)). **^{19}F NMR** (376 MHz, D_2O) δ = -197.79 – -198.71 (m). **^{19}F NMR {/^{1}H}** (376 MHz, D_2O) δ = -198.24 (br.s). **^{31}P NMR {/^{1}H}** (162 MHz, D_2O) δ = 12.95 (d, *J* = 5.3 Hz). **HRMS (ESI)** calcd. for $C_{15}H_{22}FN_2O_4P$ ($[M+3H]^+$): 347,1536, found: 347,1540.

Sodium ((1*S*,2*R*)-1-((*S*)-2-amino-3-phenylpropanamido)-2-fluoro-3-phenyl-propyl)phosphonate (**11e**, white solid, yield 99%). **^{1}H NMR** (600 MHz, D_2O) δ = 7.61 – 6.92 (m, 10H, Ar*H*), 5.10 – 4.87 (m, 1H C*H*F), 4.29 (td, *J* = 19.0, 3.2 Hz, 1H, C*H*P), 3.80 – 3.71 (m, 1H, PhCH2C*H*CO), 3.00 (dd, *J* = 13.3, 5.8 Hz, 1H, PhC*H*HCHF), 2.96 – 2.82 (m, 2H, PhC*H2*CHCO), 2.23 (td, *J* = 15.2, 10.9 Hz, 1H, PhCH*H*CHF). **^{13}C NMR** (151 MHz, D_2O) δ = 177.10 (d, *J* = 5.5 Hz, *C*=O), 138.96, 137.87, 130.02, 130.02, 129.38, 129.16, 127.58, 127.22 (8 x s, Ar), 96.21 (dd, *J* = 175.1, 10.8 Hz, *C*HF), 57.65 (s, PhCH2*C*HCO), 53.55 (dd, *J* = 130.0, 17.1 Hz, *C*HP), 41.16 (s, Ph*C*H2CHCO), 36.61 (d, *J* = 21.4 Hz, Ph*C*H2CHF). **^{19}F NMR** (565 MHz, D_2O) δ = -176.97 – -177.89 (m). **^{19}F NMR {/^{1}H}** (565 MHz, D_2O) δ = -177.43 (d, *J* = 40.8 Hz). **^{31}P NMR {/^{1}H}** (243 MHz,

D_2O) δ = 10.44 (d, *J* = 40.1 Hz). **HRMS (ESI)** calcd. for $C_{18}H_{20}FN_2O_4P$ ($[M+3H]^+$): 381,1379, found: 381,1377.

3. Enzyme kinetics study:

Activation of cathepsin C from bovine spleen: cathepsin C in a solution 1% NaCl containing 1 mM EDTA-Na_2 and 5 mM β-mercaptoethanol was incubated for half an hour in a water bath at 37 °C.

The enzyme reaction was carried out in acetate buffer (0.1 M, pH 5.0, which contained 30 mM NaCl, 1 mM EDTA-Na_2 and 1 mM DTT (all concentrations given are final)) at 37°C for 10 minutes. The substrate used in the enzymatic reaction was glycine-L-phenylalanine *p*-nitroanilide Gly-L-Phe-pNA, and the progress of the reaction was monitored spectrophotometrically (Jasco V-730) at a wavelength of 405 nm. The reaction mixture consisted of: Gly-L-Phe-pNA substrate solution in 0.1 M acetate buffer pH 5 containing 1 mM EDTA-Na_2, 1 mM DTT, 30 mM NaCl (substrate concentration in the sample: 2.5 to 0 .5 mM), a solution of the potential inhibitor in buffer (concentration depending on the potency of the inhibition) and enzyme (5 nM). Kinetic constants and type of inhibition was calculated using a computer program made available courtesy of PhD J. Hurek (University of Opole). Weighted regression was used in the calculation for linearizations. All measurements were performed in triplicate.

4. NMR data

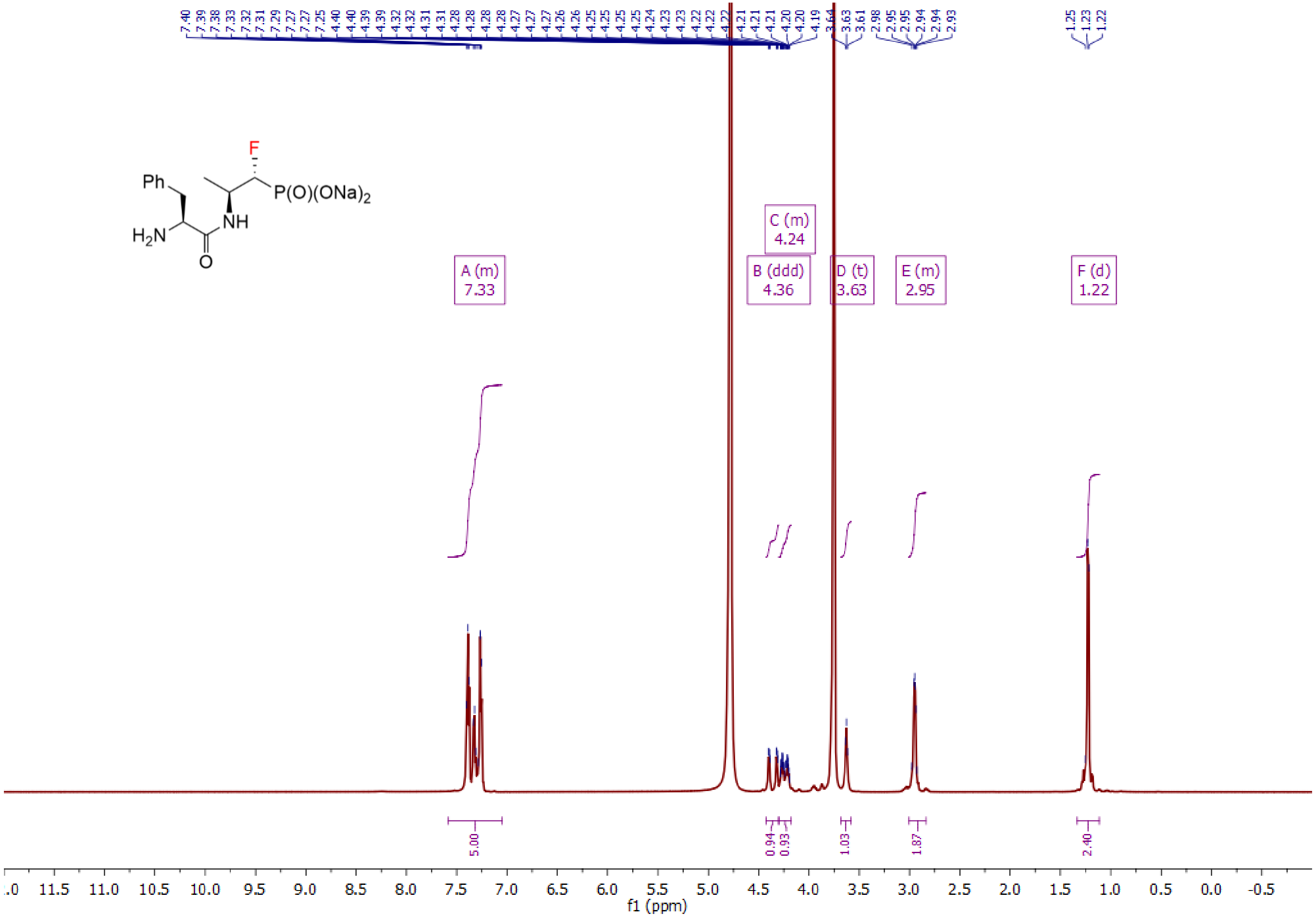

^{1}H NMR (600 MHz, D_2O) of **9a**.

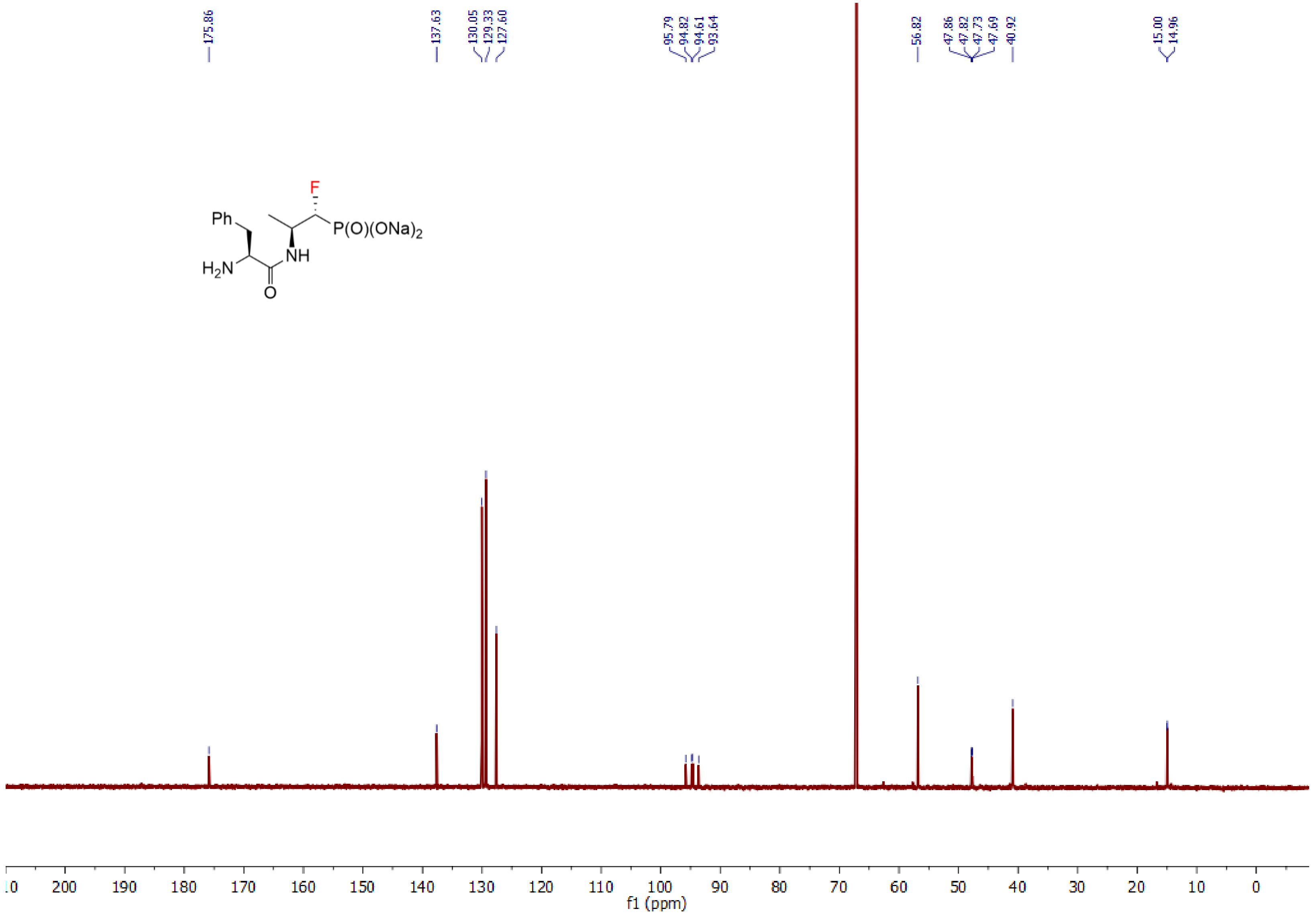

^{13}C NMR (151 MHz, D_2O) of **9a**.

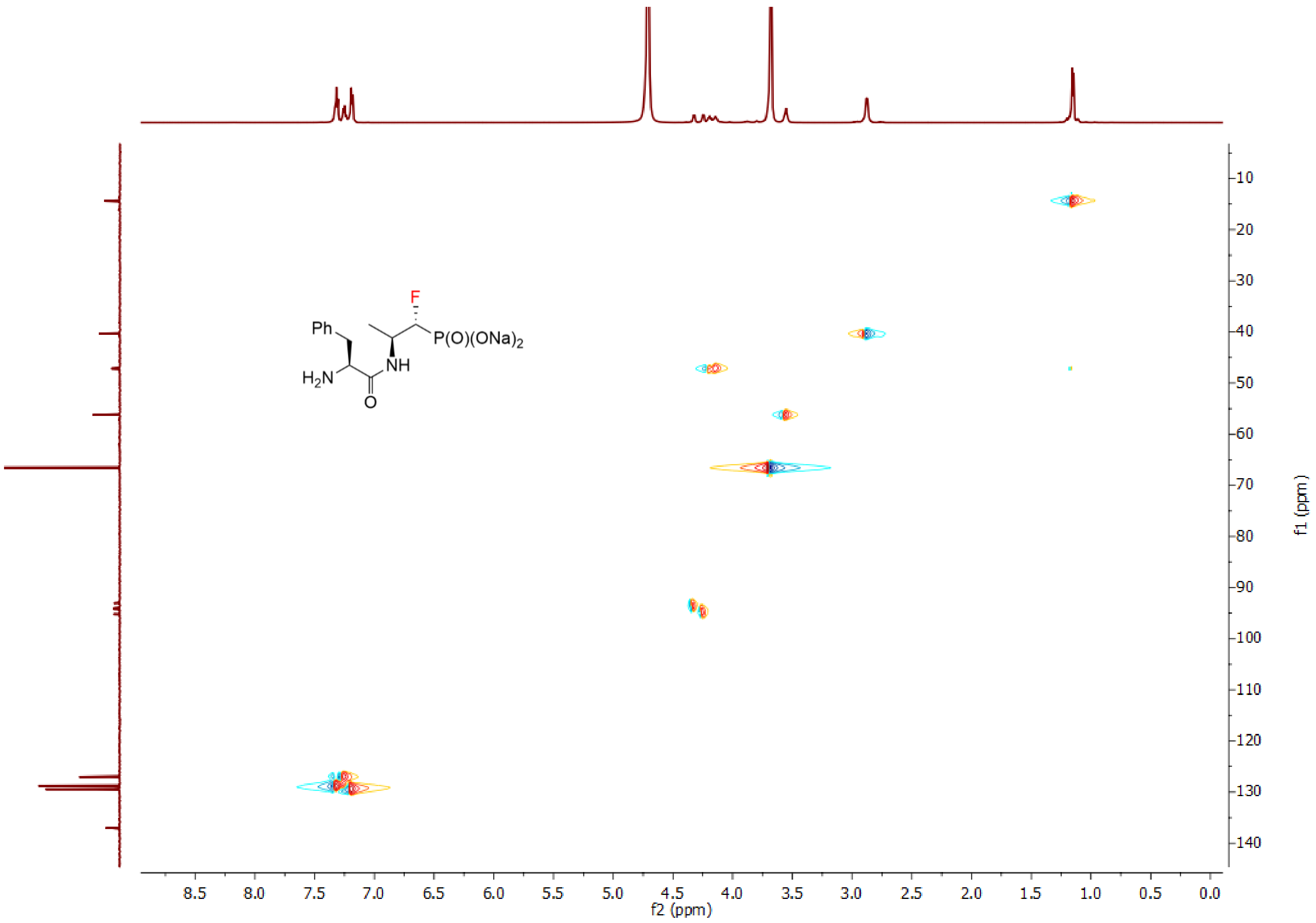

^{1}H-^{13}C HSQC (600 MHz, D_2O) of **9a**.

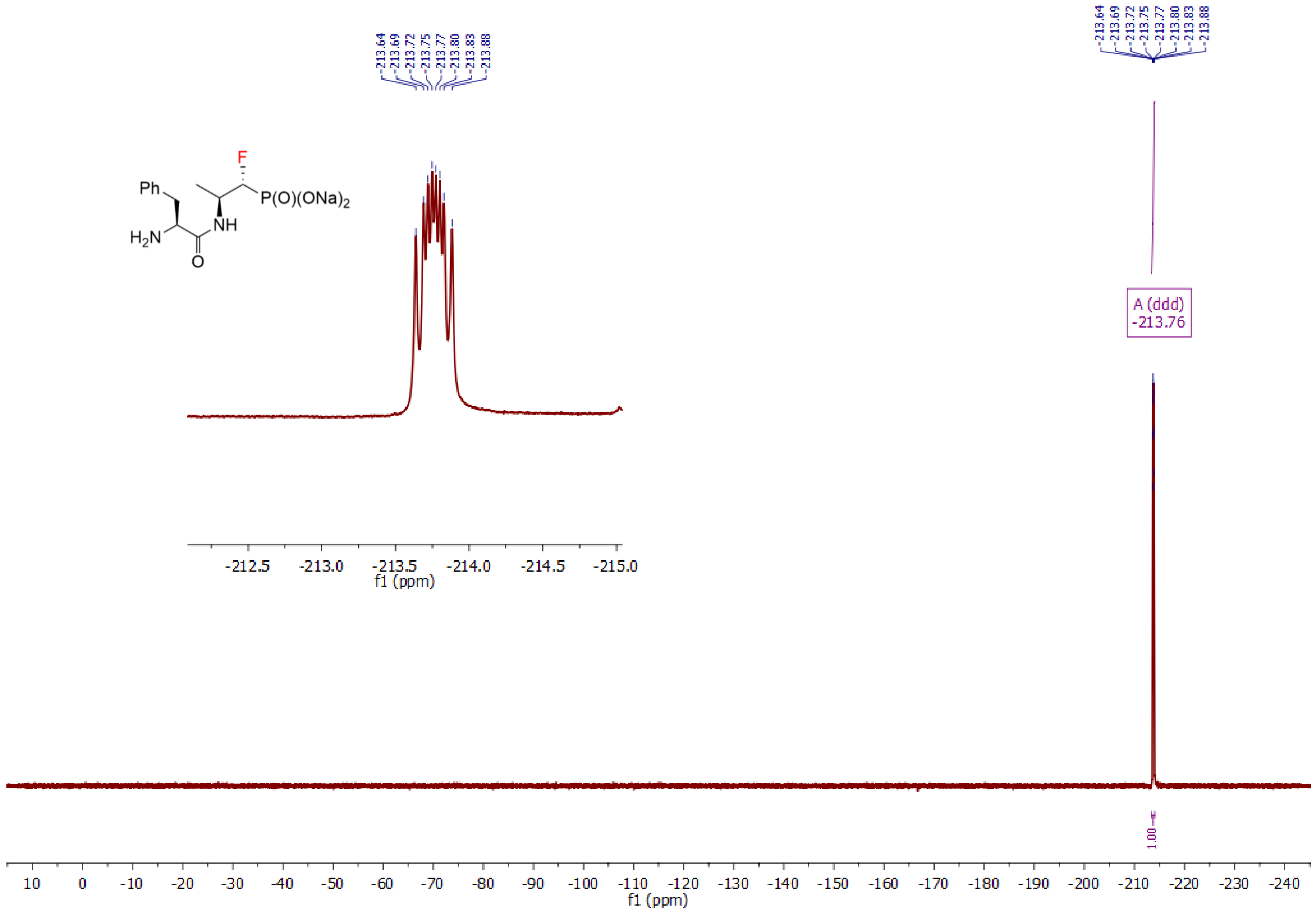

^{19}F NMR (565 MHz, D_2O) of **9a**.

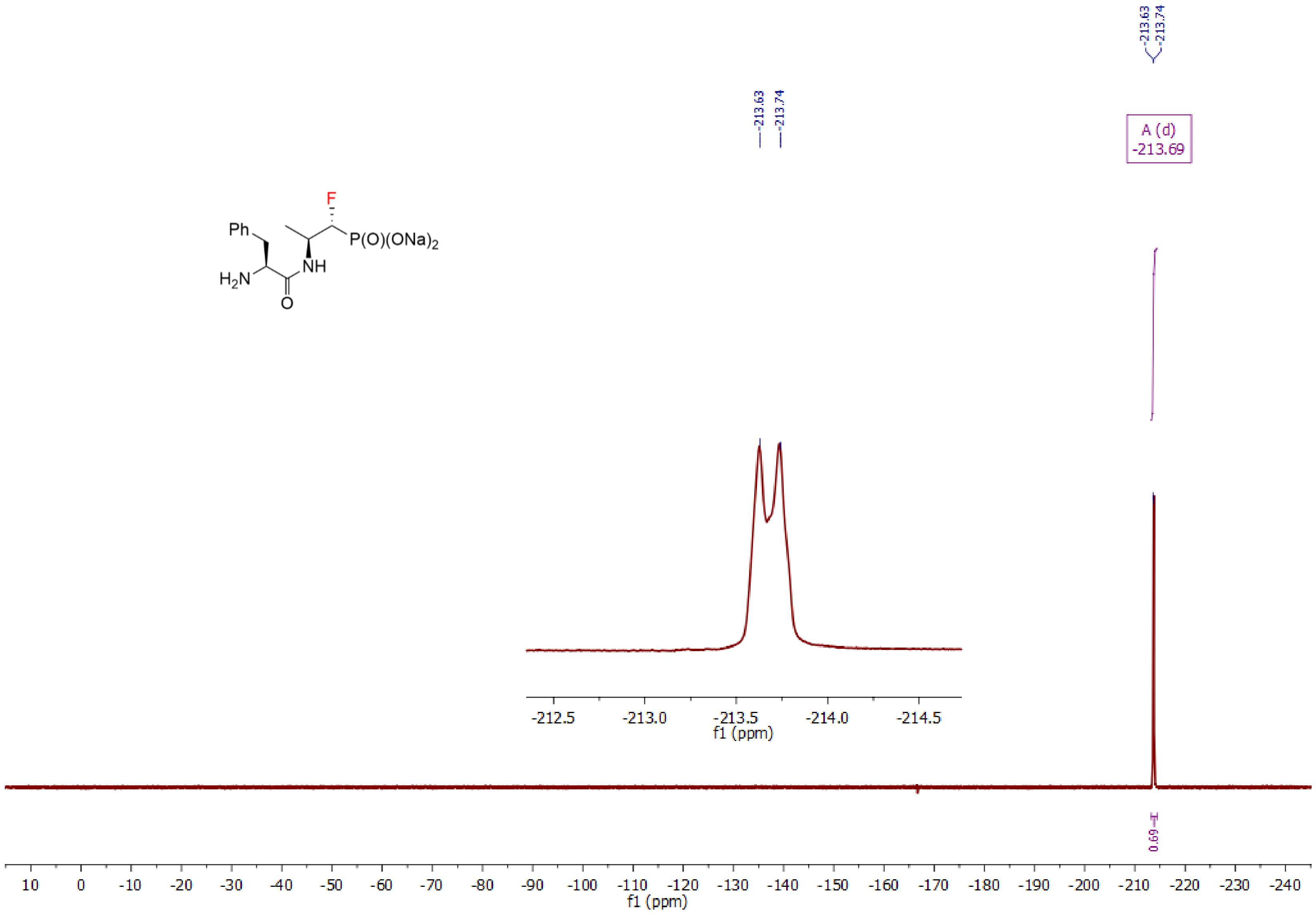

$^{19}F\{/^{1}H\}$ NMR (565 MHz, D_2O) of **9a**.

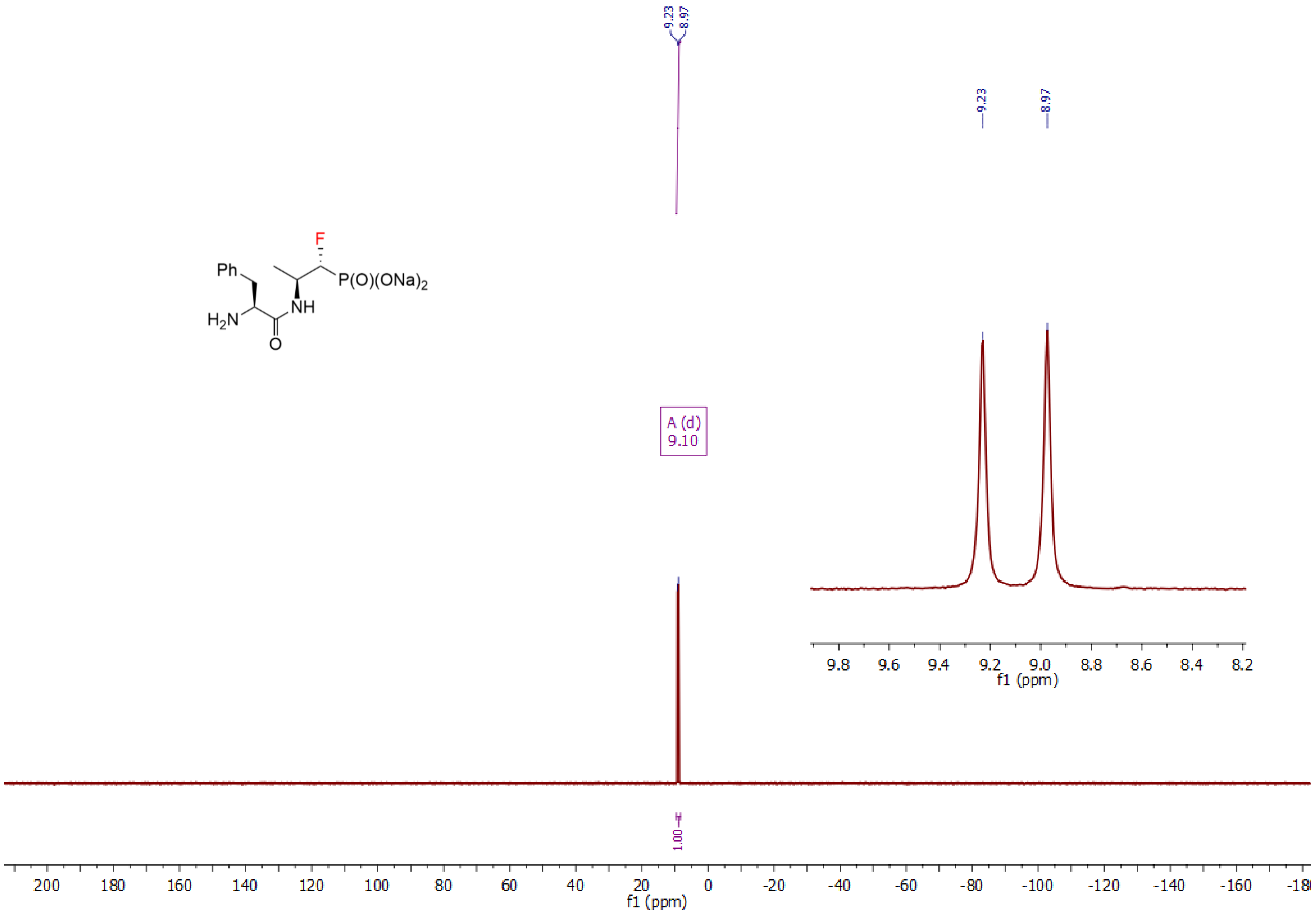

$^{31}P\{/^{1}H\}$ NMR (243 MHz, D_2O) of **9a**.

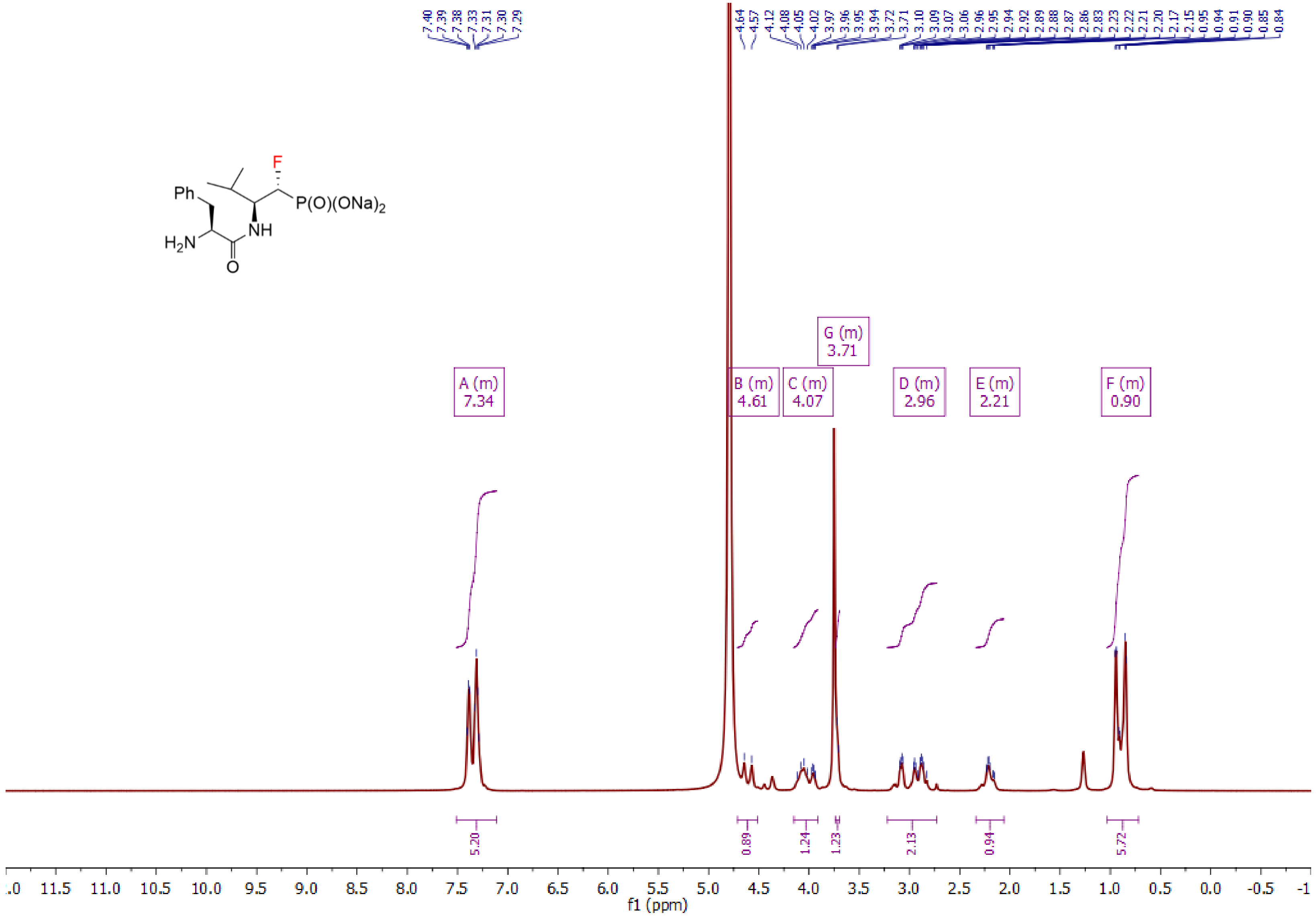

^{1}H NMR (600 MHz, D_2O) of **9b**.

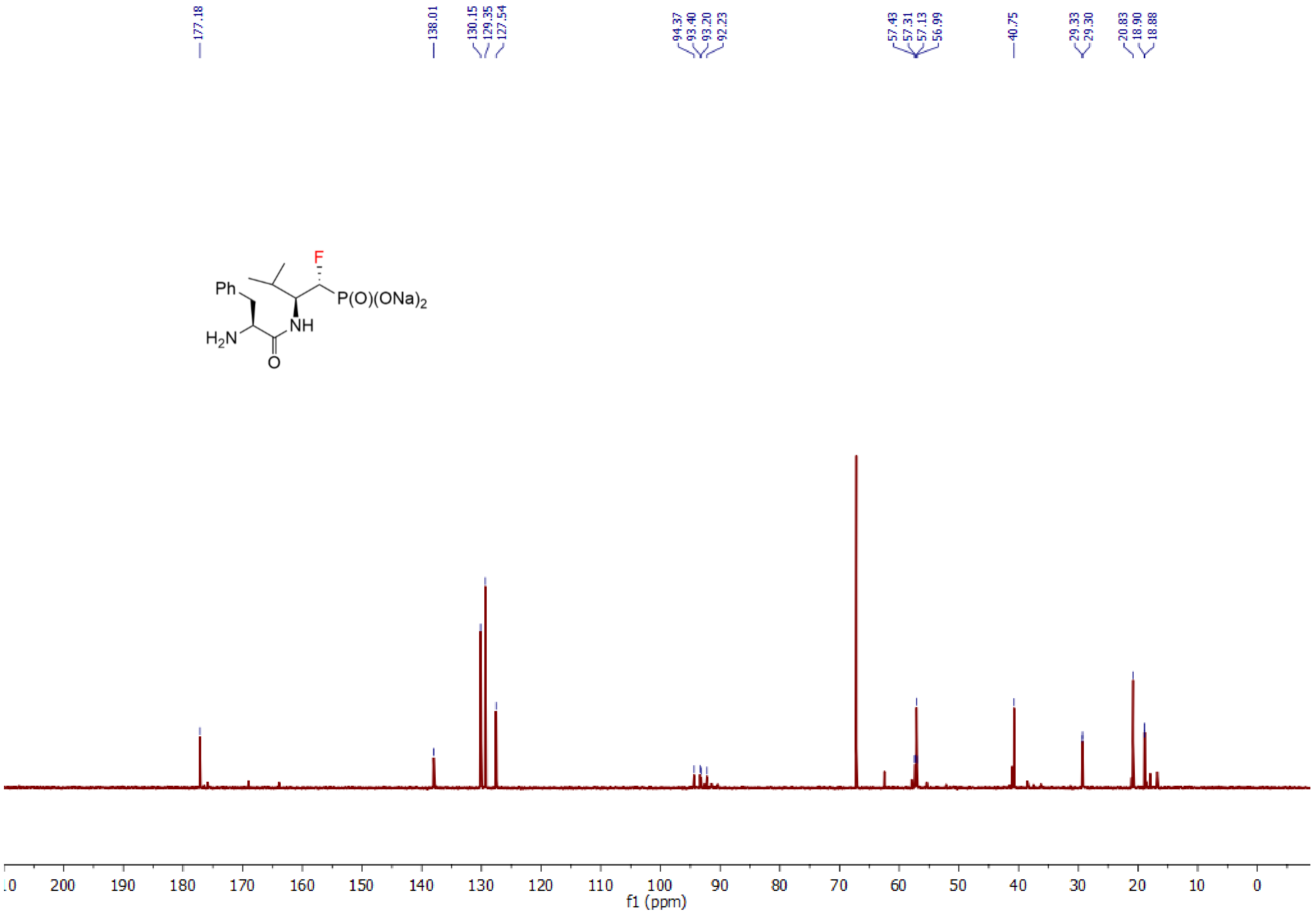

^{13}C NMR (151 MHz, D_2O) of **9b**.

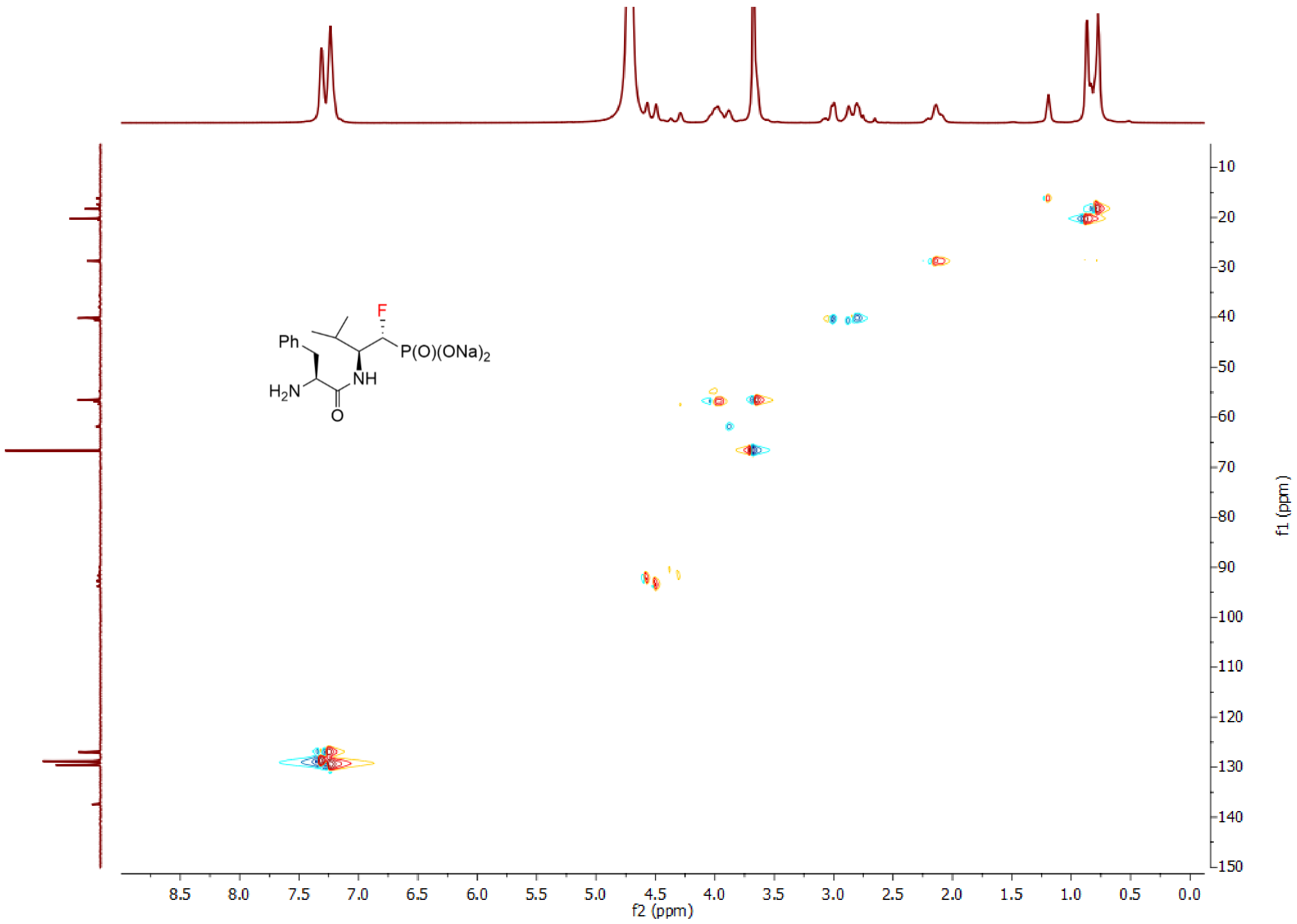

^{1}H-^{13}C HSQC (600 MHz, D_2O) of **9b**.

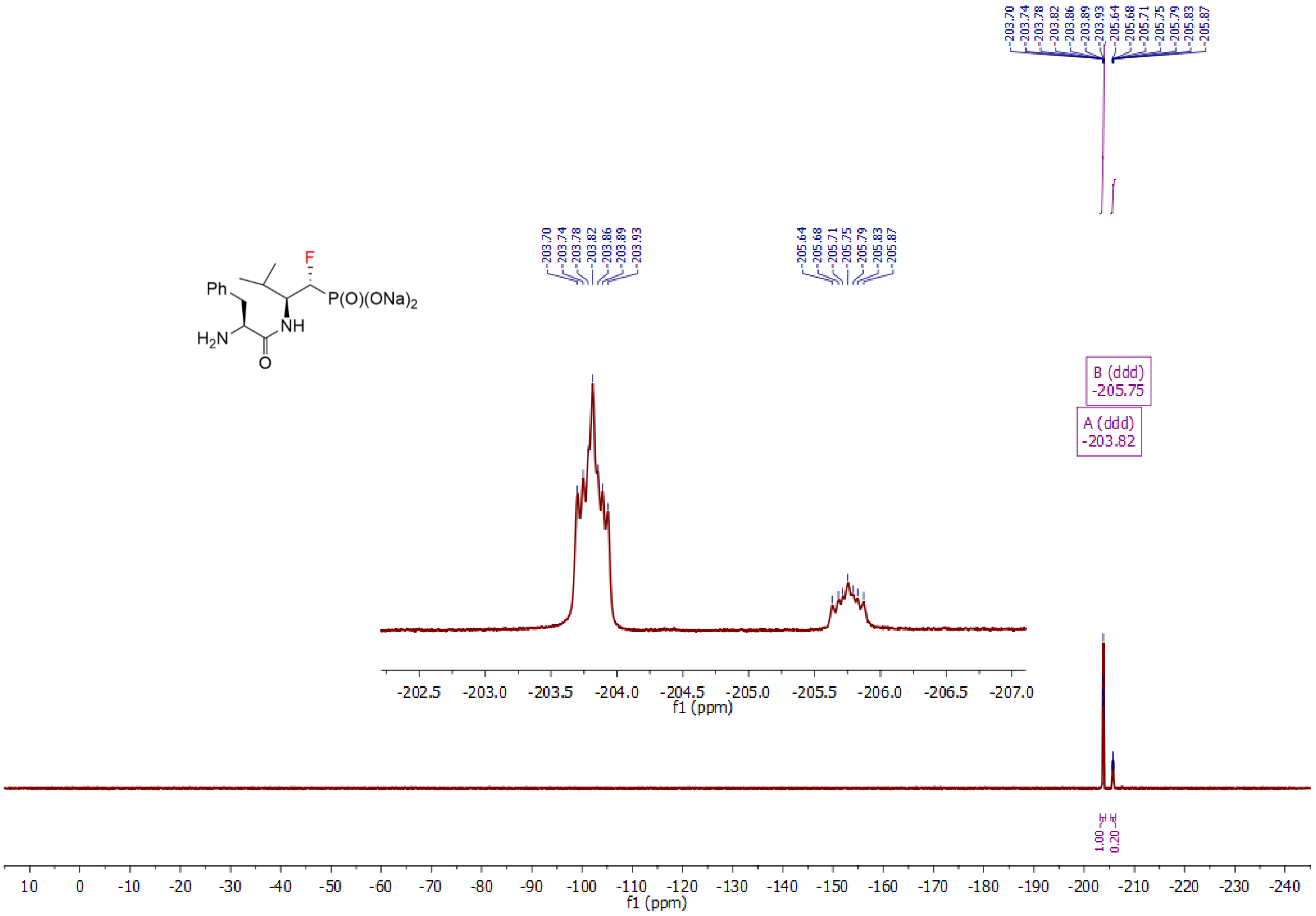

^{19}F NMR (565 MHz, D_2O) of **9b**.

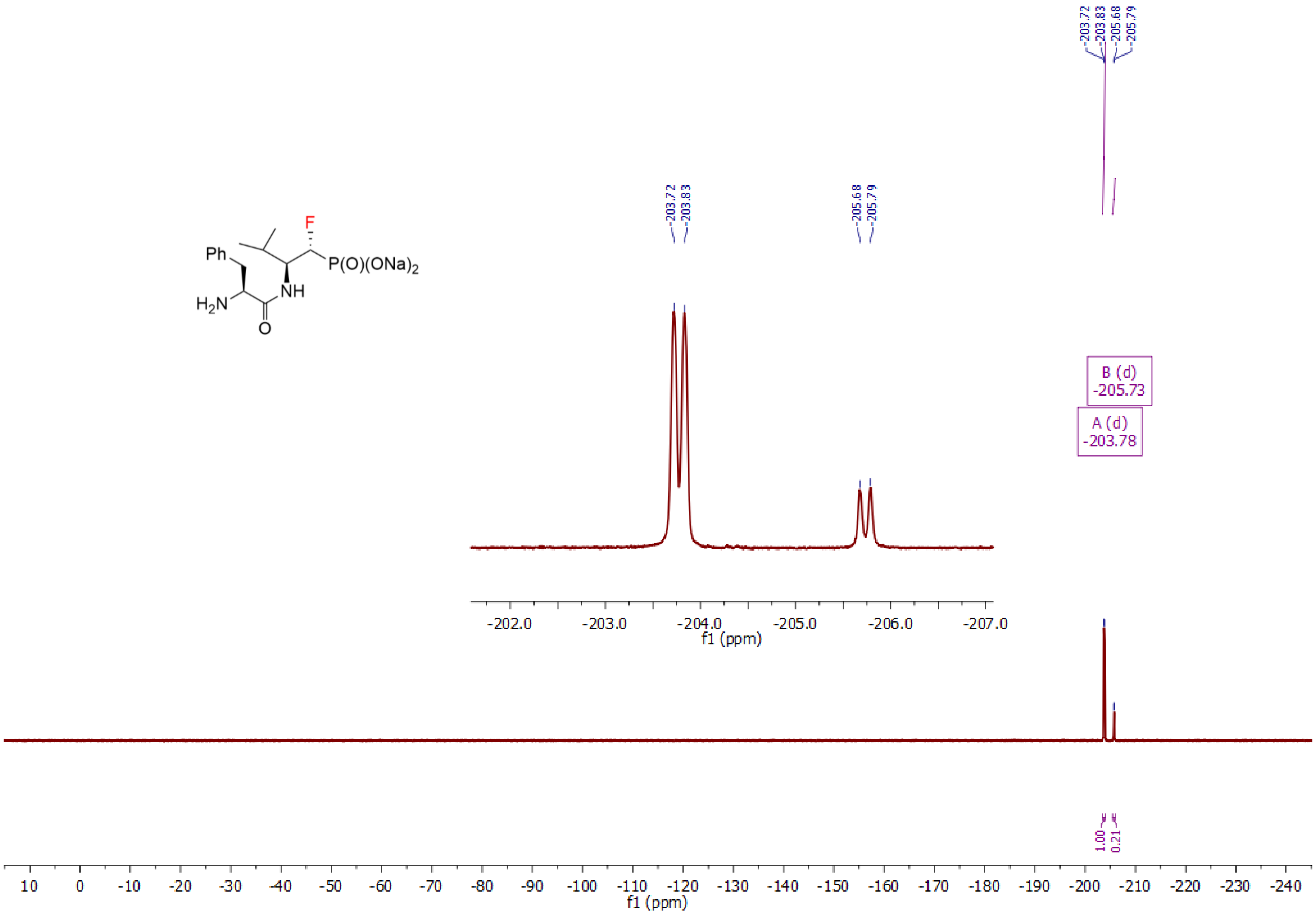

$^{19}F\{/^{1}H\}$ NMR (565 MHz, D_2O) of **9b**.

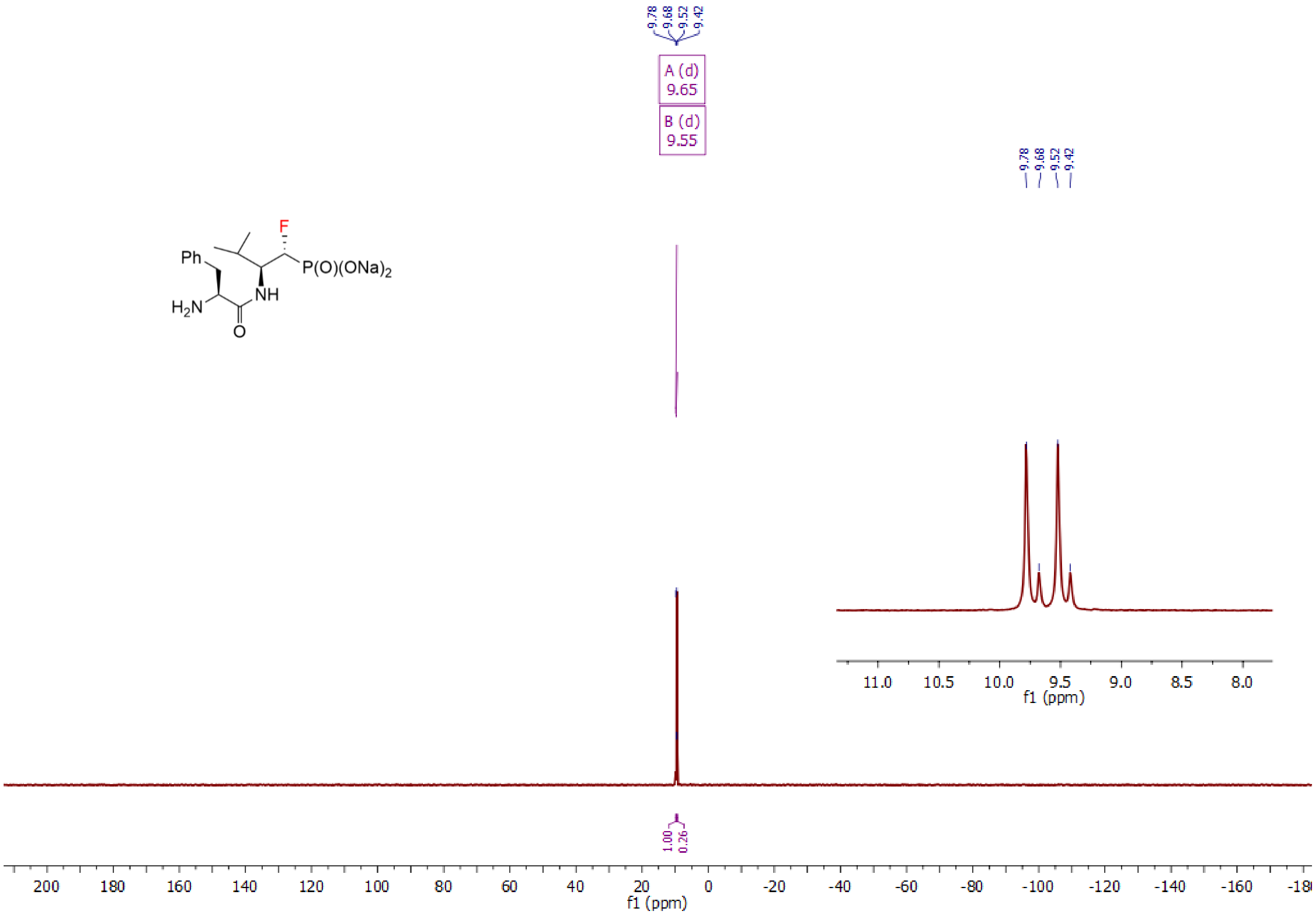

$^{31}P\{/^{1}H\}$ NMR (243 MHz, D_2O) of **9b**.

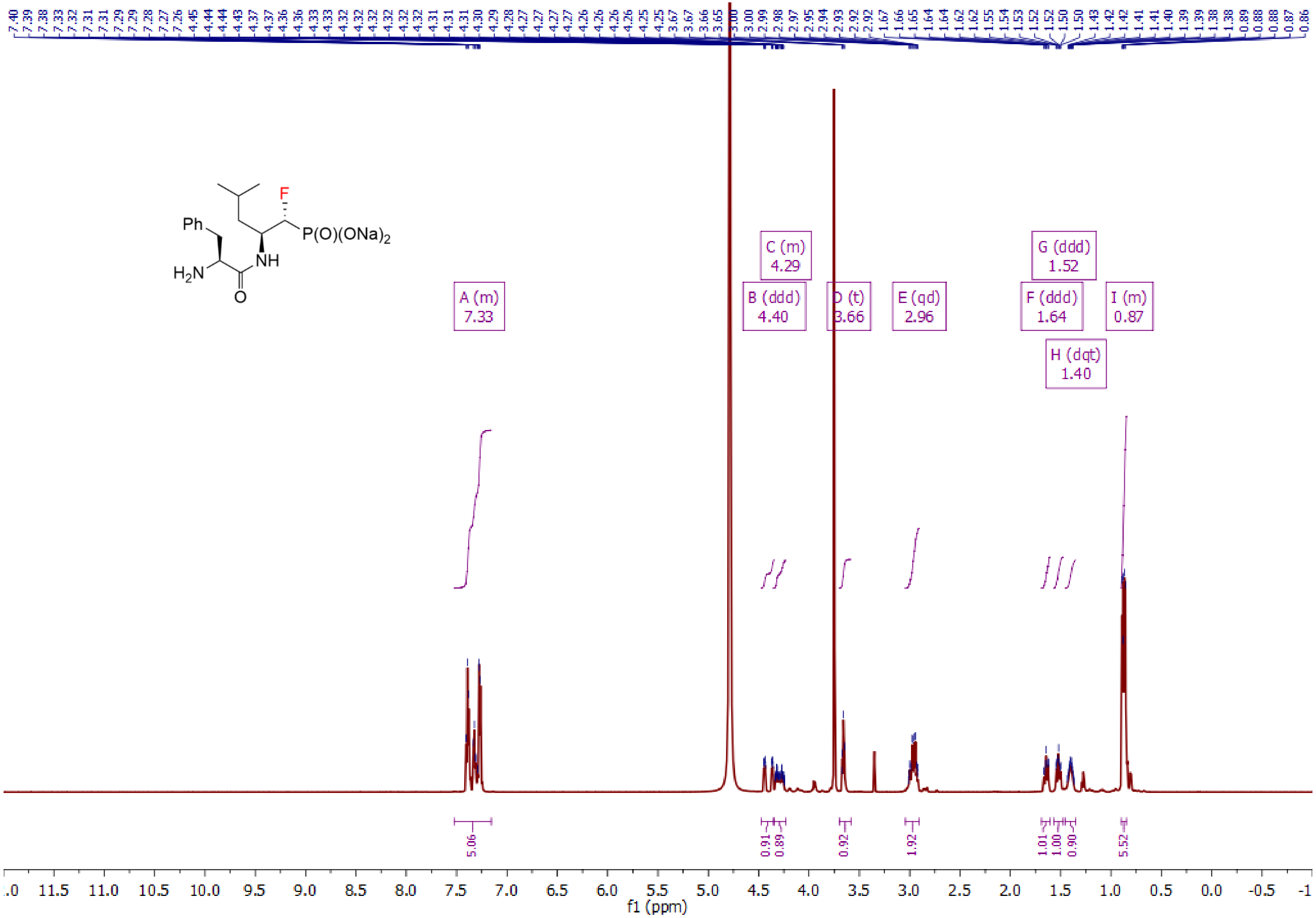

^{1}H NMR (600 MHz, D_2O) of **9c**.

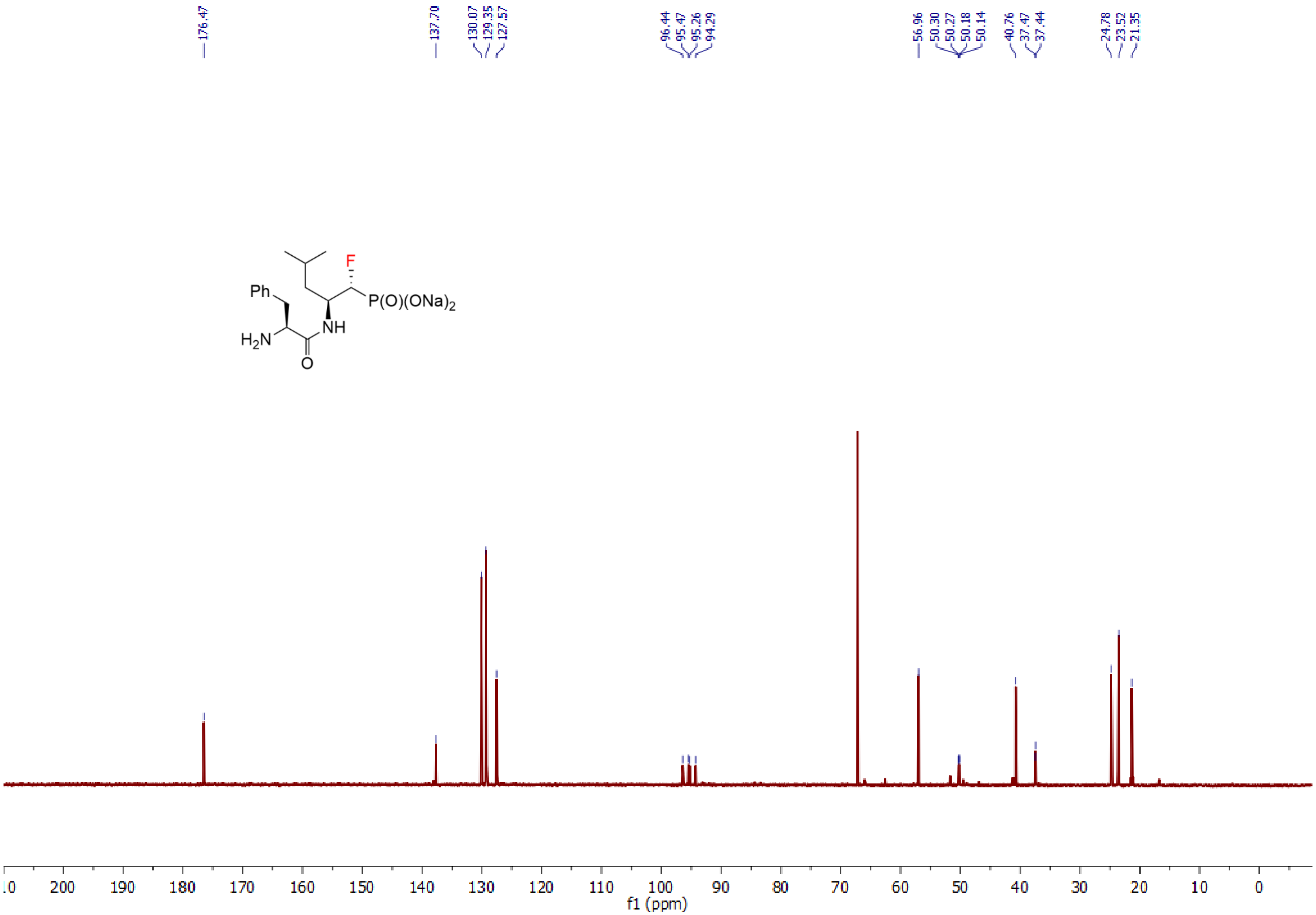

^{13}C NMR (151 MHz, D_2O) of **9c**.

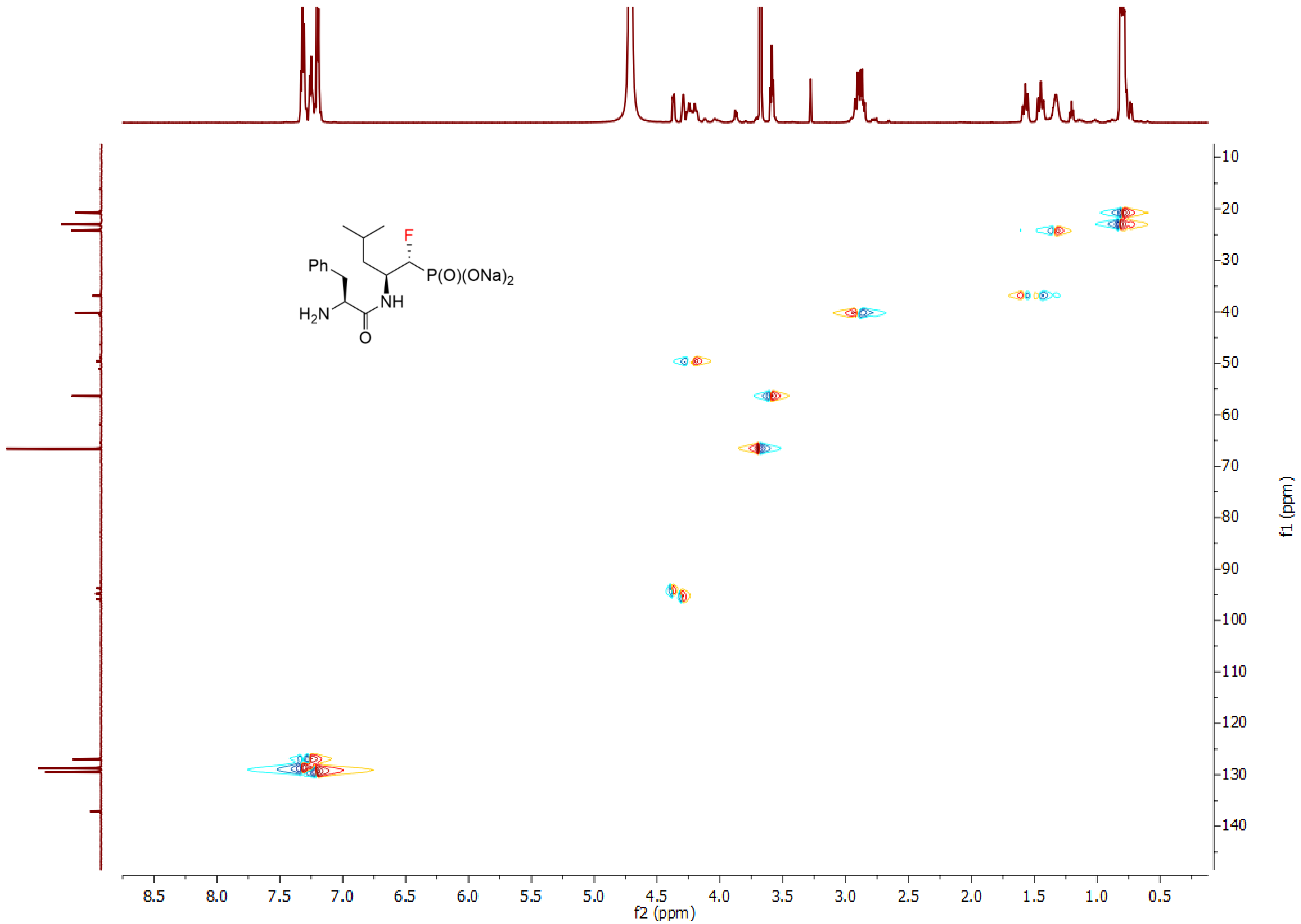

^{1}H-^{13}C HSQC (600 MHz, D_2O) of **9c**.

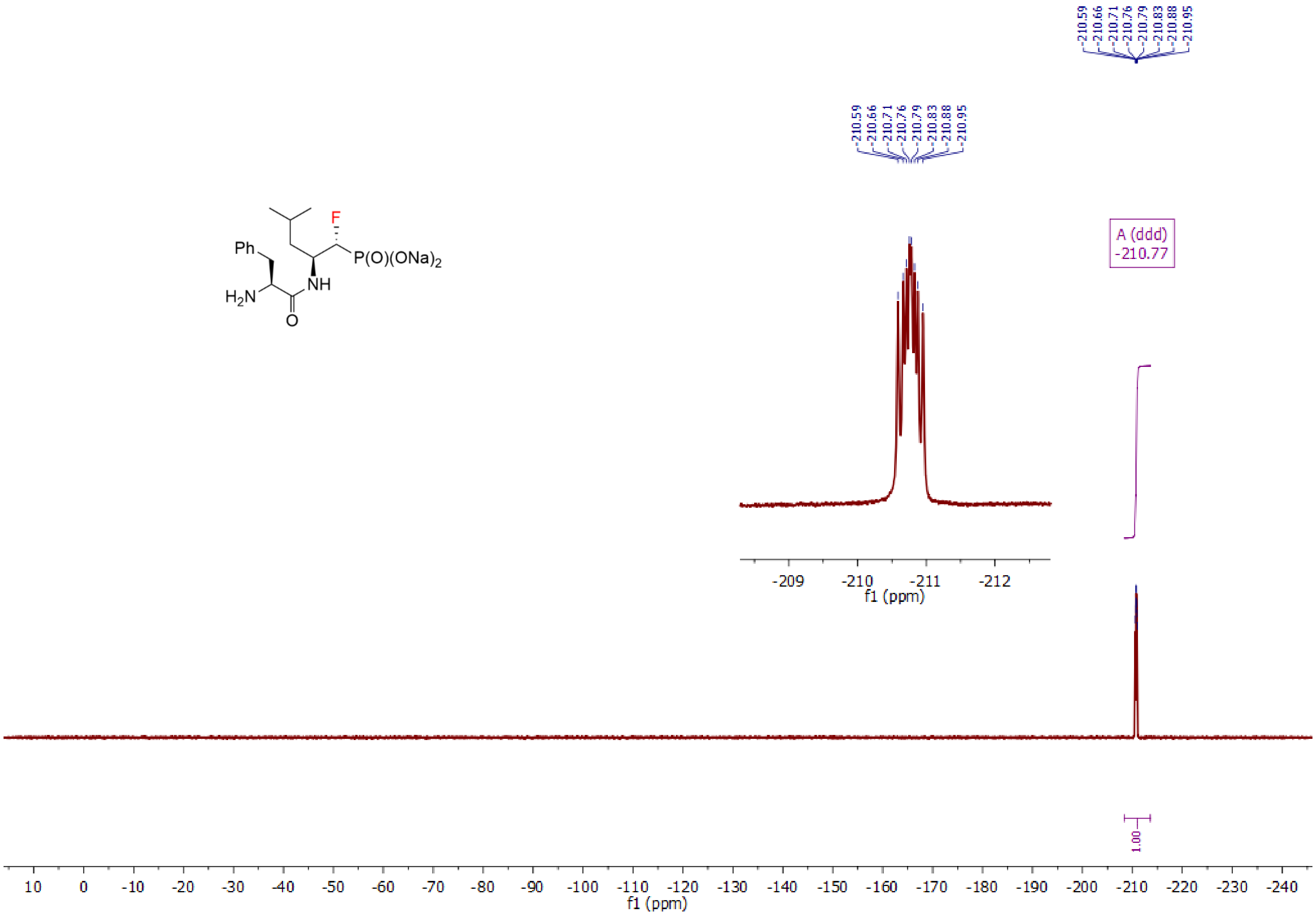

^{19}F NMR (376 MHz D_2O) of **9c**.

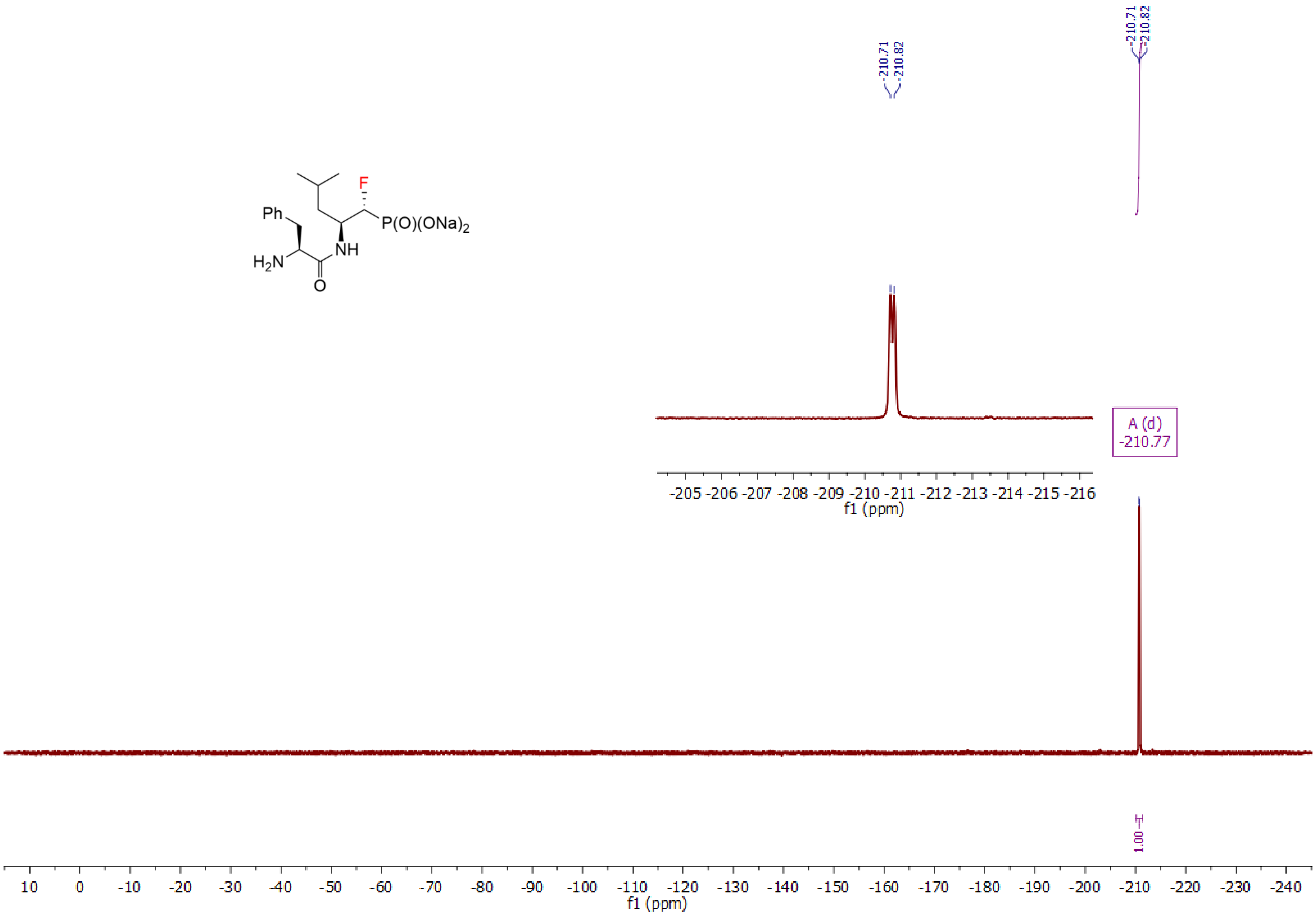

$^{19}F\{/^{1}H\}$ NMR (565 MHz, D_2O) of **9c**.

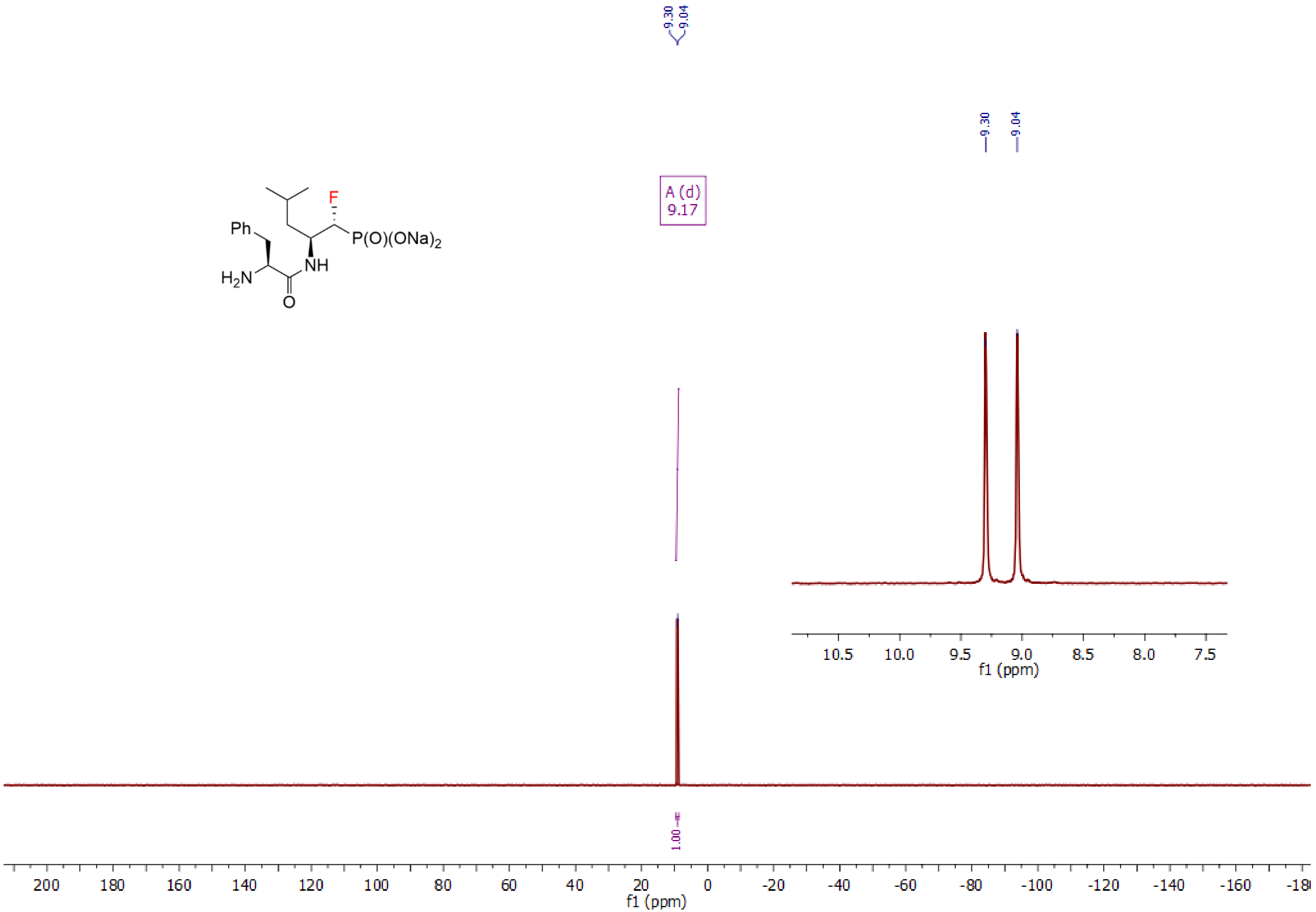

$^{31}P\{/^{1}H\}$ NMR (243 MHz, D_2O) of **9c**.

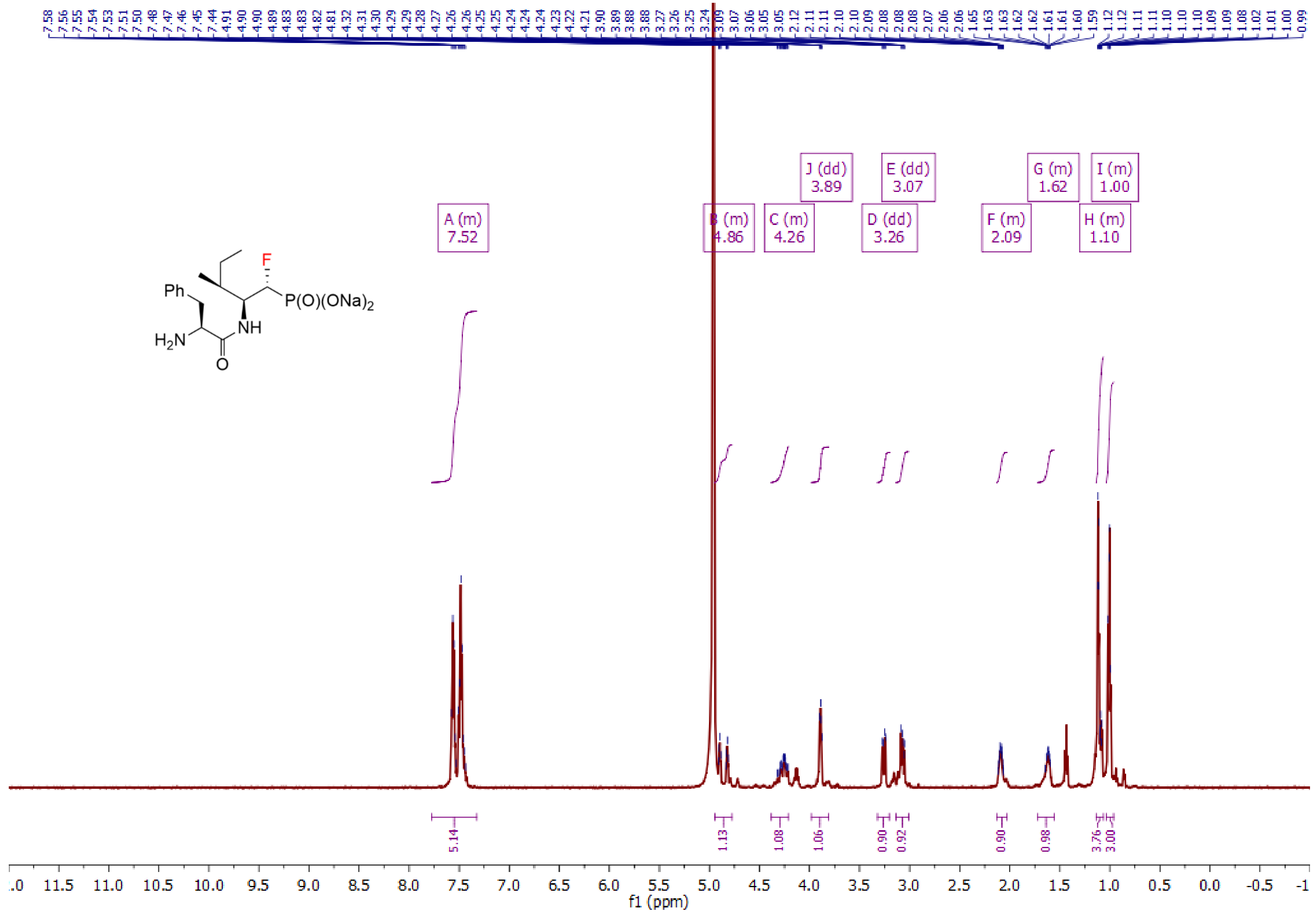

^{1}H NMR (600 MHz, D_2O) of **9d**.

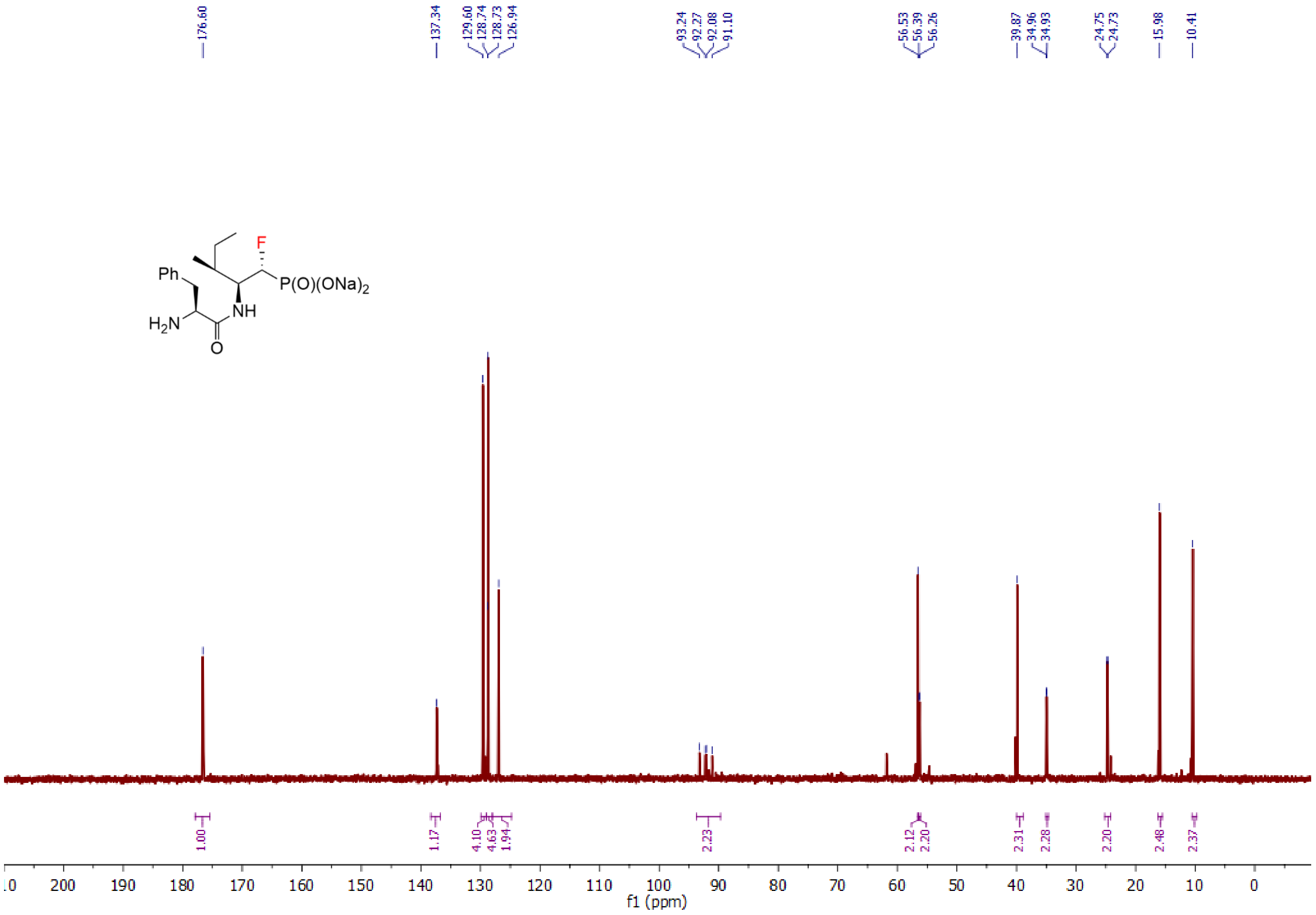

^{13}C NMR (151 MHz, D_2O) of **9d**.

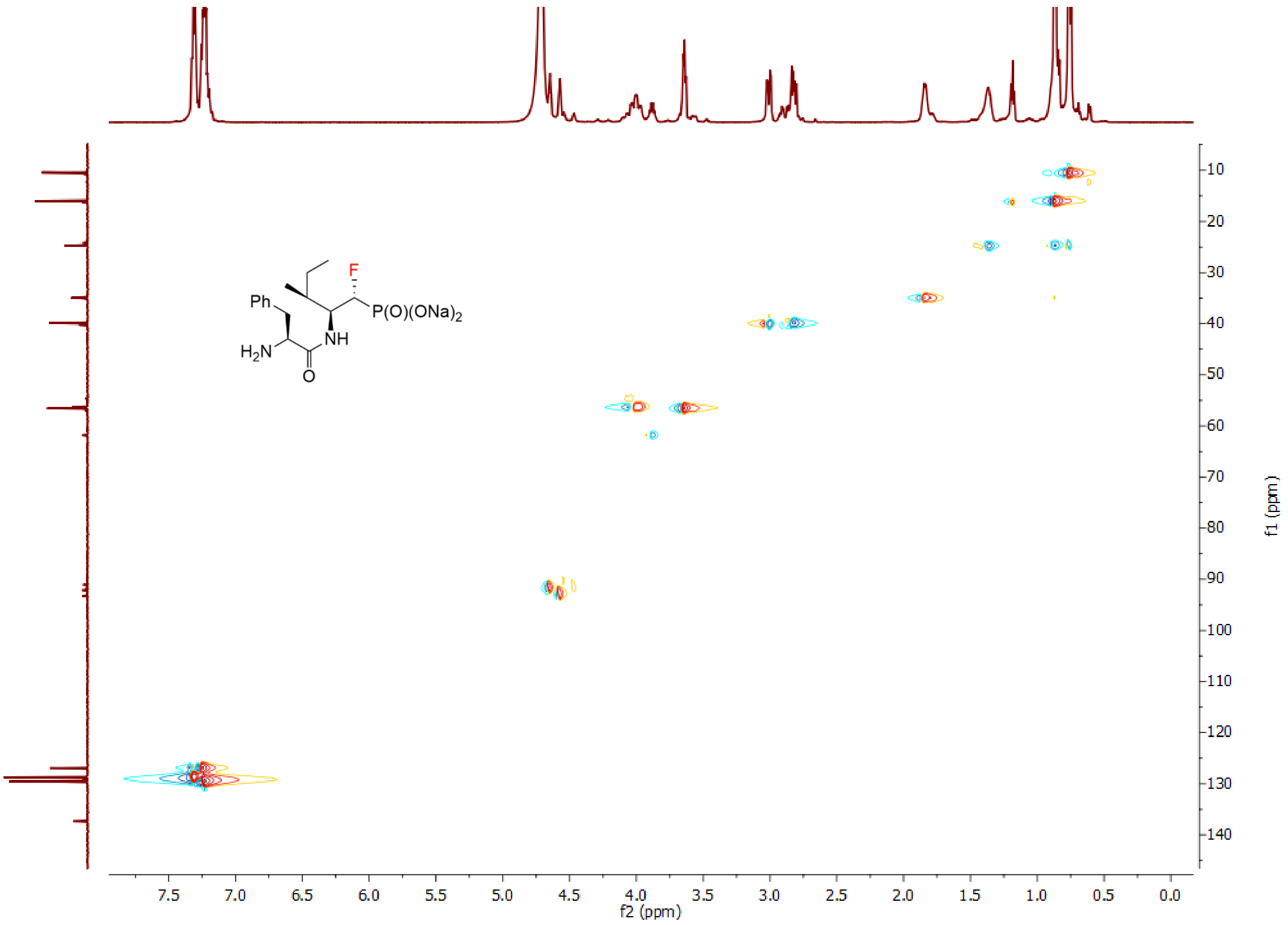

^{1}H-^{13}C HSQC (600 MHz, D_2O) of **9d**.

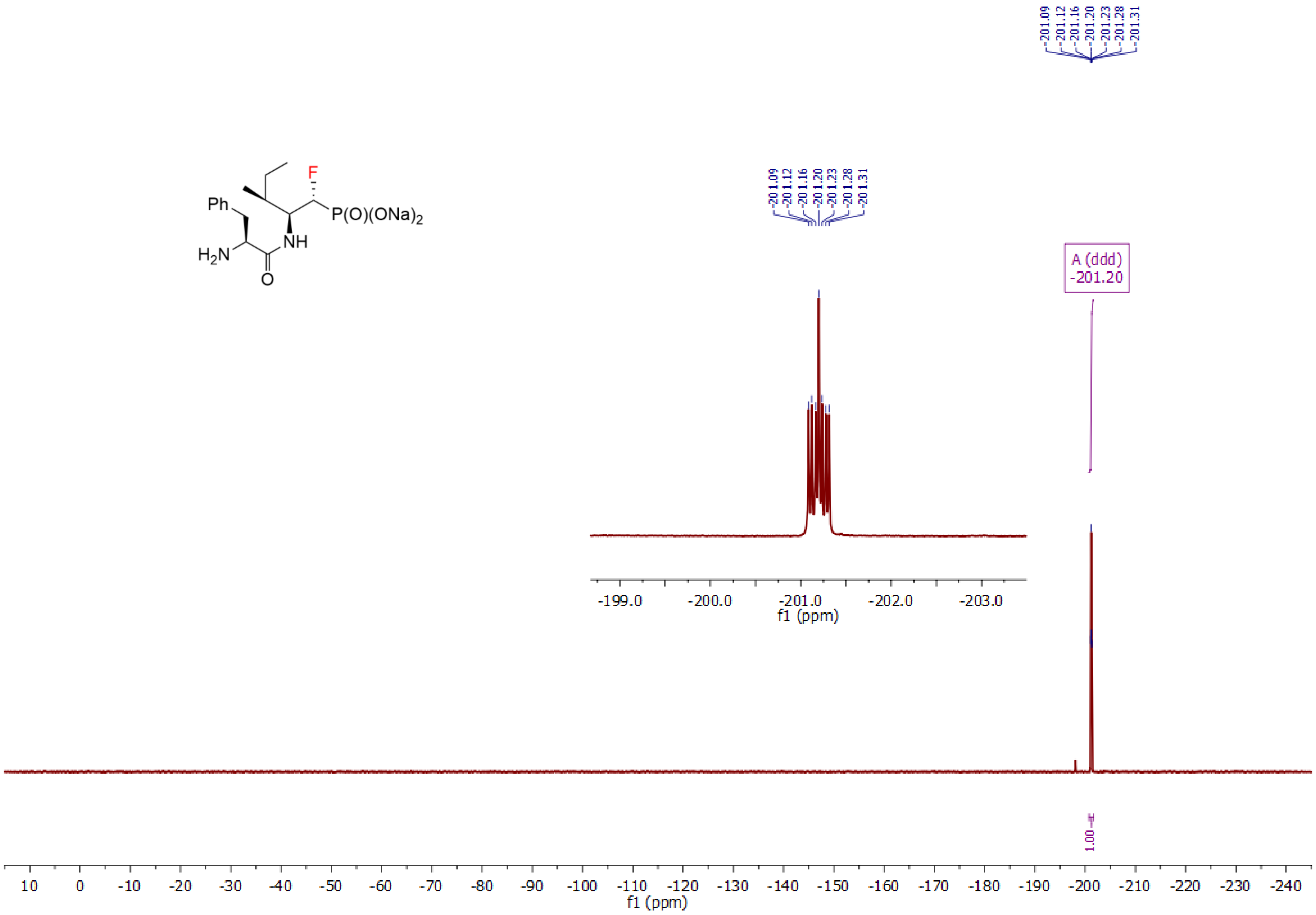

^{19}F NMR (565 MHz, D_2O) of **9d**.

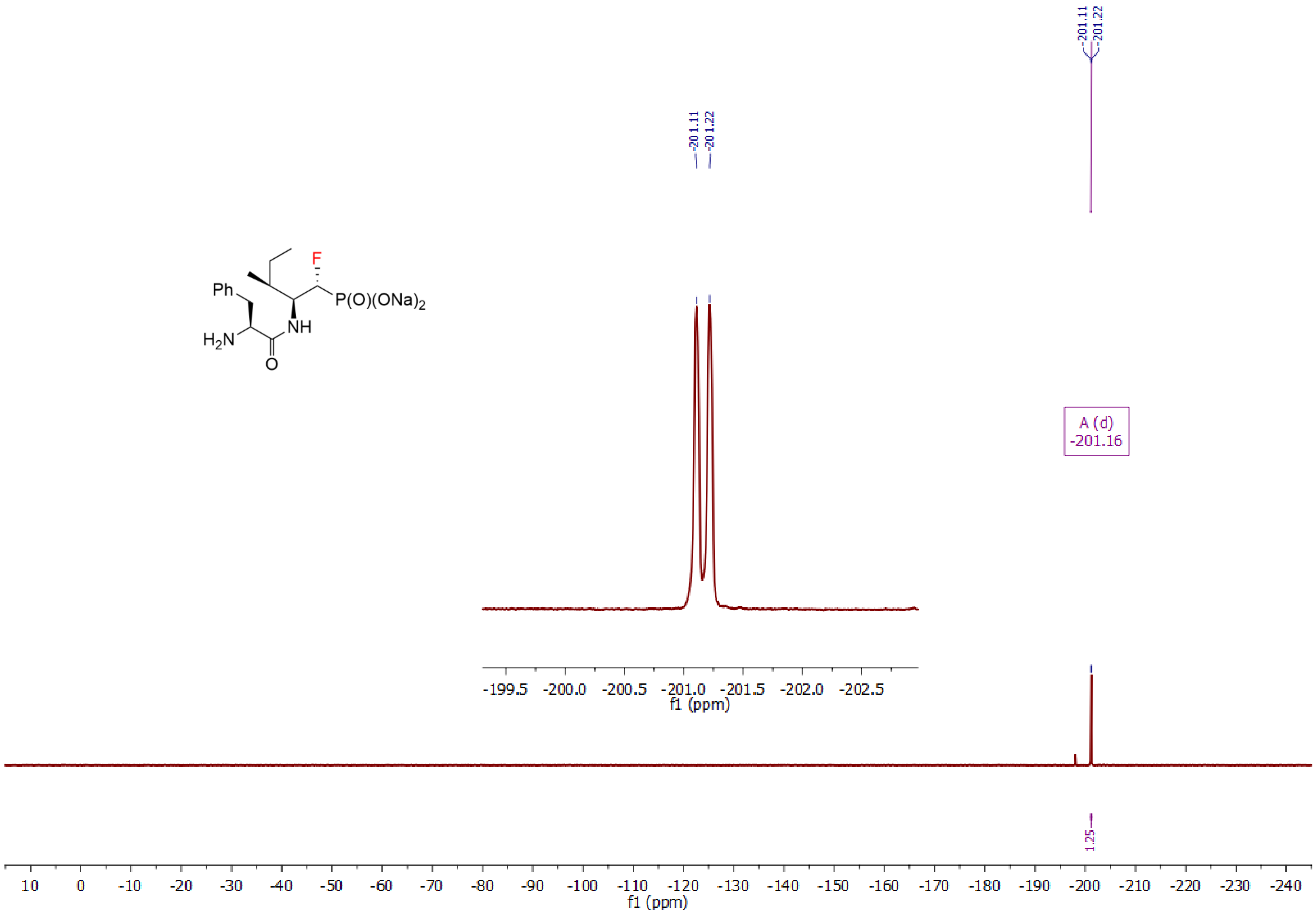

$^{19}F\{/^{1}H\}$ NMR (565 MHz, D_2O) of **9d**.

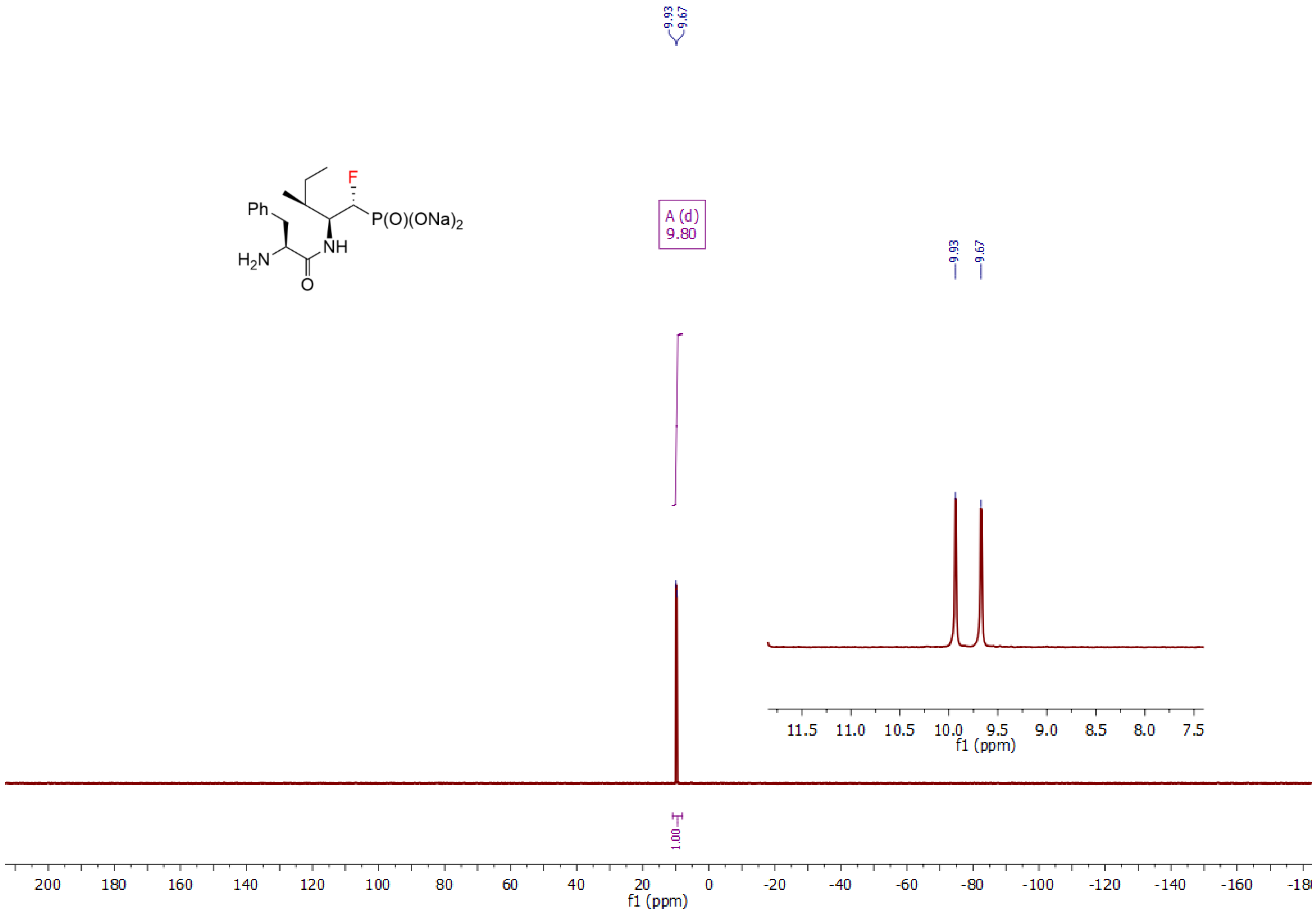

$^{31}P\{/^{1}H\}$ NMR (243 MHz, D_2O) of **9d**.

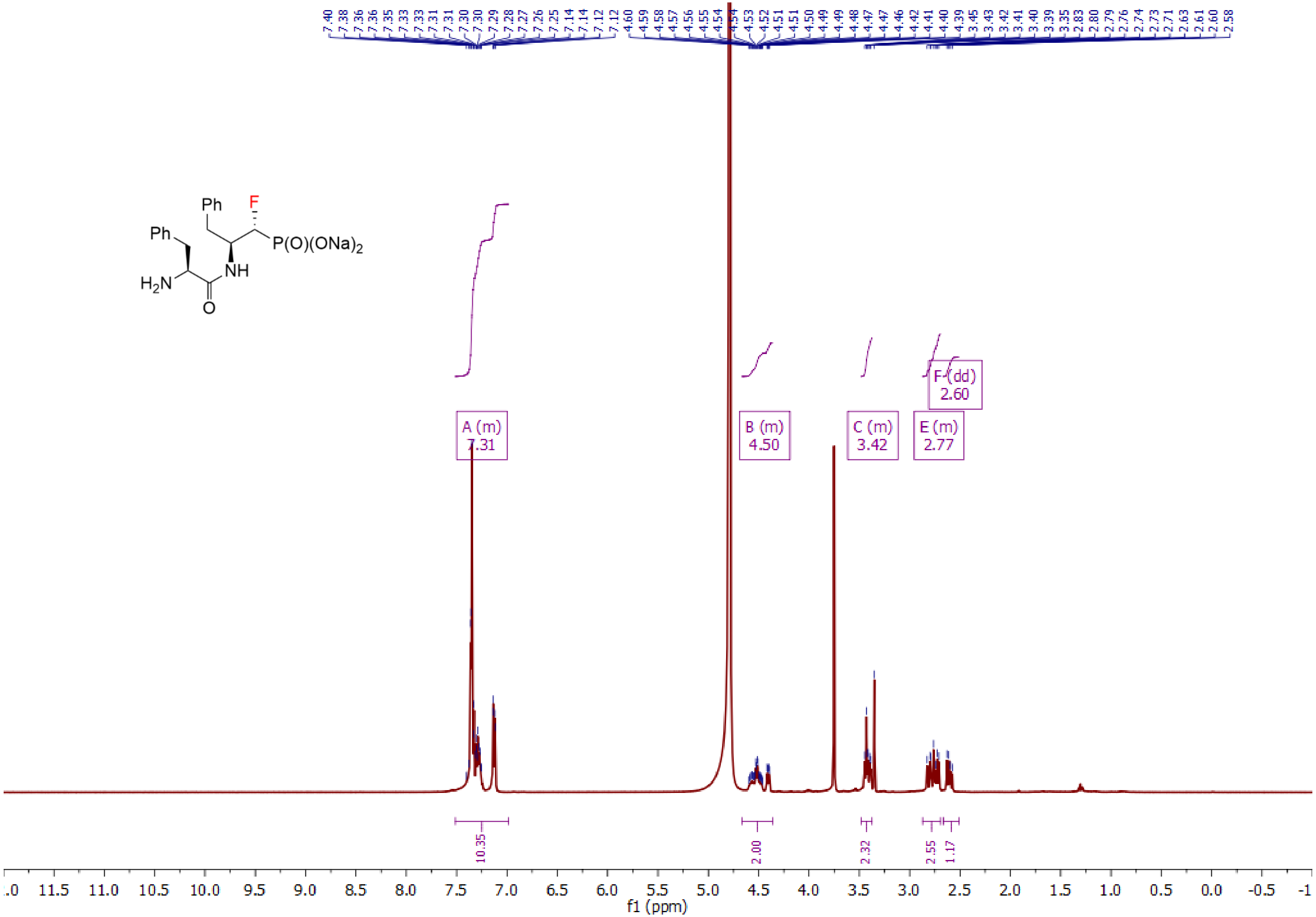

^{1}H NMR (400 MHz, D_2O) of **9e**.

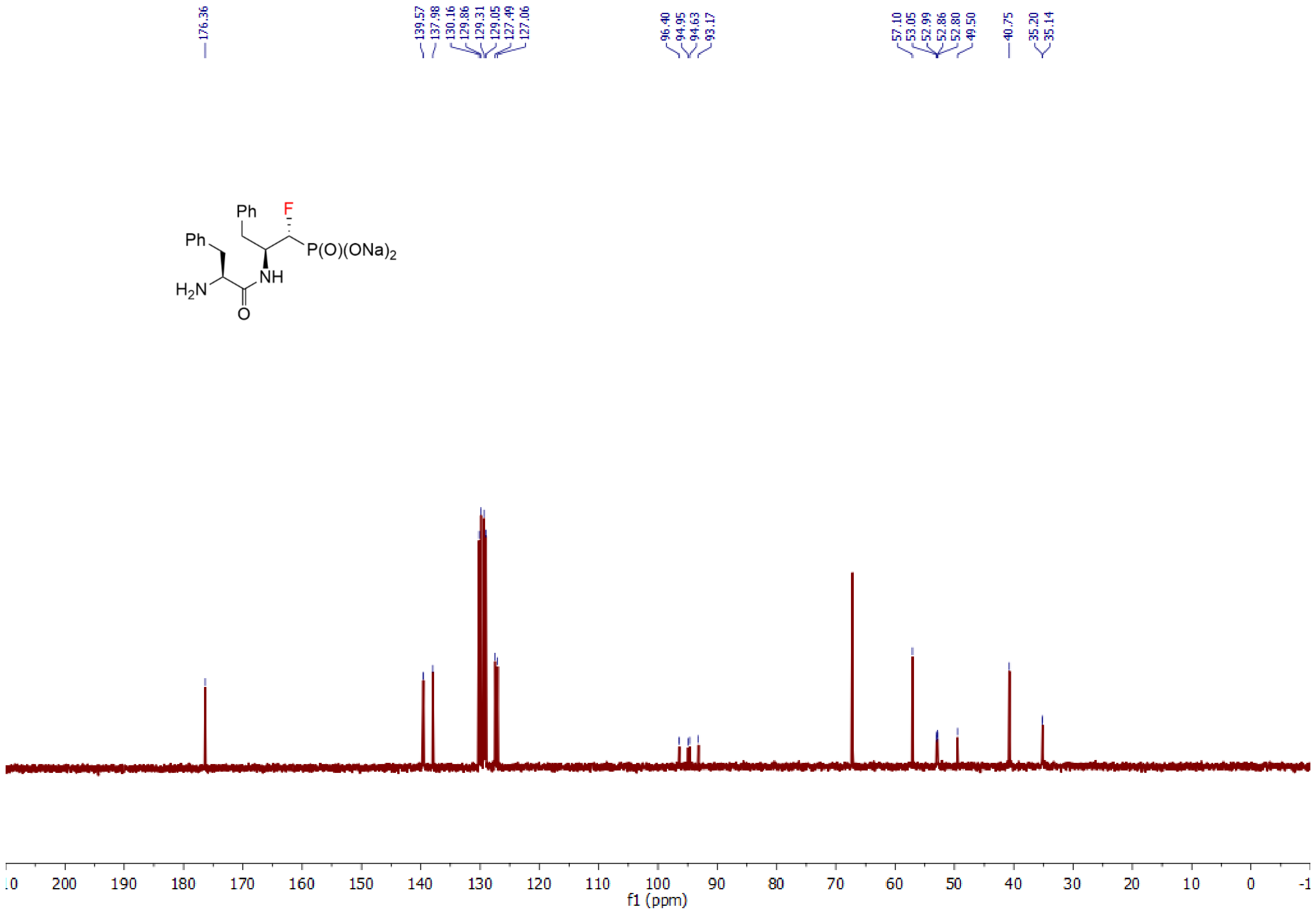

^{13}C NMR (101 MHz, D_2O) of **9e**.

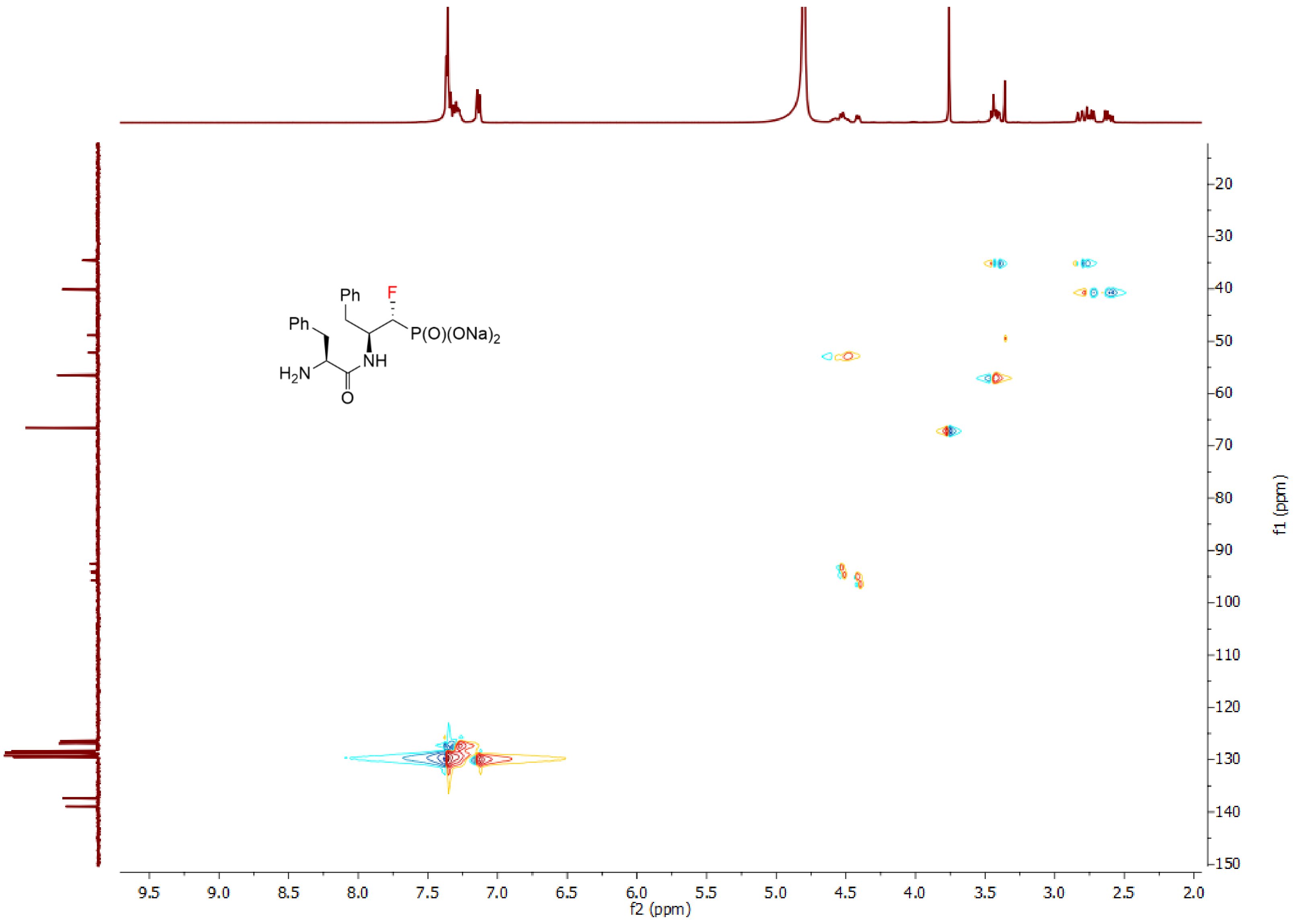

^{1}H-^{13}C HSQC (400 MHz, D_2O) of **9e**.

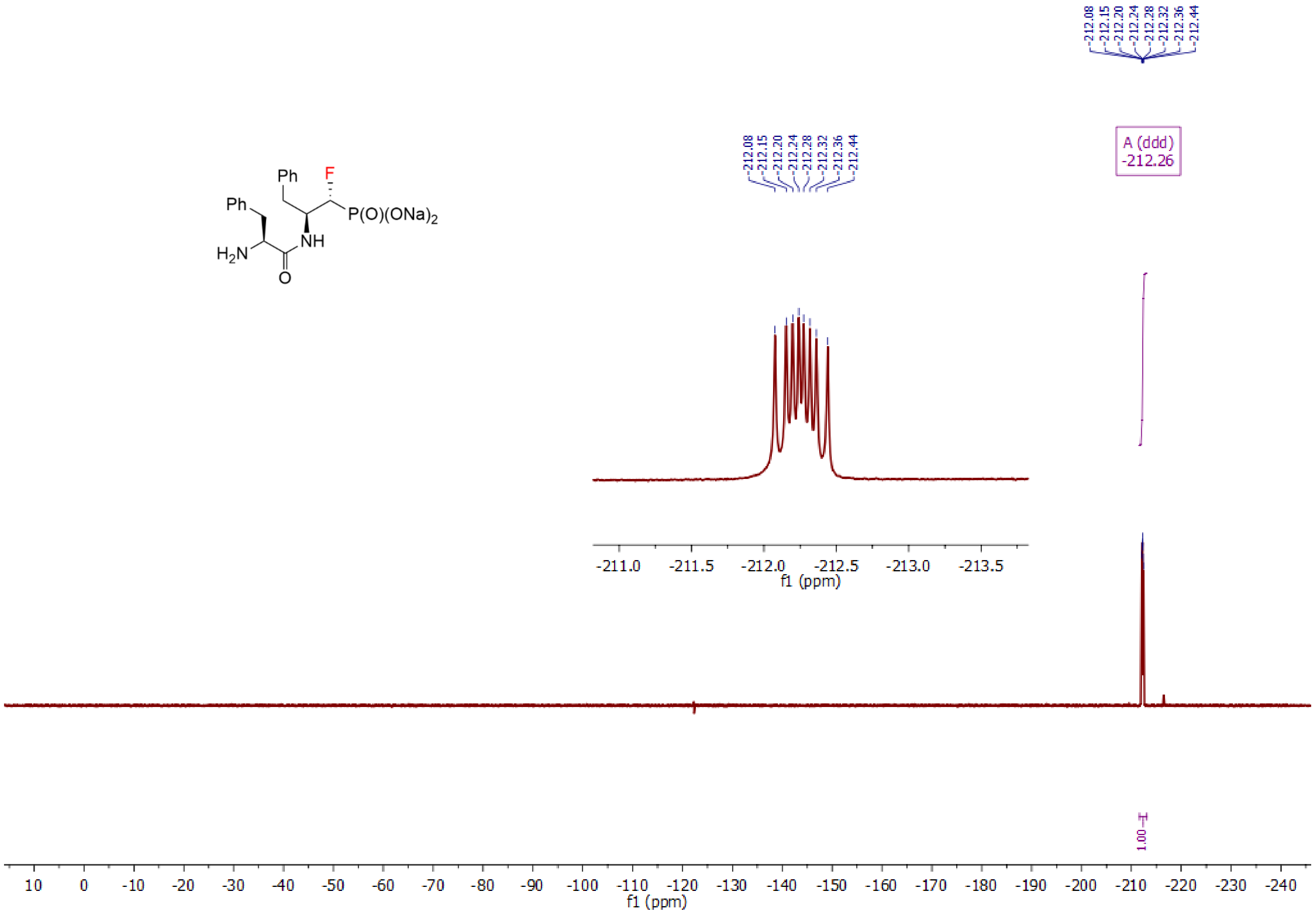

^{19}F NMR (376 MHz D_2O) of **9e**.

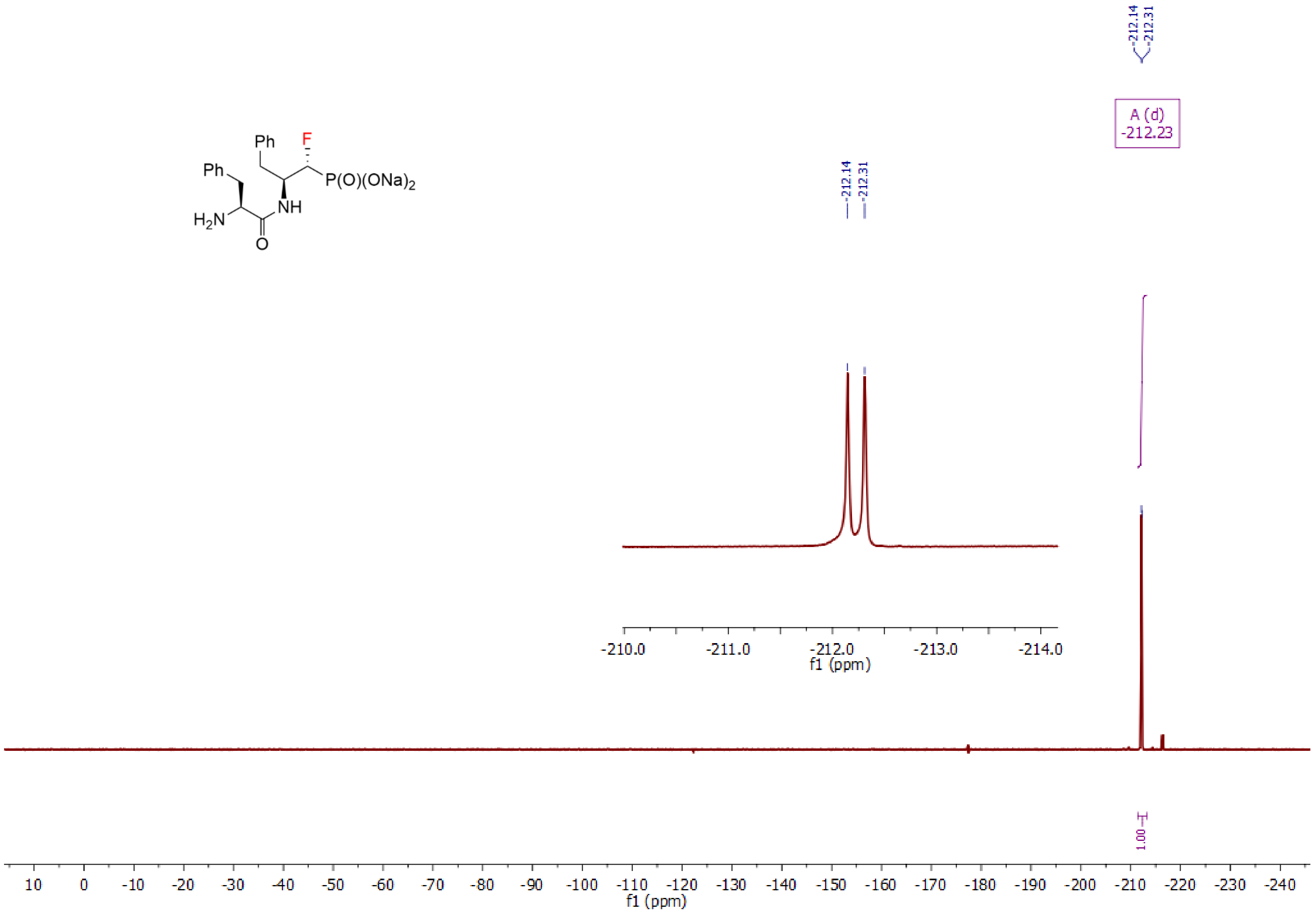

$^{19}F\{/^{1}H\}$ NMR (376 MHz D_2O) of **9e**.

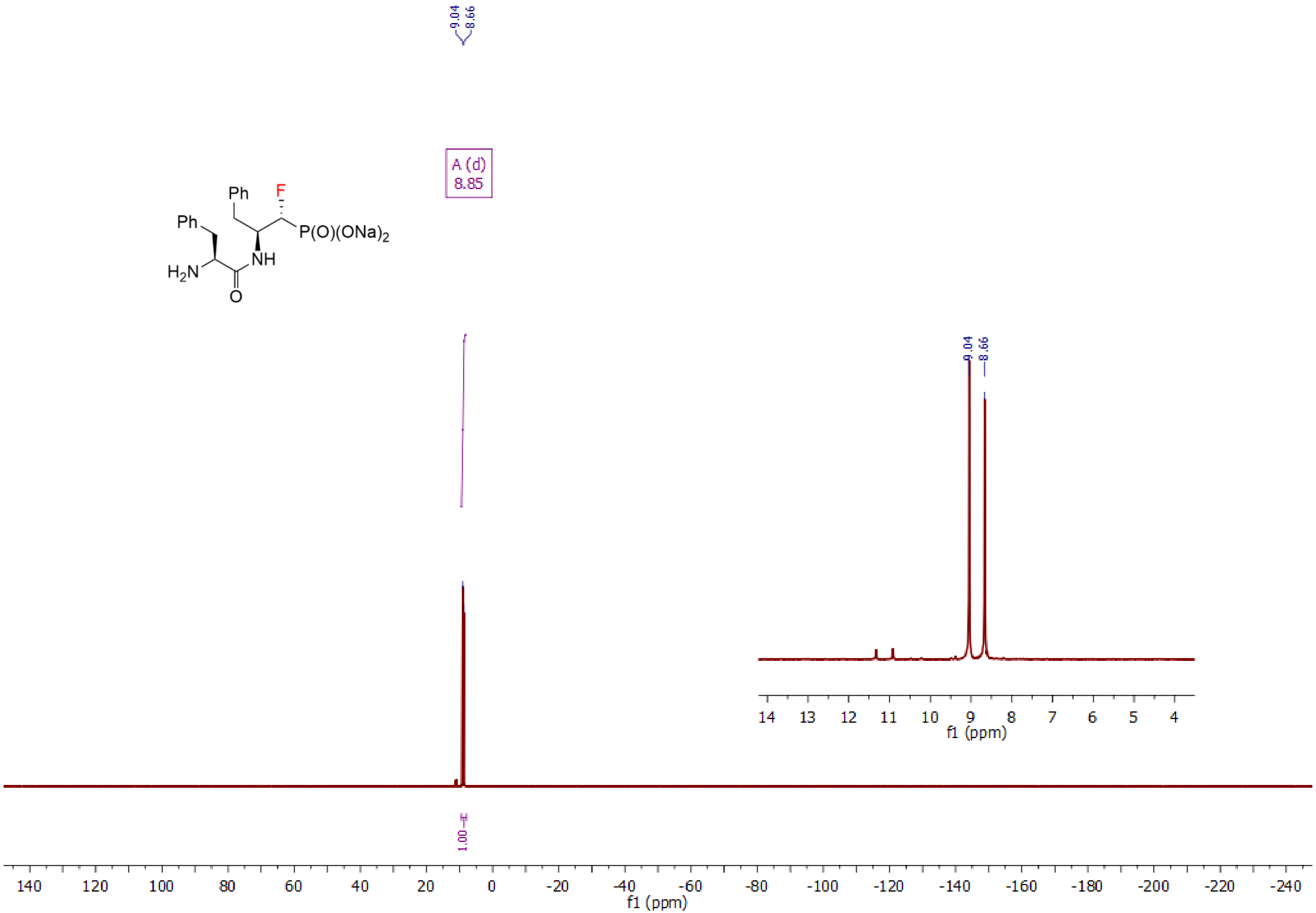

$^{31}P\{^1H\}$ NMR (162 MHz D_2O) of **9e**.

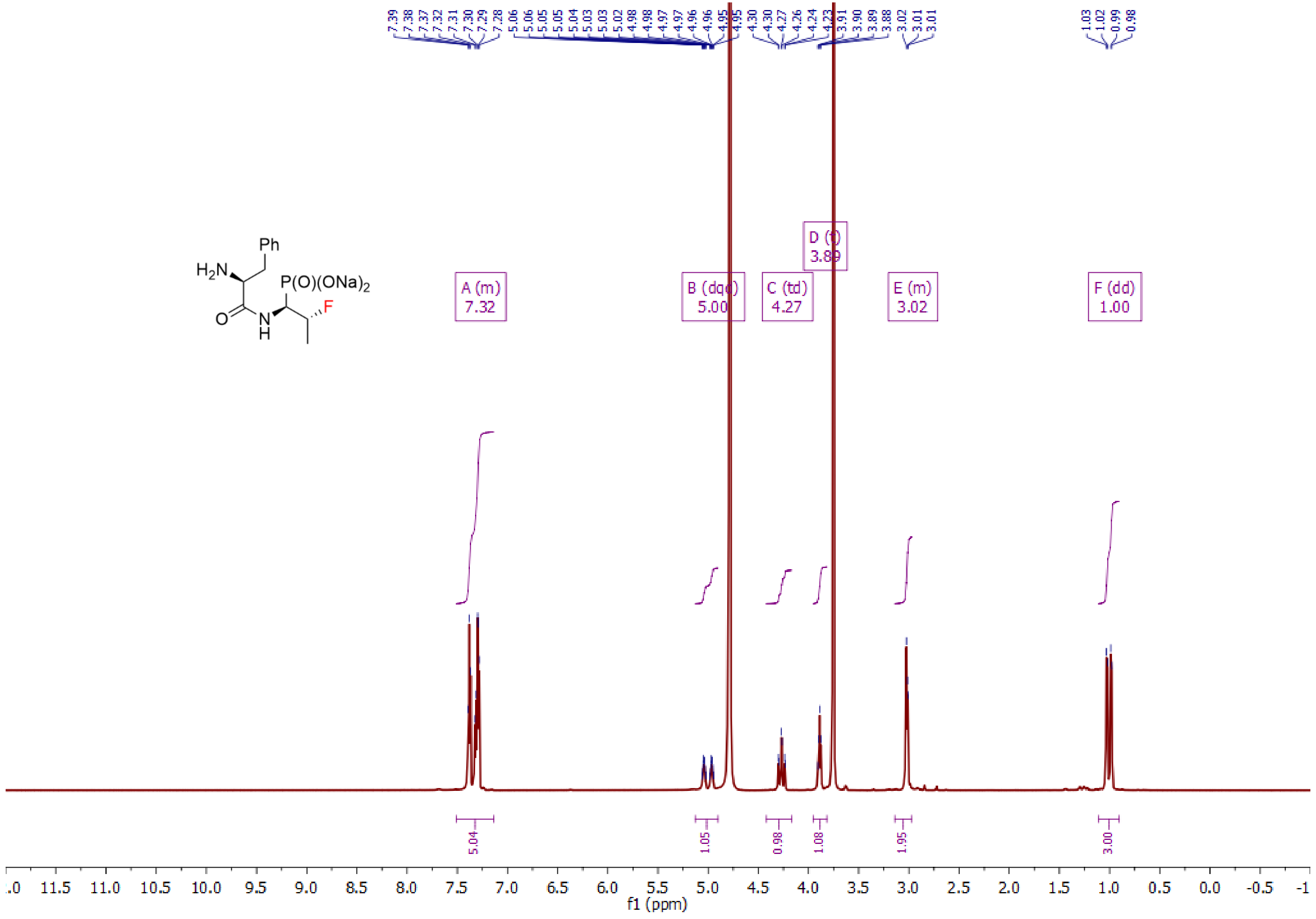

^{1}H NMR (600 MHz, D_2O) of **11a**.

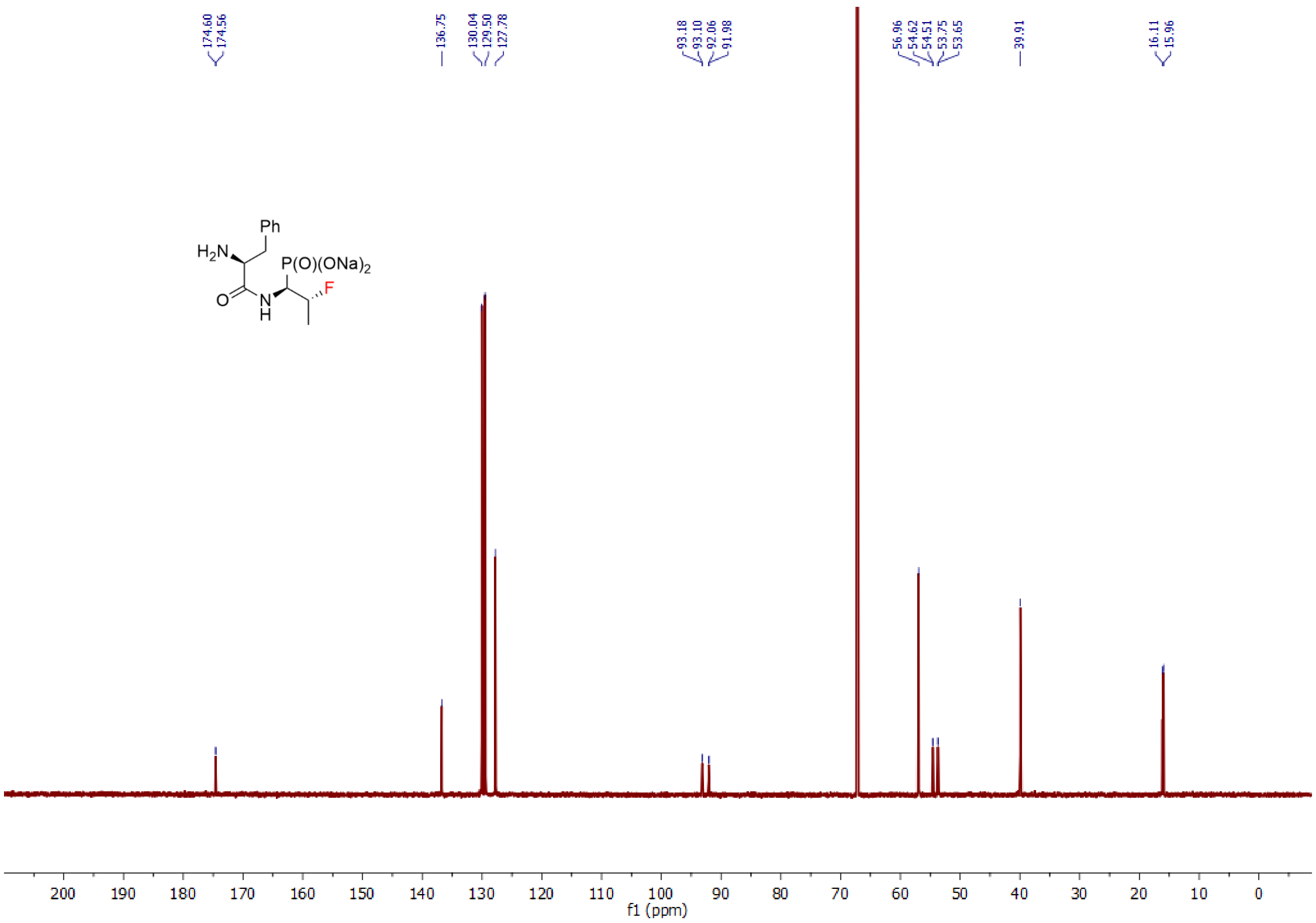

^{13}C NMR (151 MHz, D_2O) of **11a**.

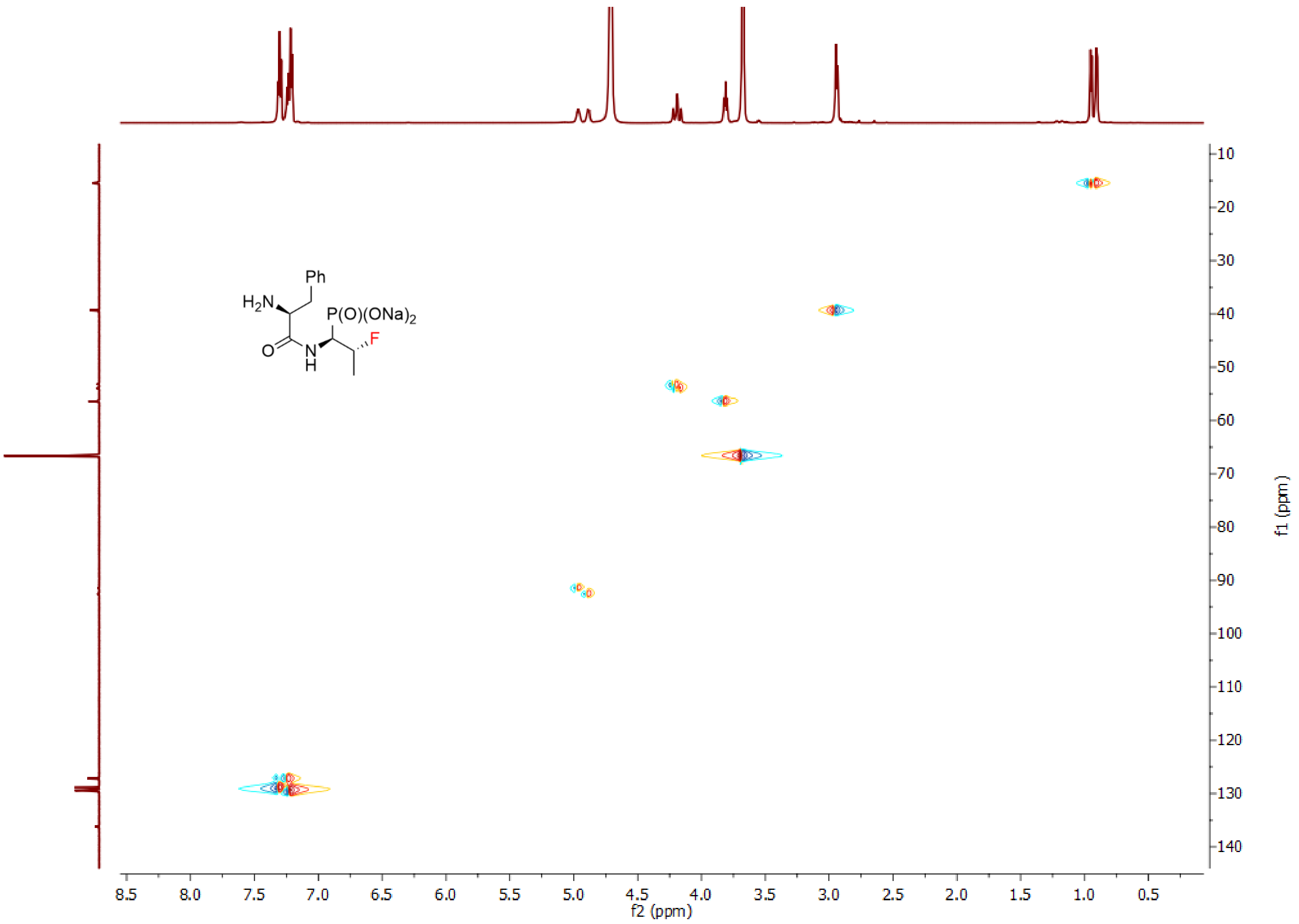

^{1}H-^{13}C HSQC (600 MHz, D_2O) of **11a**.

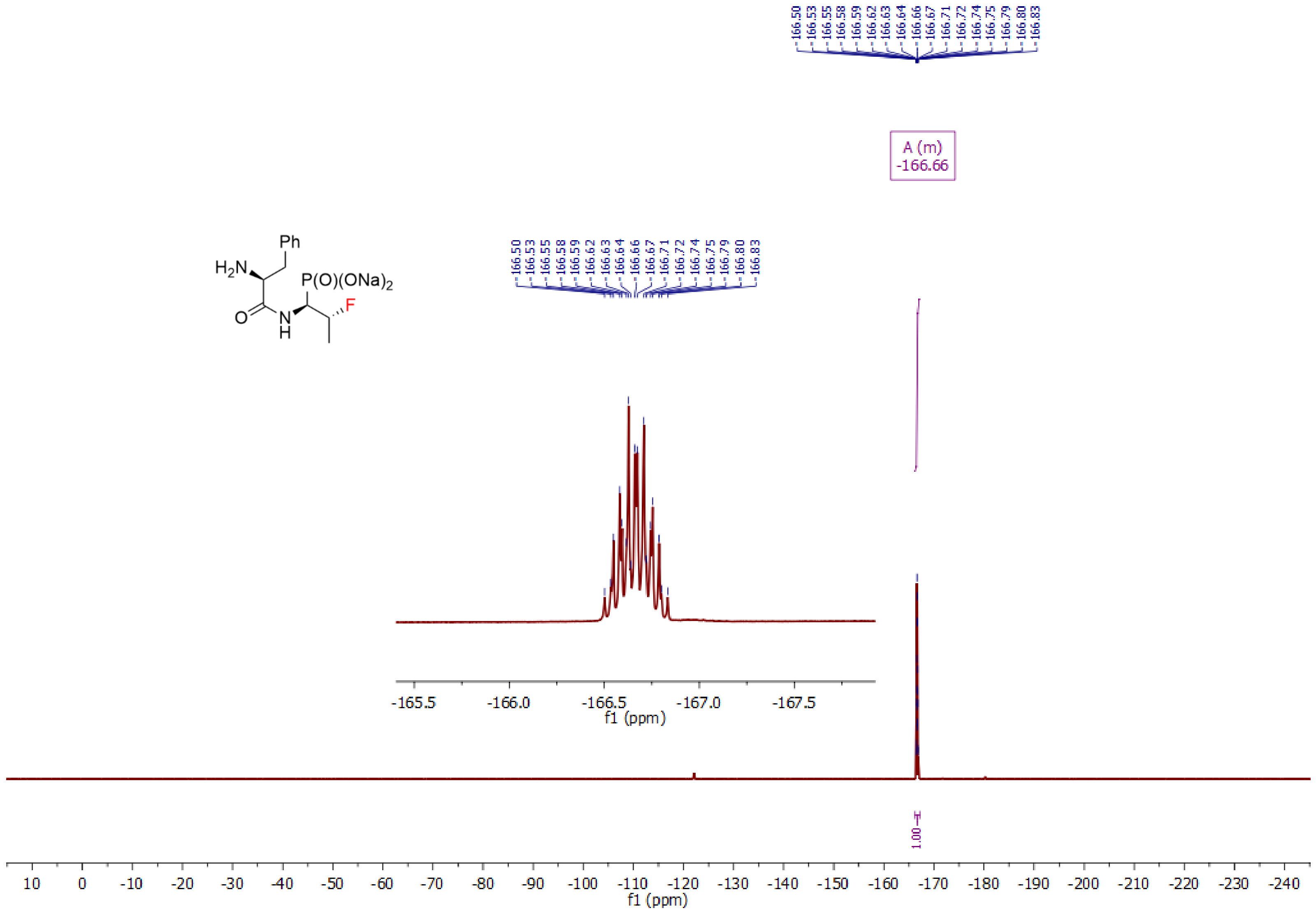

^{19}F NMR (565 MHz, D2O) of **11a**.

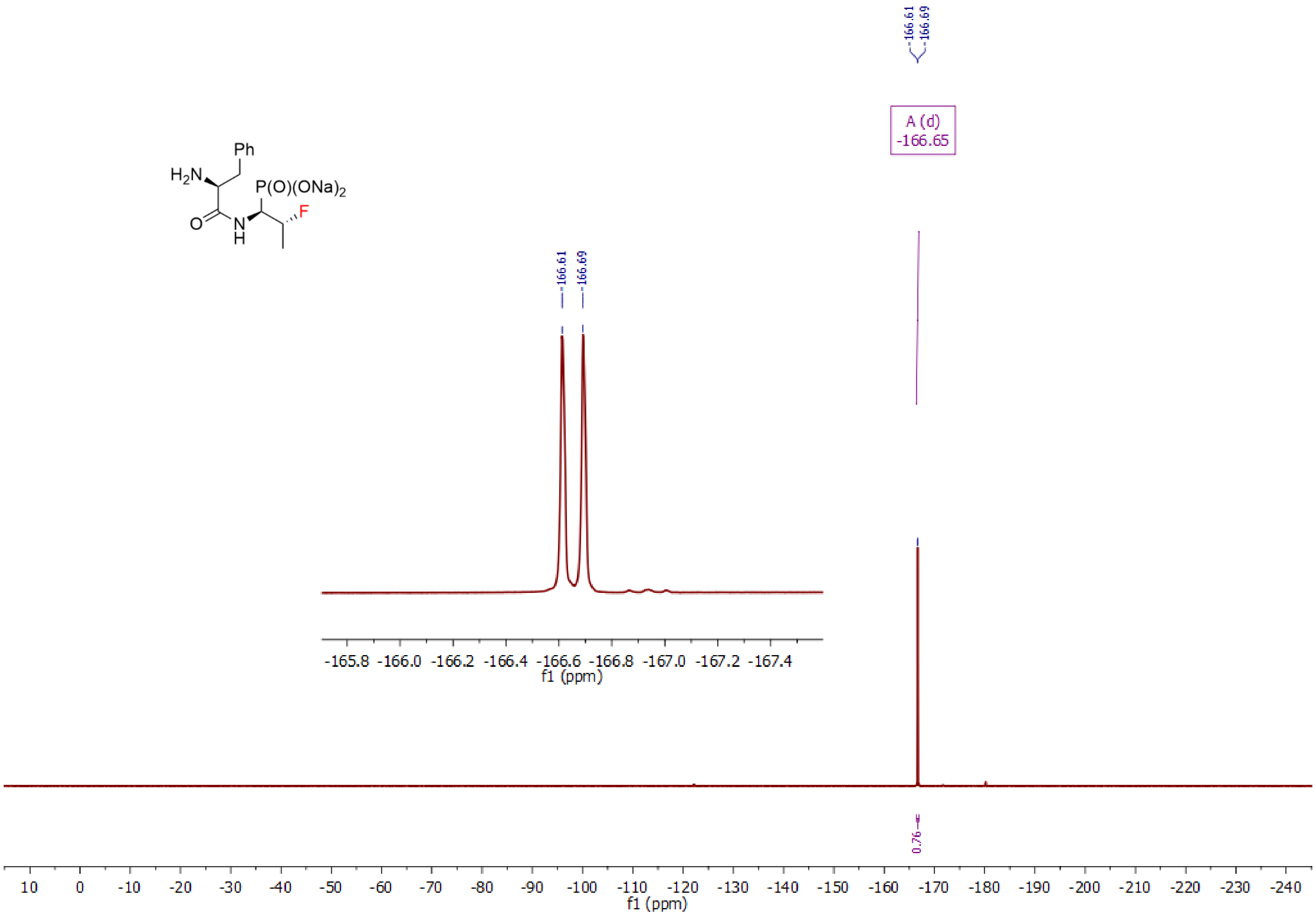

$^{19}F\{/^{1}H\}$ NMR (565 MHz, D_2O) of **11a**.

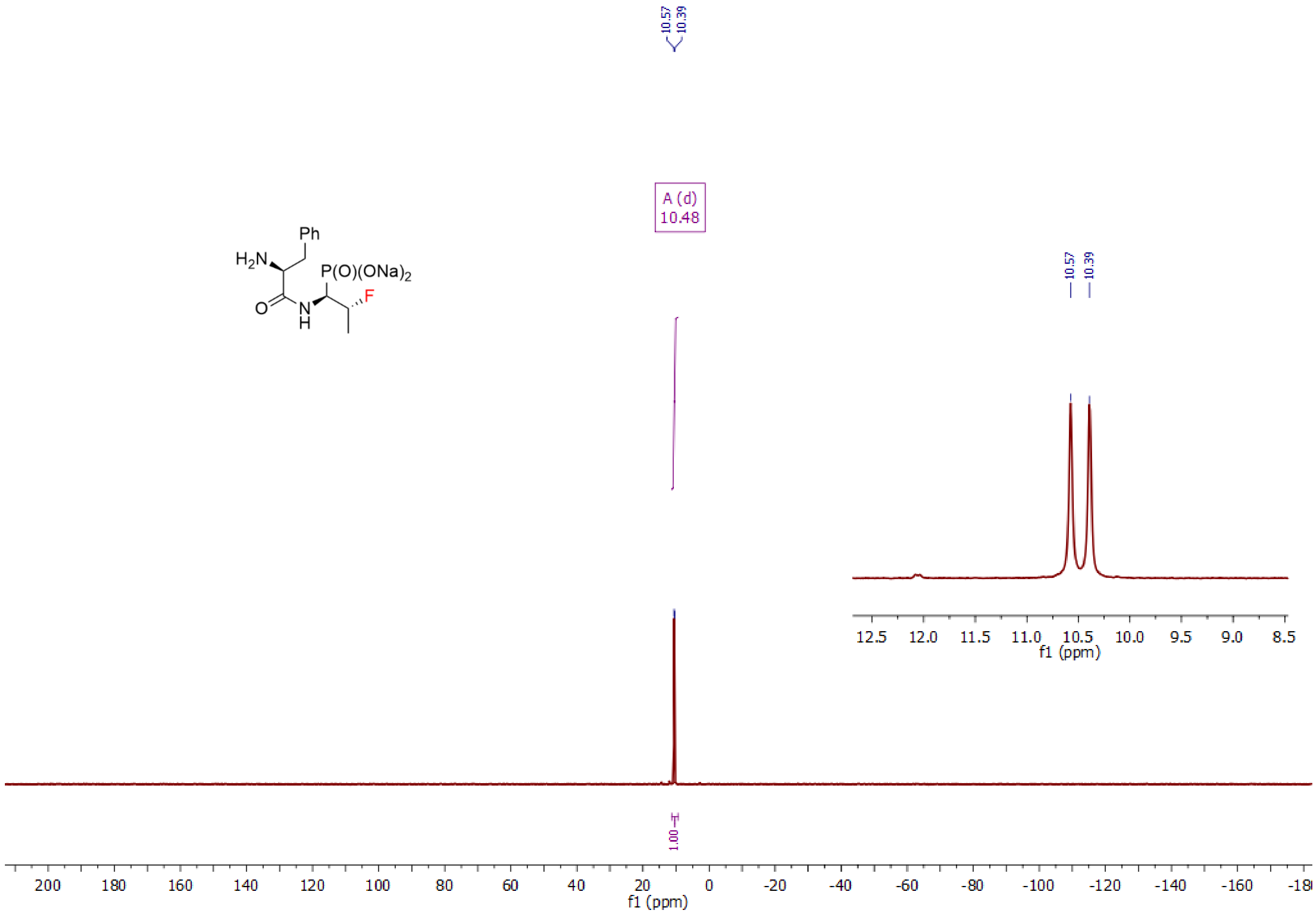

$^{31}P\{/^{1}H\}$ NMR (243 MHz, D_2O) of **11a**.

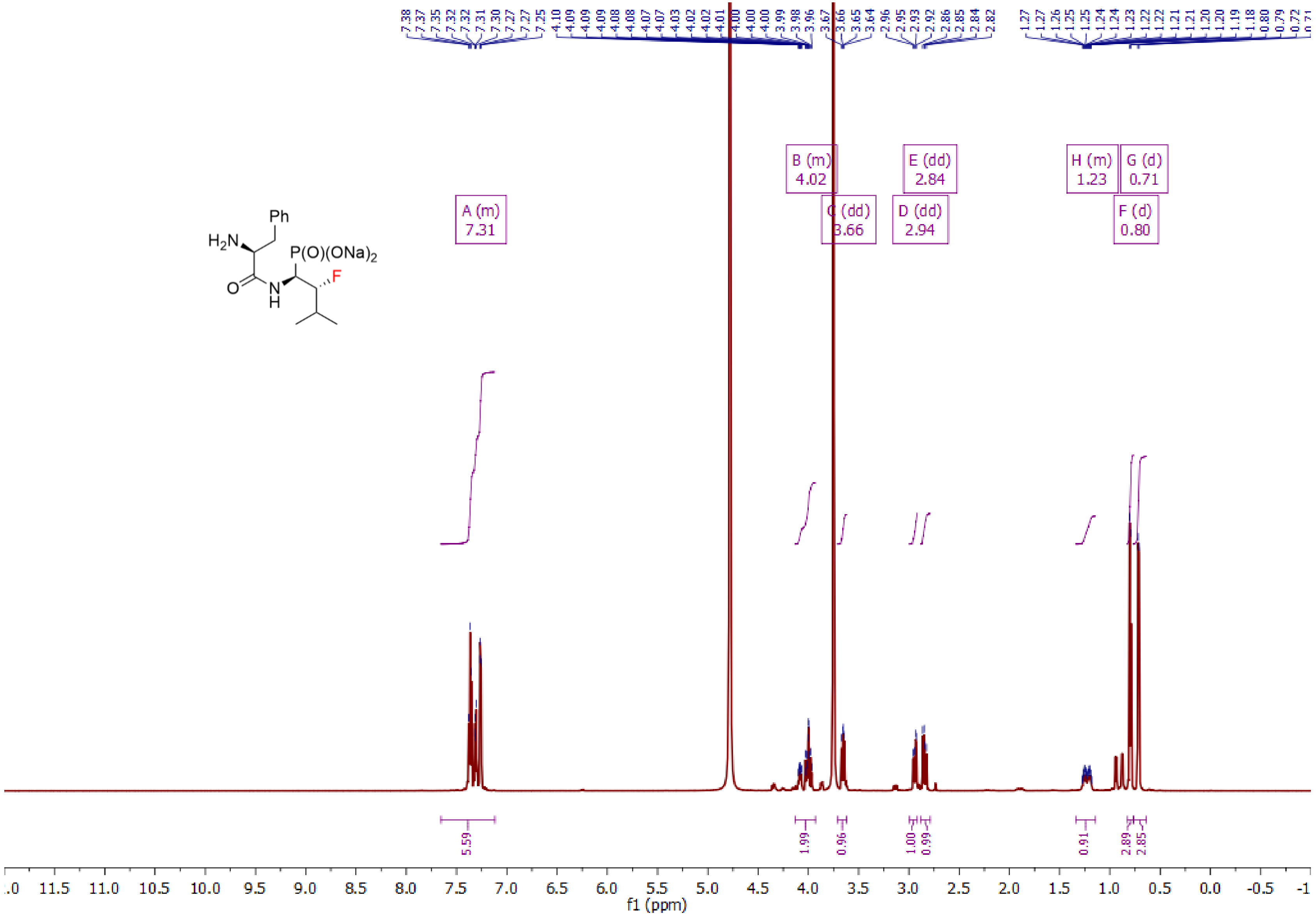

^{1}H NMR (600 MHz, D_2O) of **11b**.

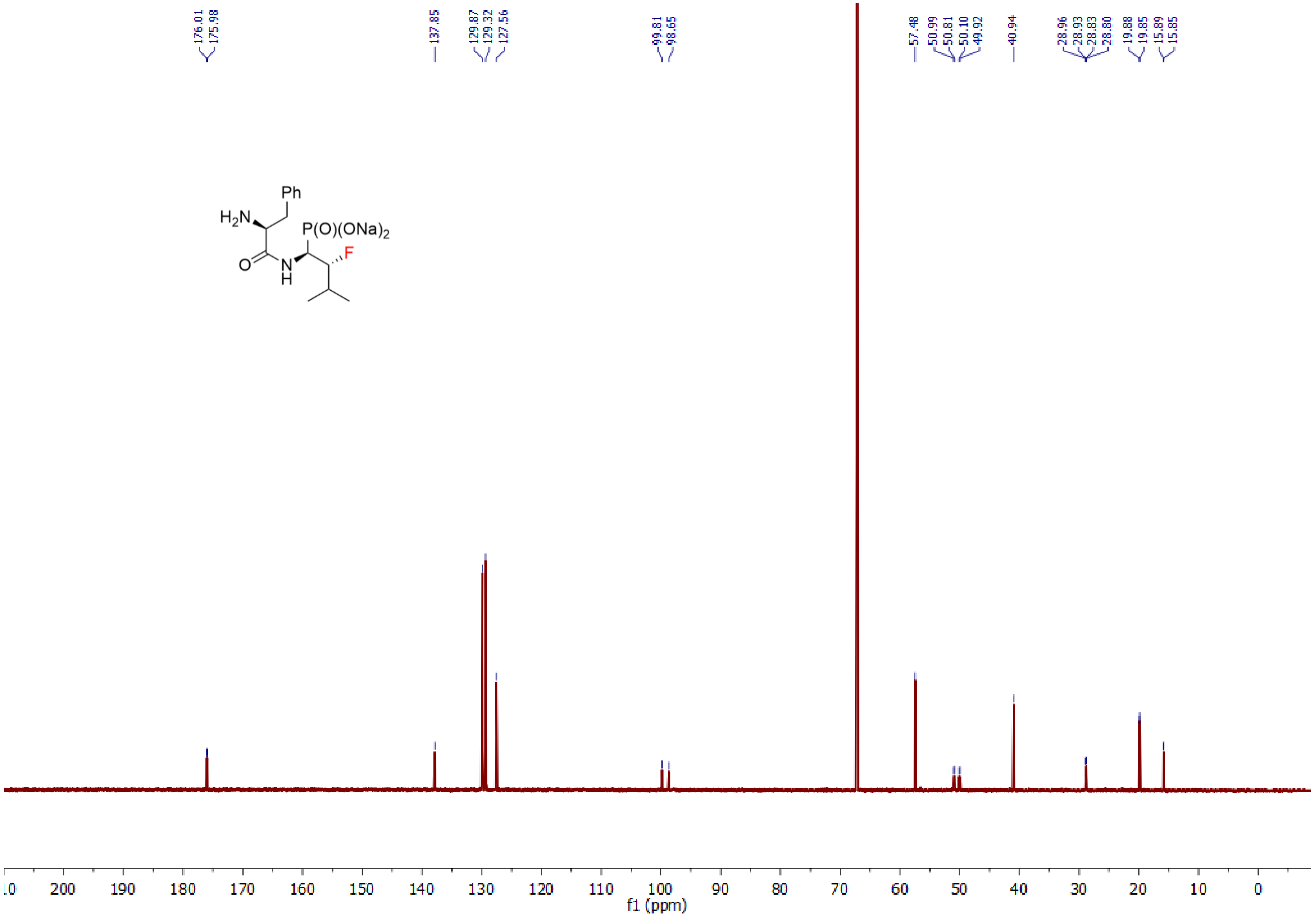

^{13}C NMR (151 MHz, D_2O) of **11b**.

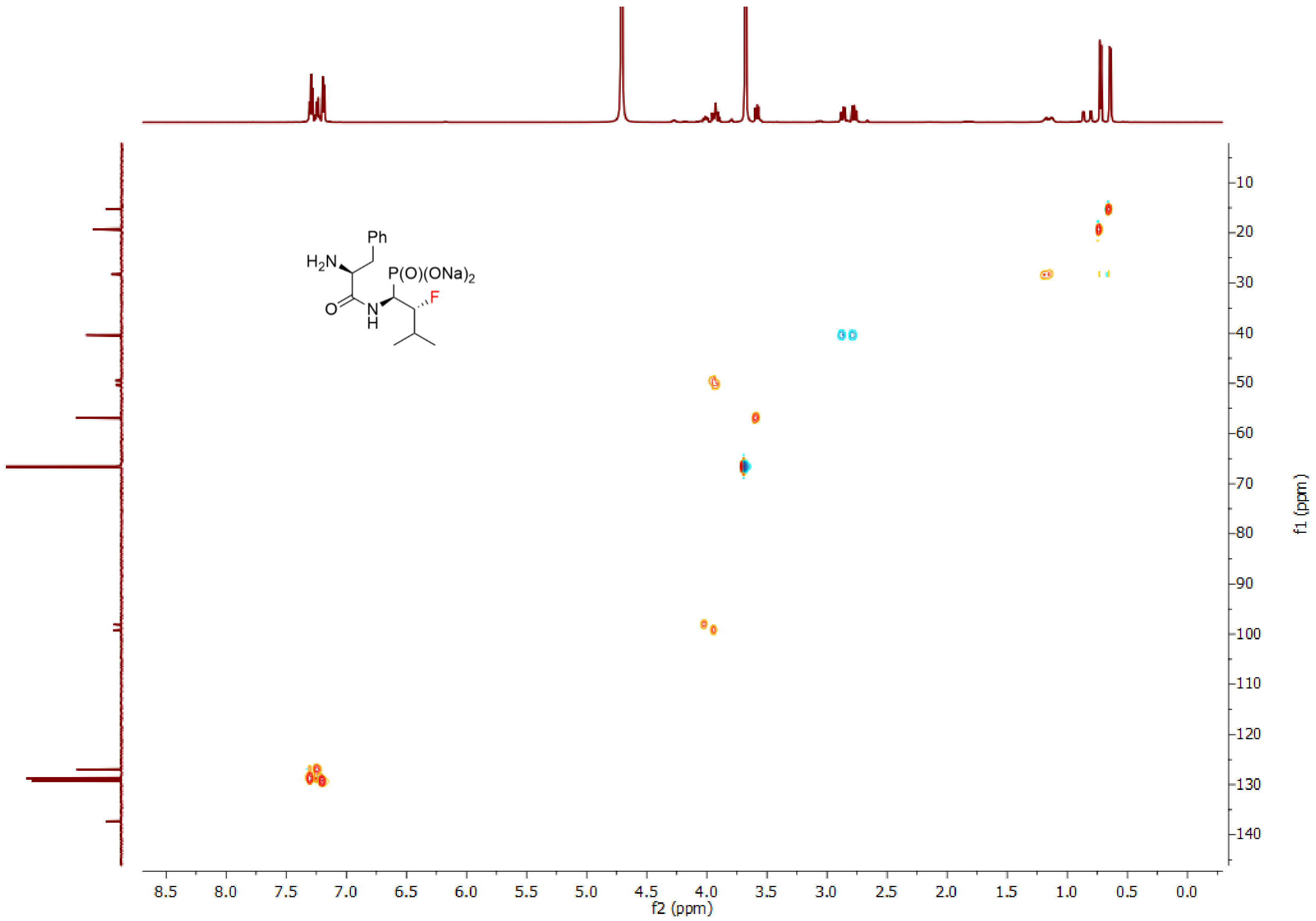

^{1}H-^{13}C HSQC (600 MHz, D_2O) of **11b**.

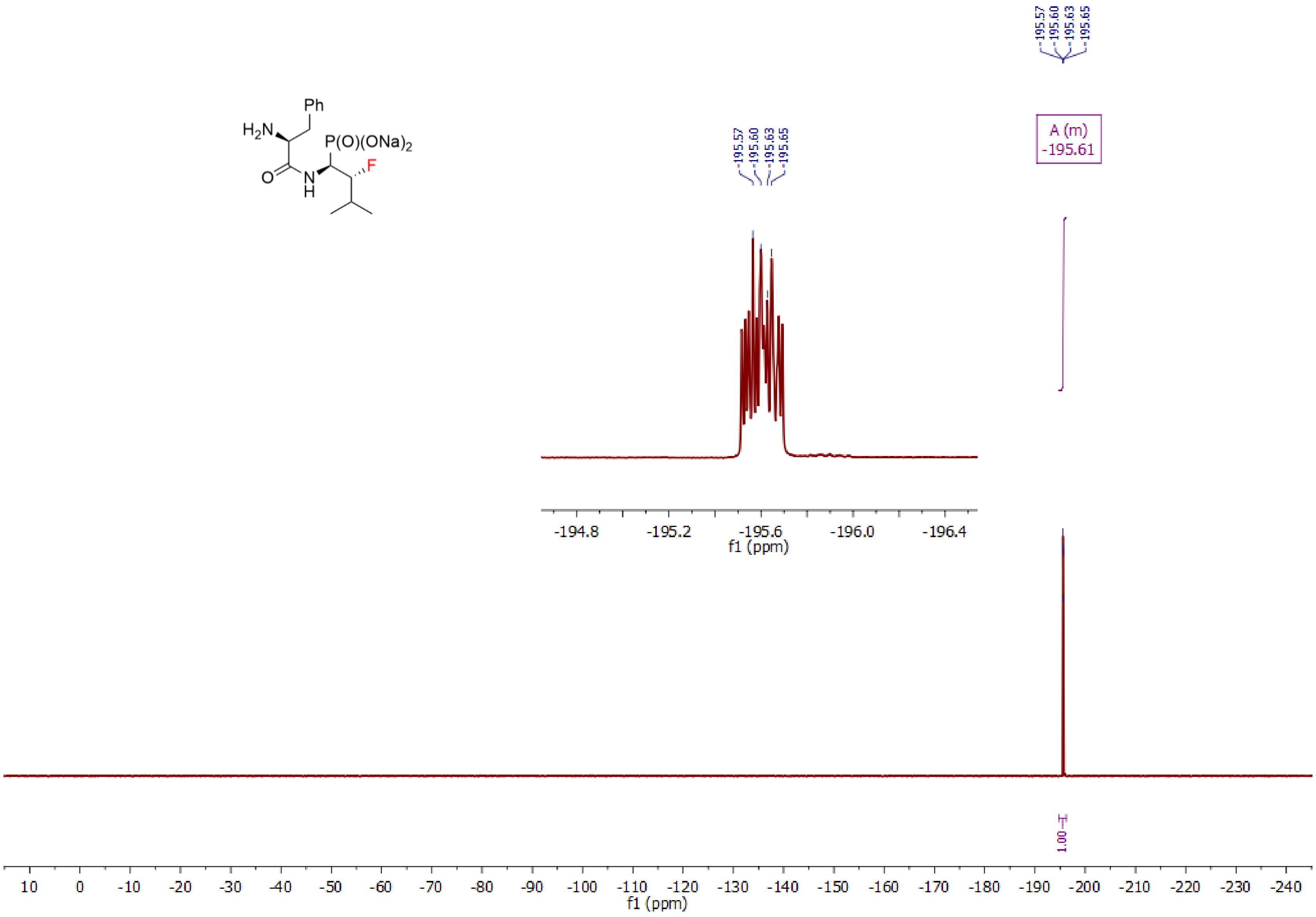

^{19}F NMR (565 MHz, D_2O) of **11b**.

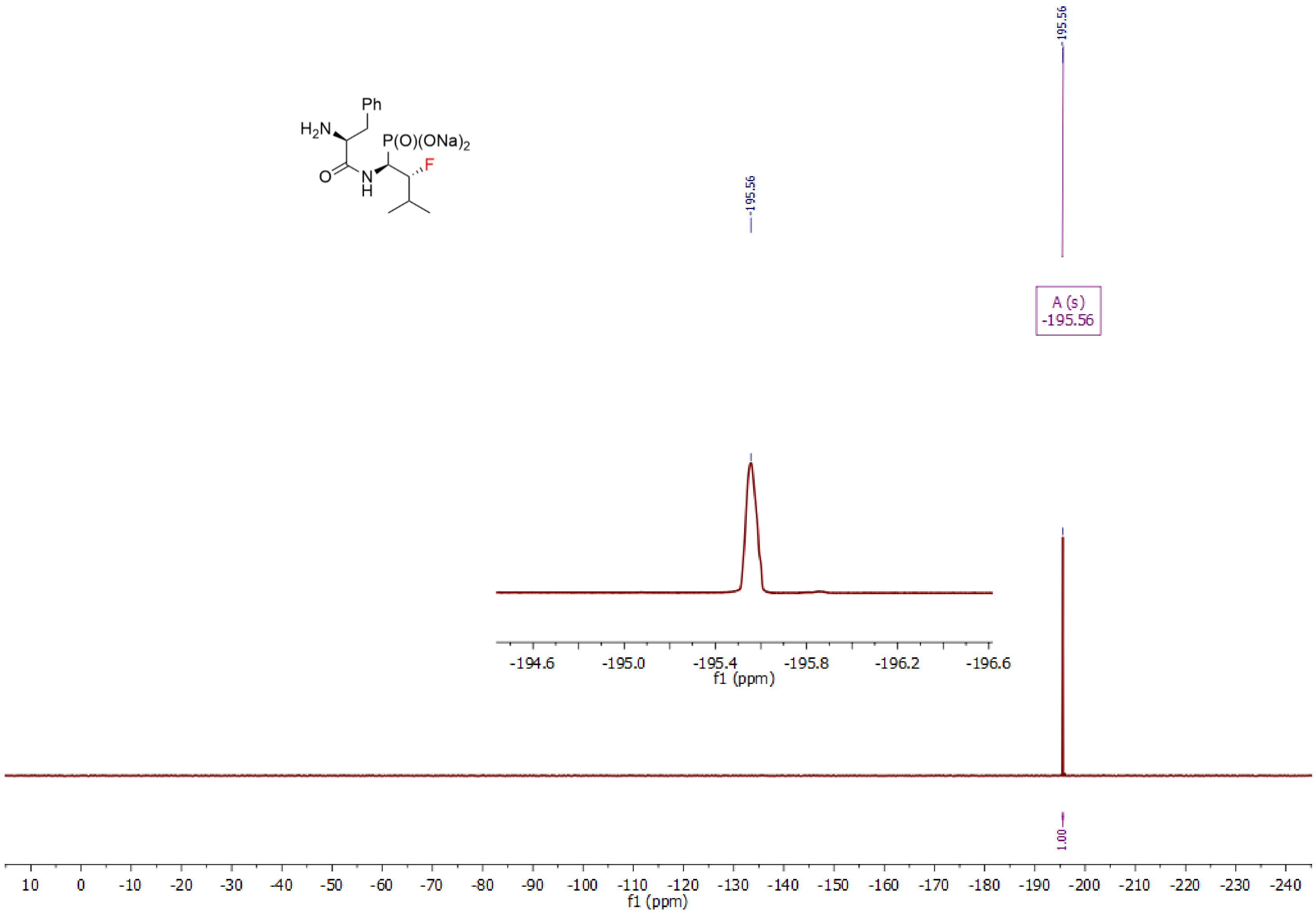

$^{19}F\{/^{1}H\}$ NMR (565 MHz, D_2O) of **11b**.

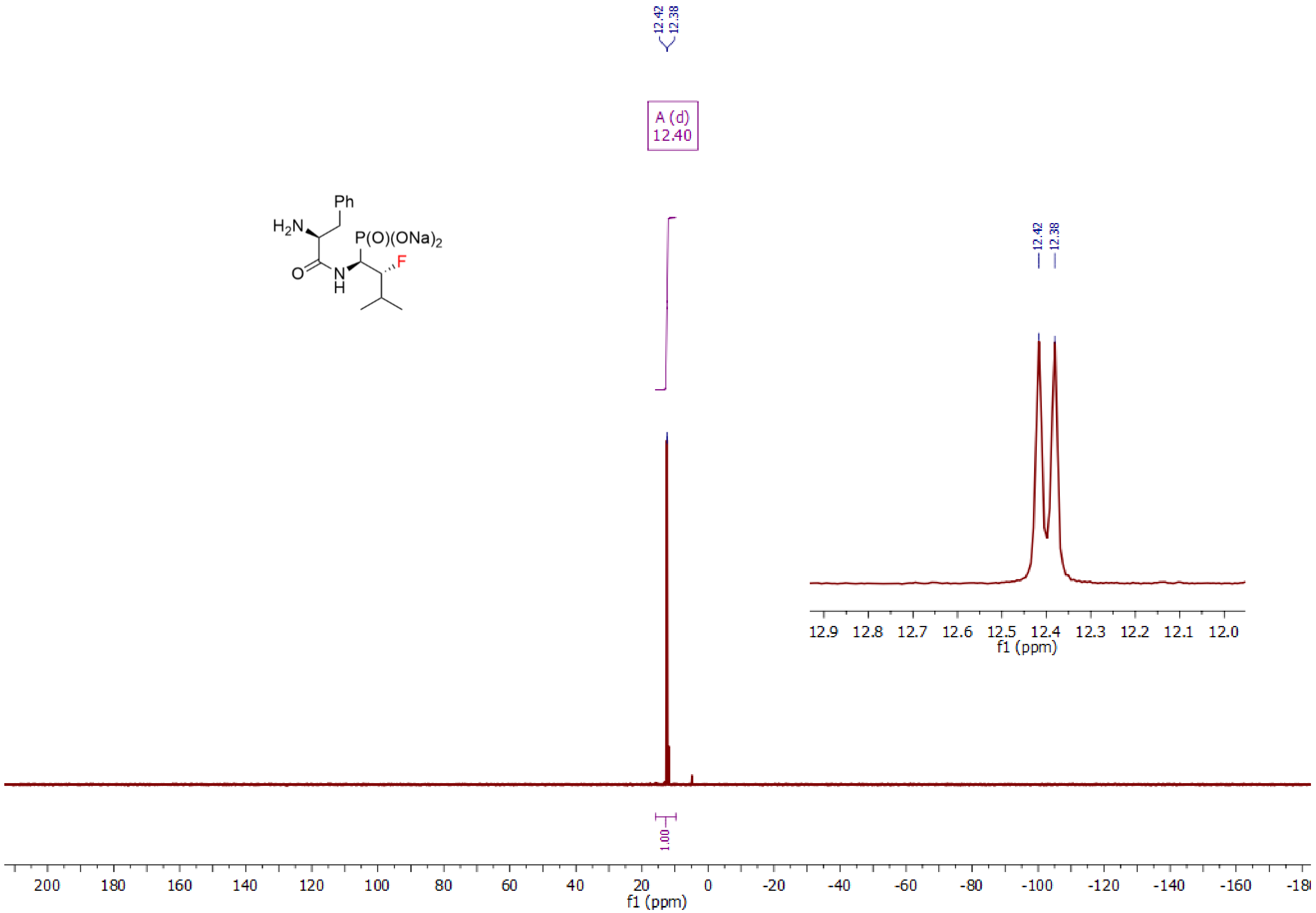

$^{31}P\{/^{1}H\}$ NMR (243 MHz, D_2O) of **11b**.

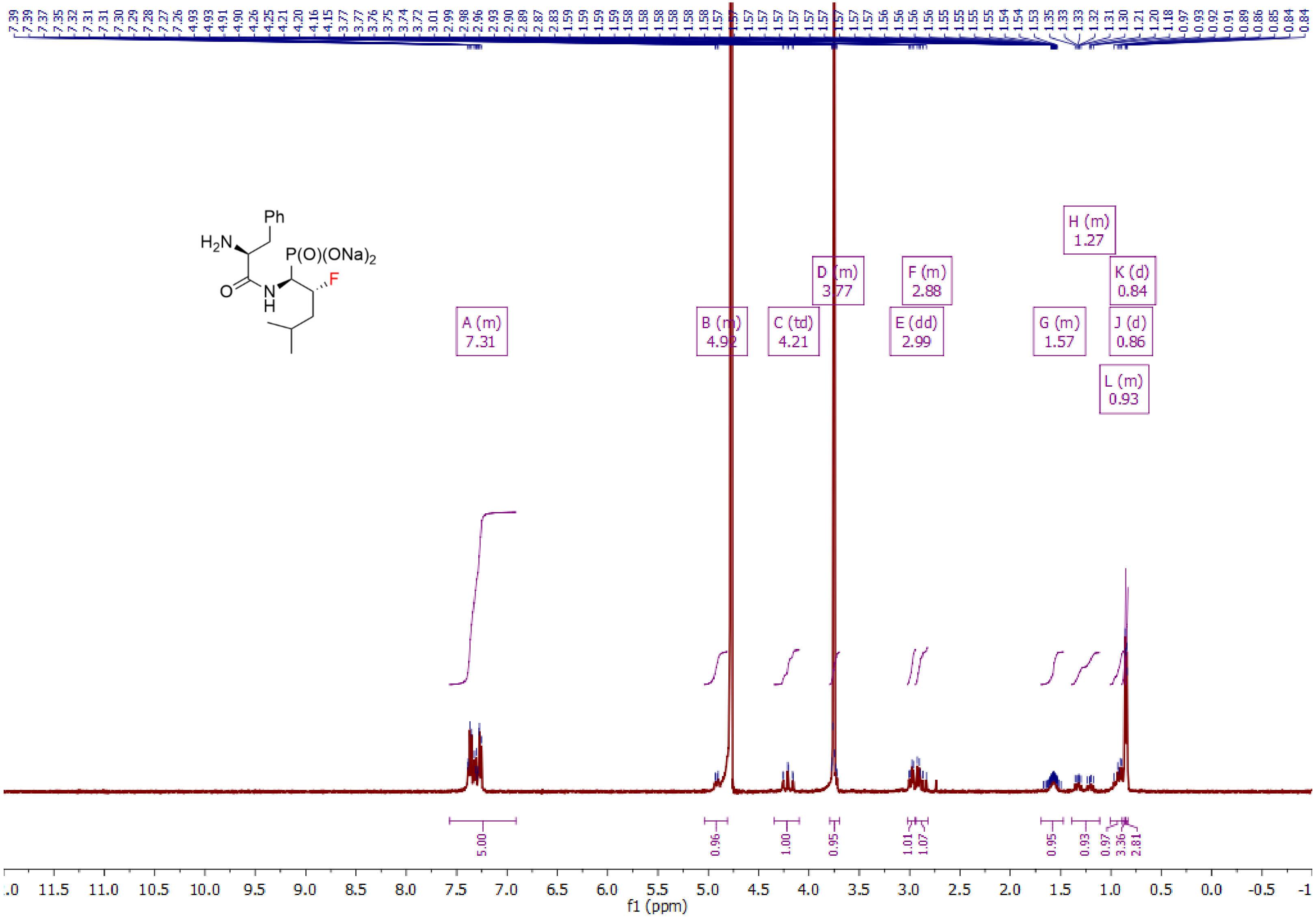

^{1}H NMR (400 MHz, D_2O) of **11c**.

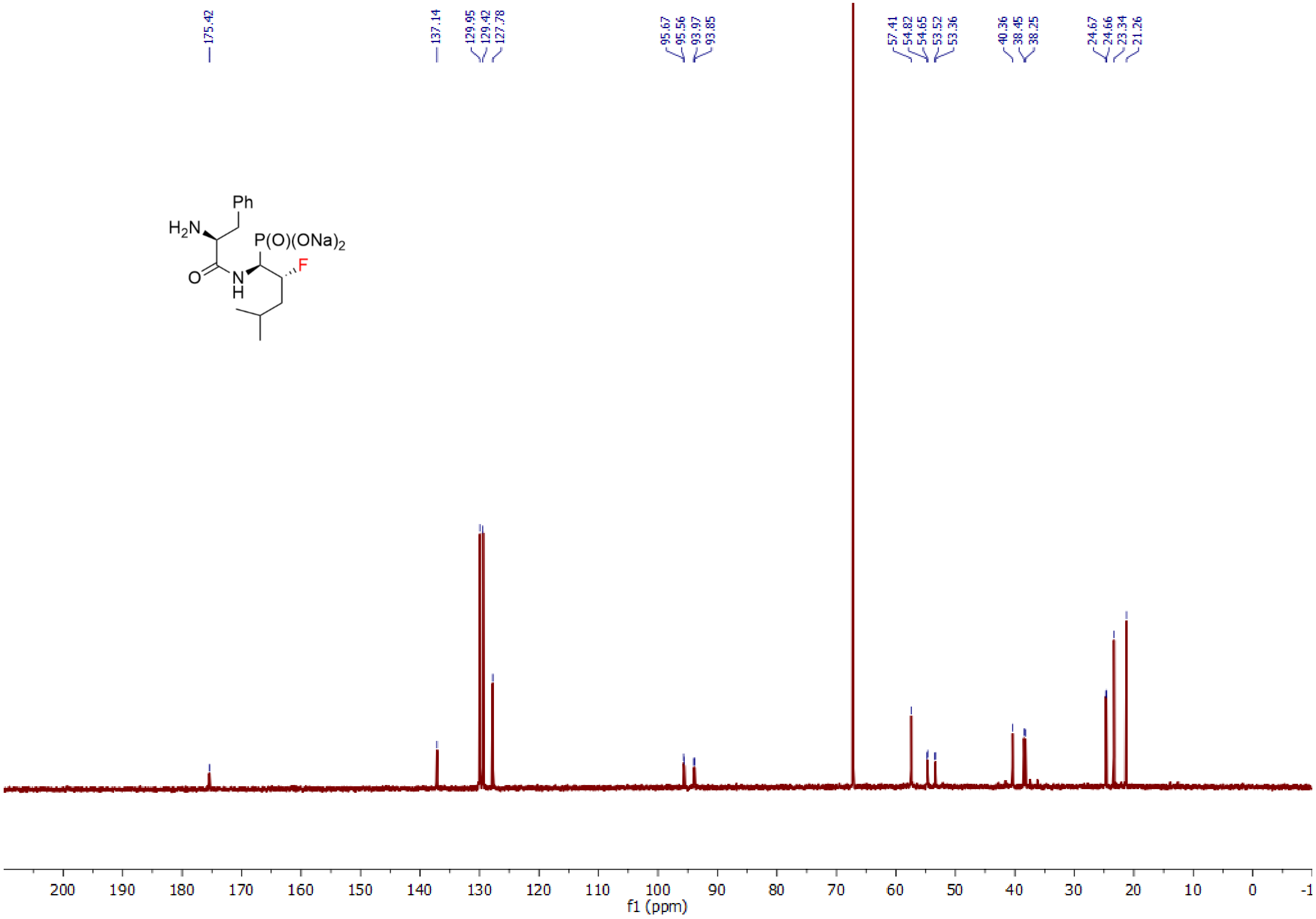

^{13}C NMR (101 MHz, D_2O) of **11c**.

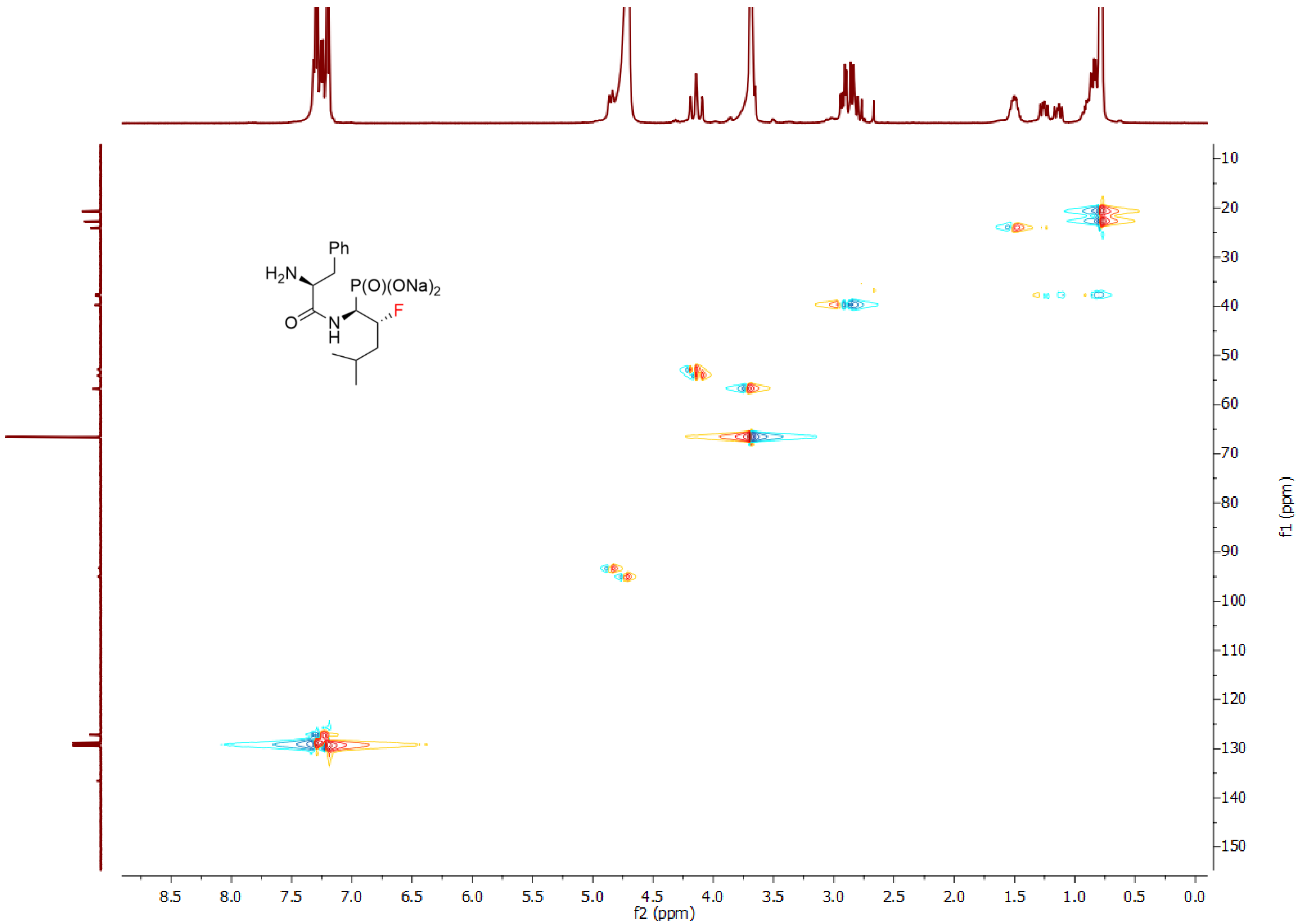

^{1}H-^{13}C HSQC (400 MHz, D_2O) of **11c**.

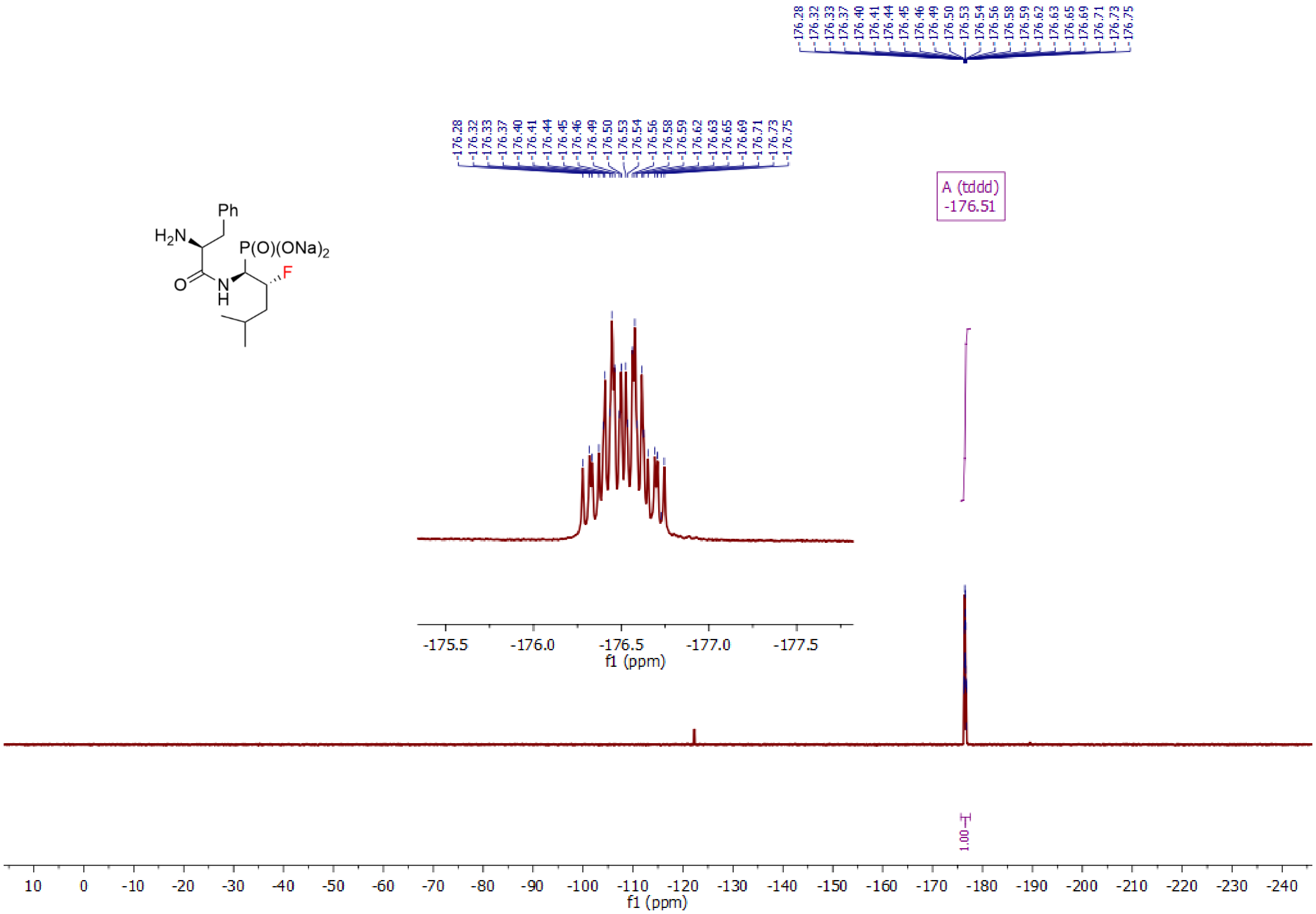

^{19}F NMR (376 MHz D_2O) of **11c**.

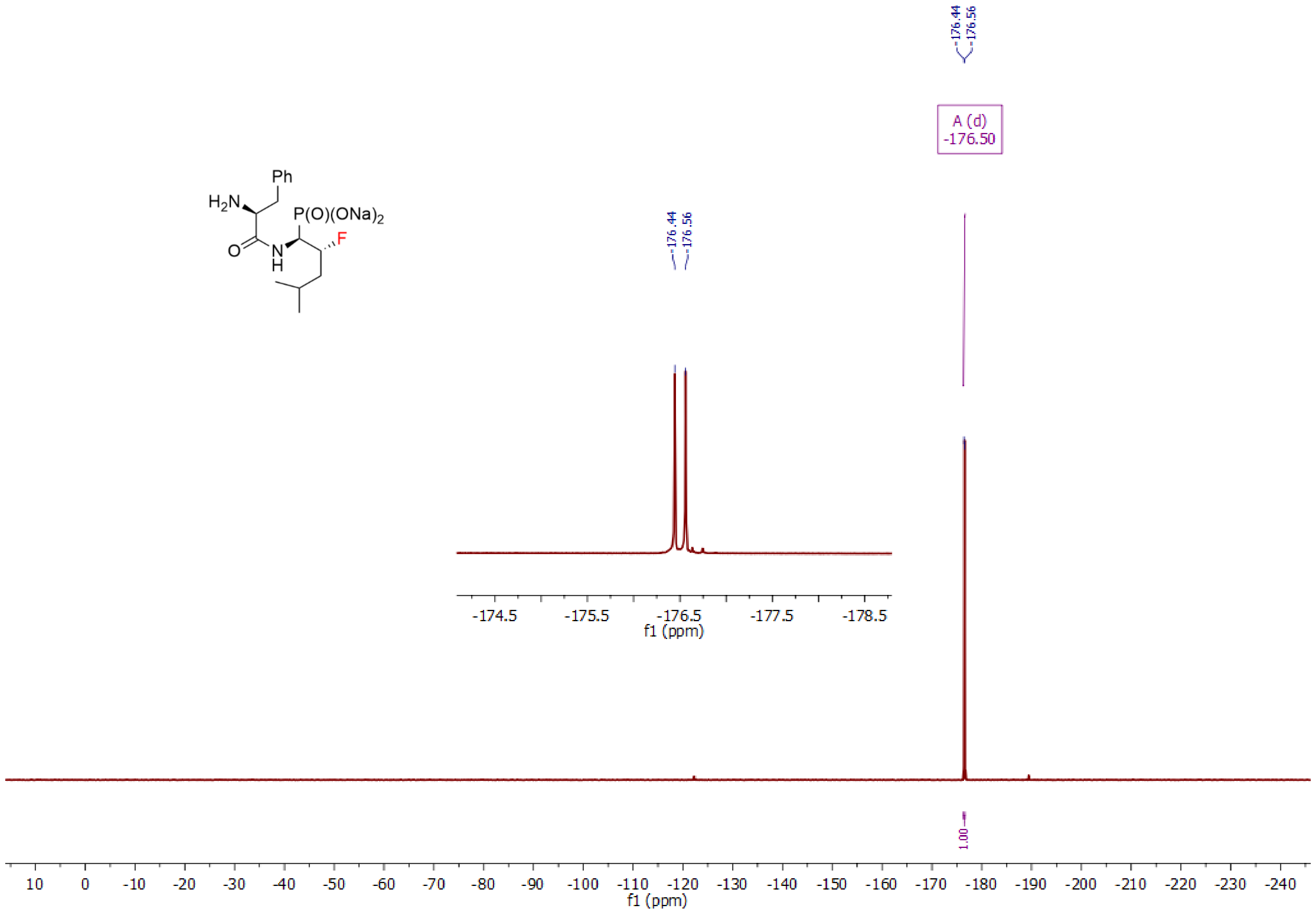

$^{19}F\{/^{1}H\}$ NMR (376 MHz D_2O) of **11c**.

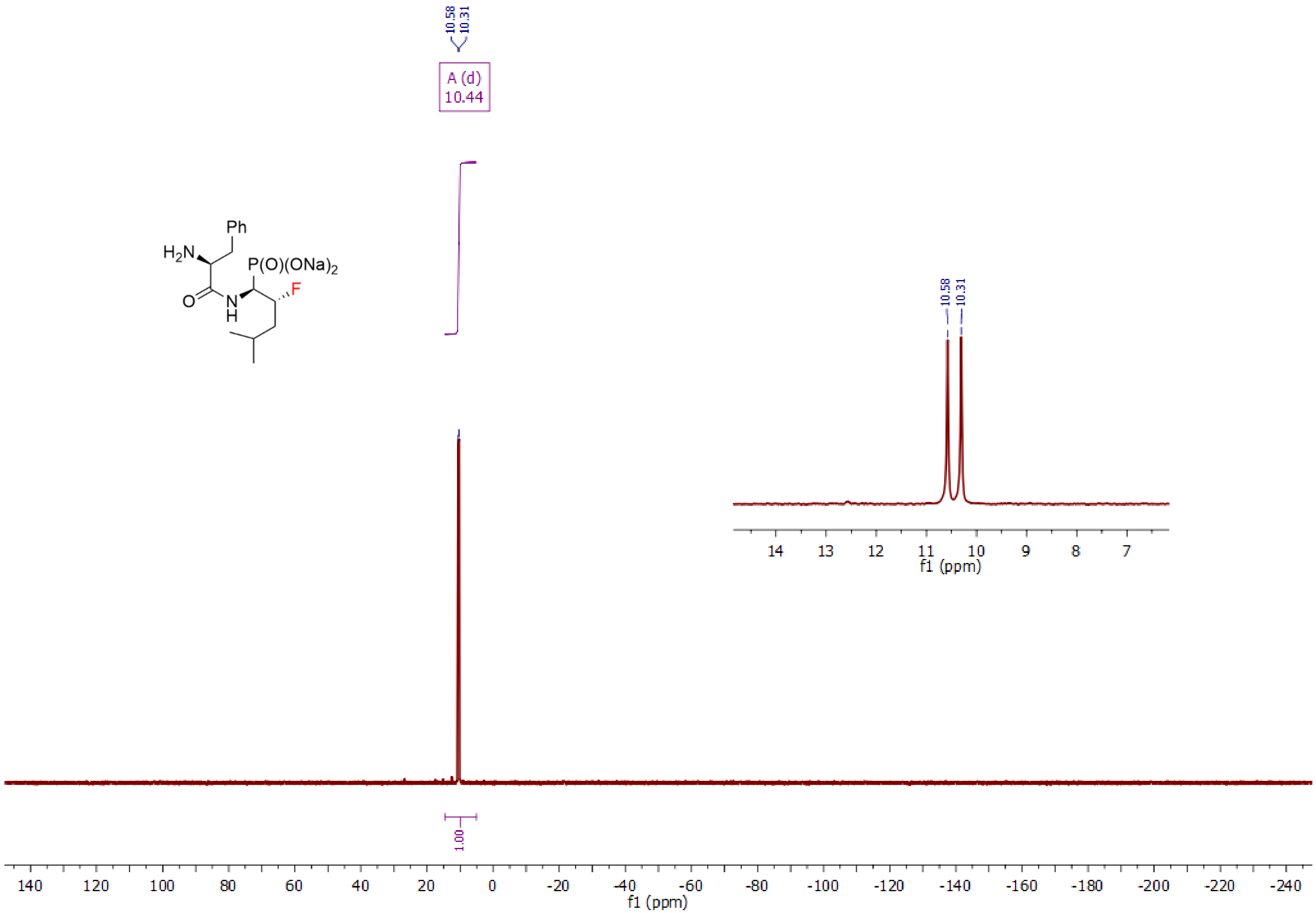

$^{31}P\{/^{1}H\}$ NMR (162 MHz D_2O) of **11c**.

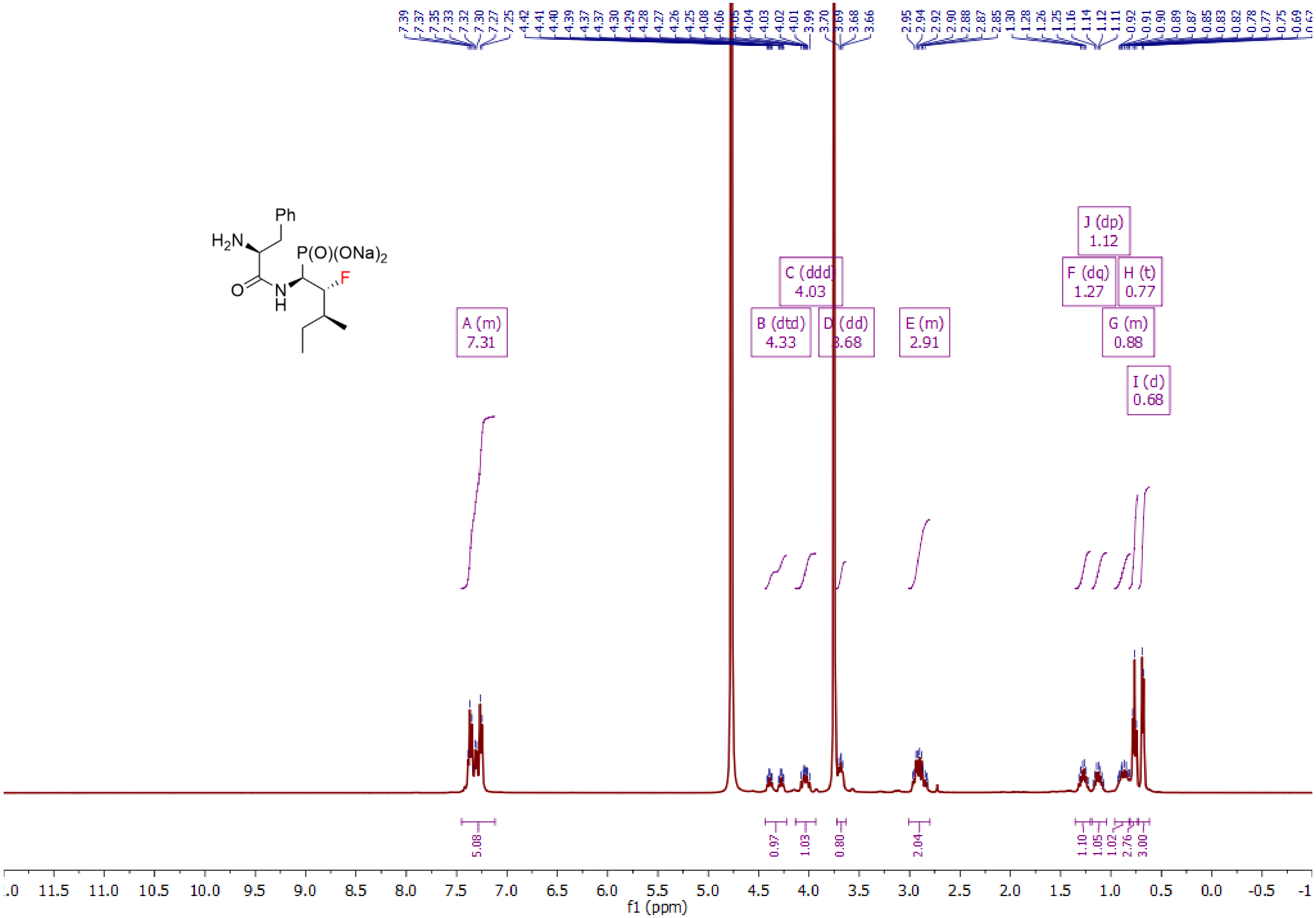

^{1}H NMR (400 MHz, D_2O) of **11d**.

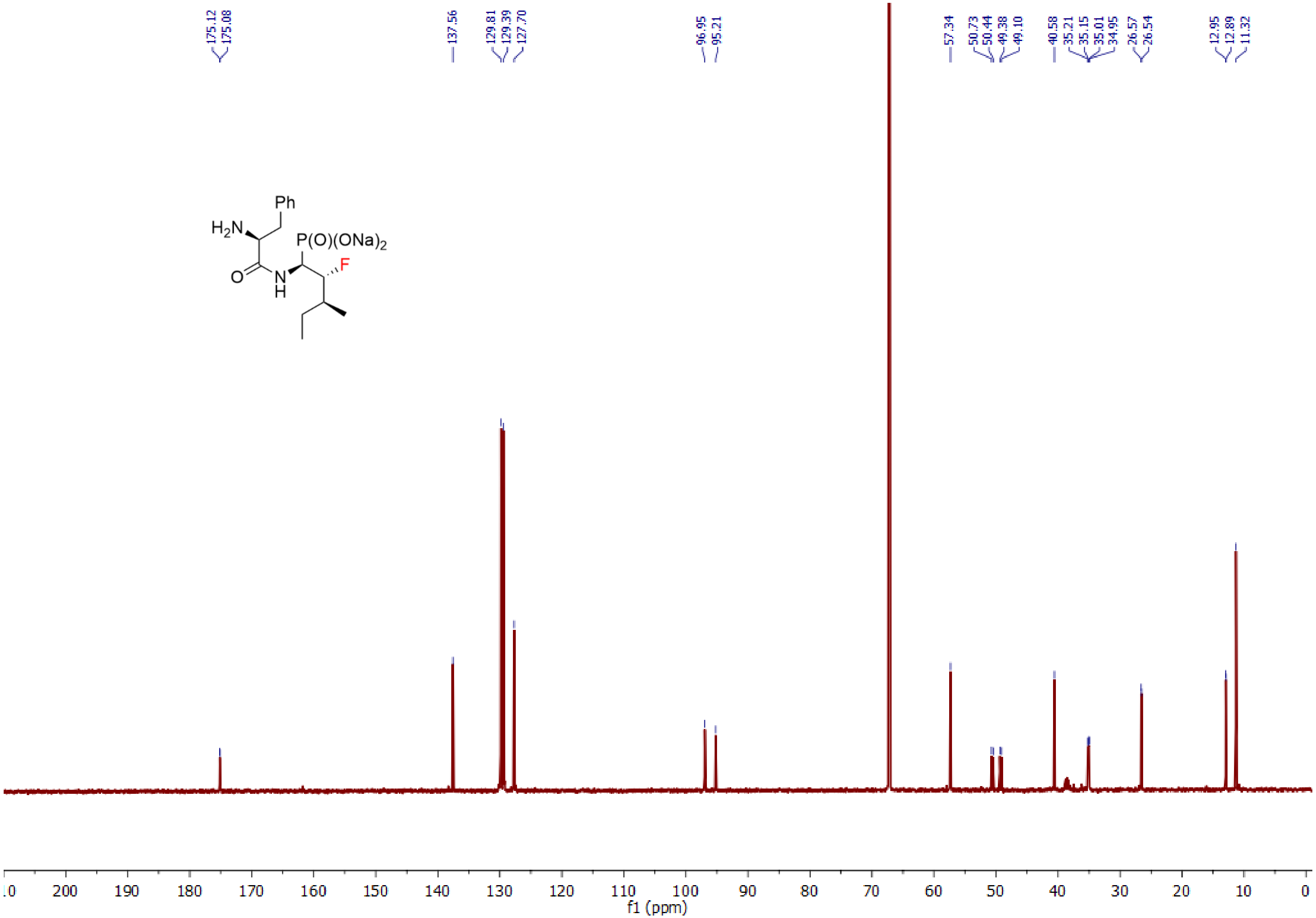

^{13}C NMR (101 MHz, D_2O) of **11d**.

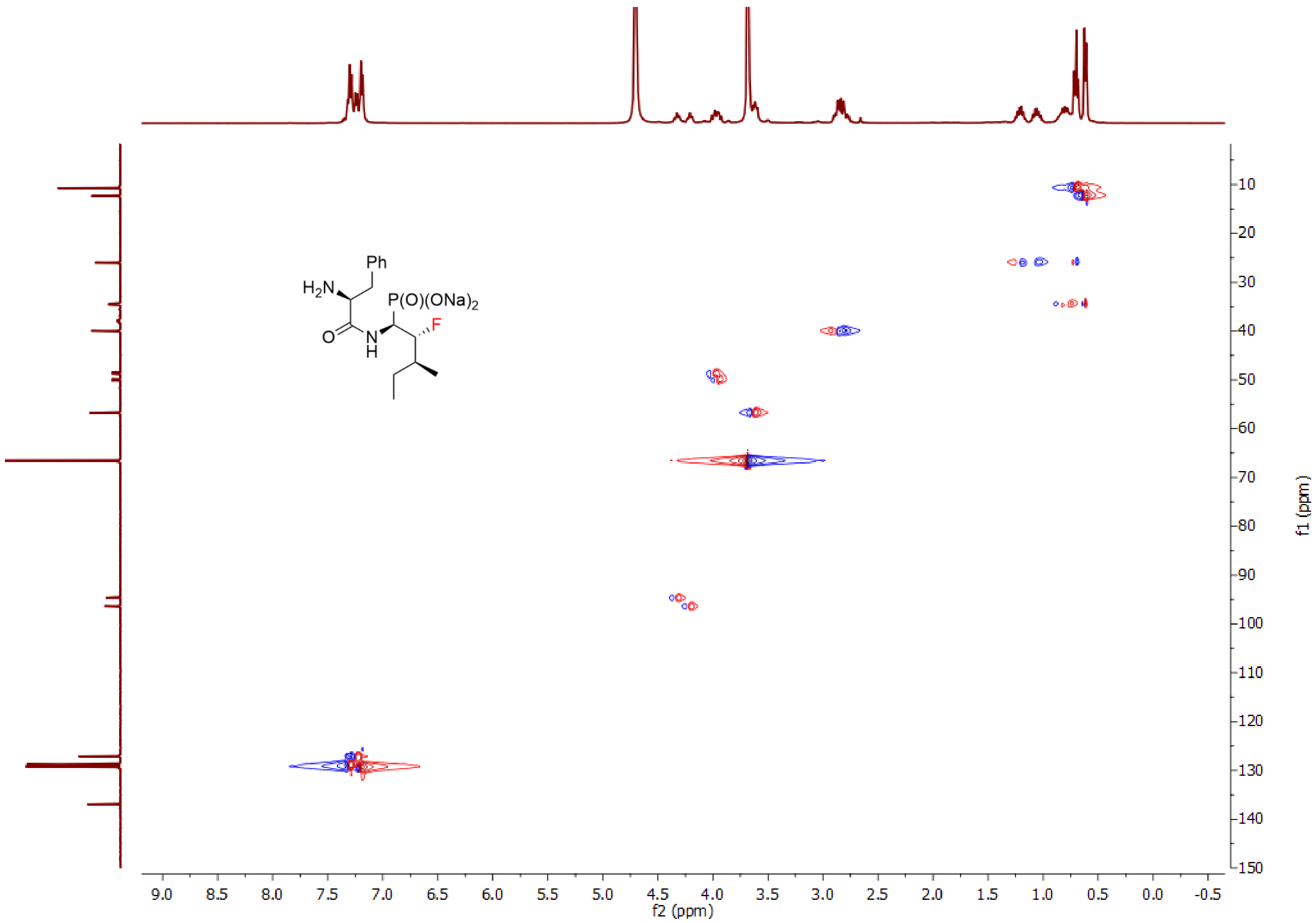

^{1}H-^{13}C HSQC (400 MHz, D_2O) of **11d**.

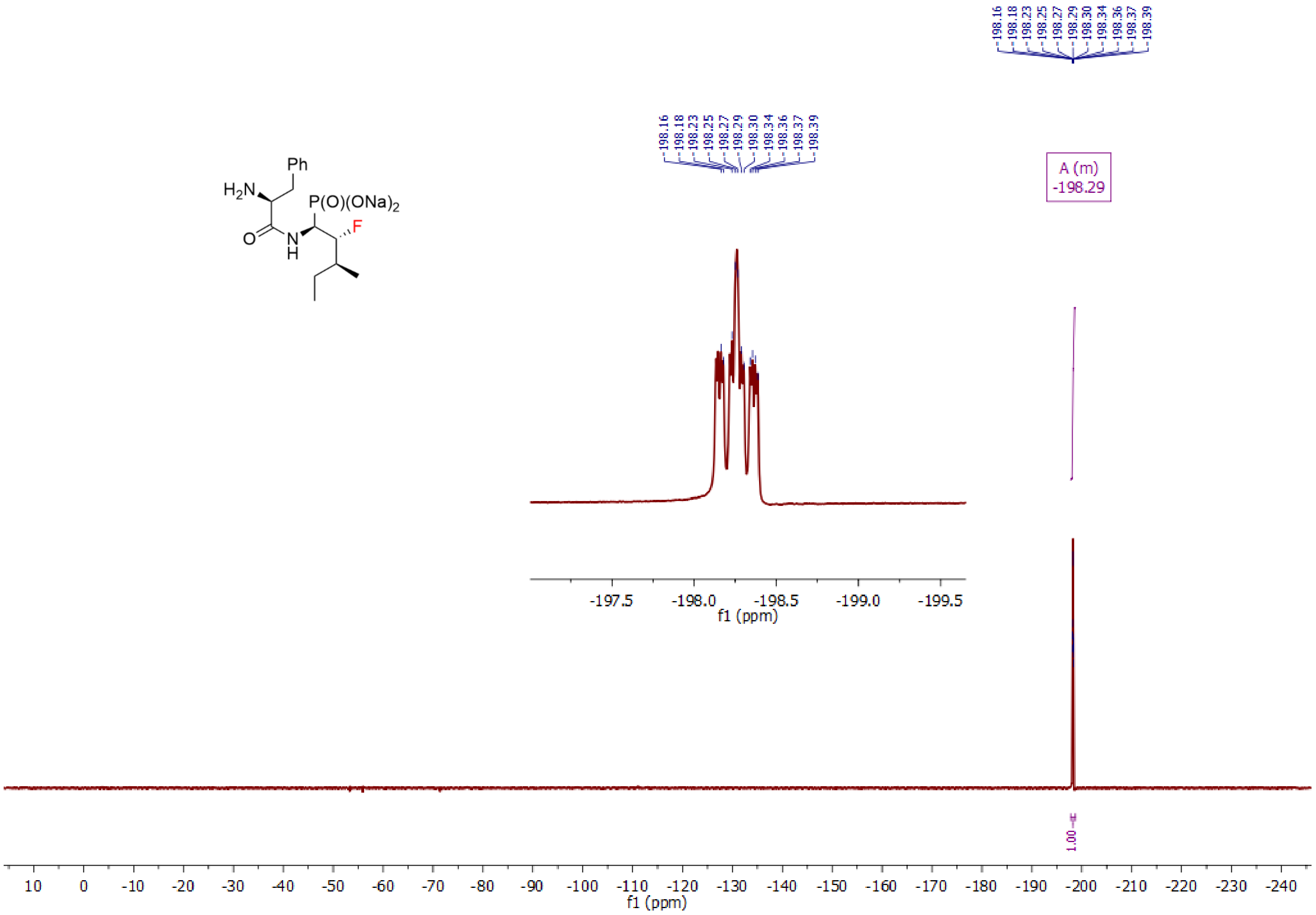

^{19}F NMR (376 MHz D_2O) of **11d**.

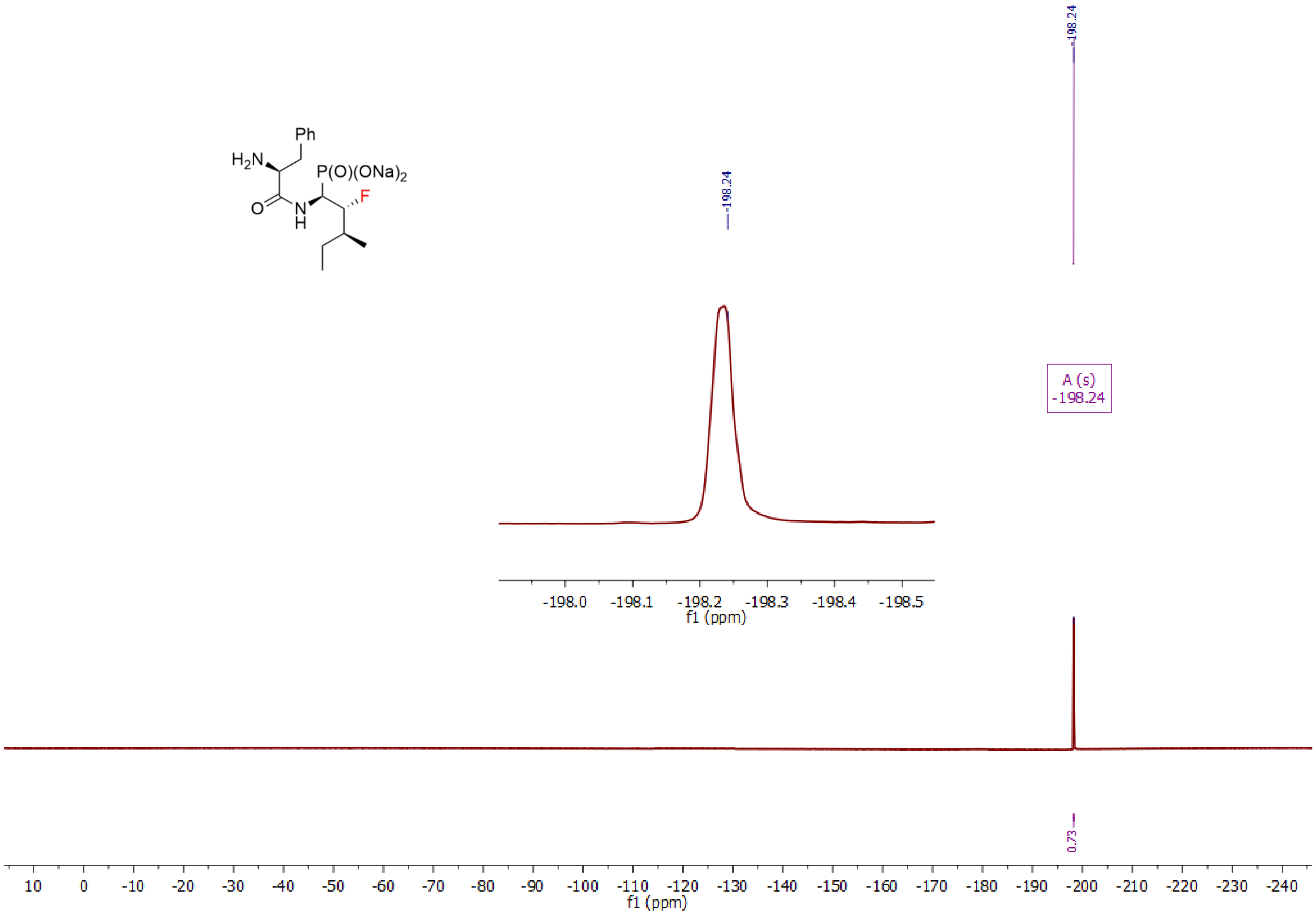

$^{19}F\{/^{1}H\}$ NMR (376 MHz D_2O) of **11d**.

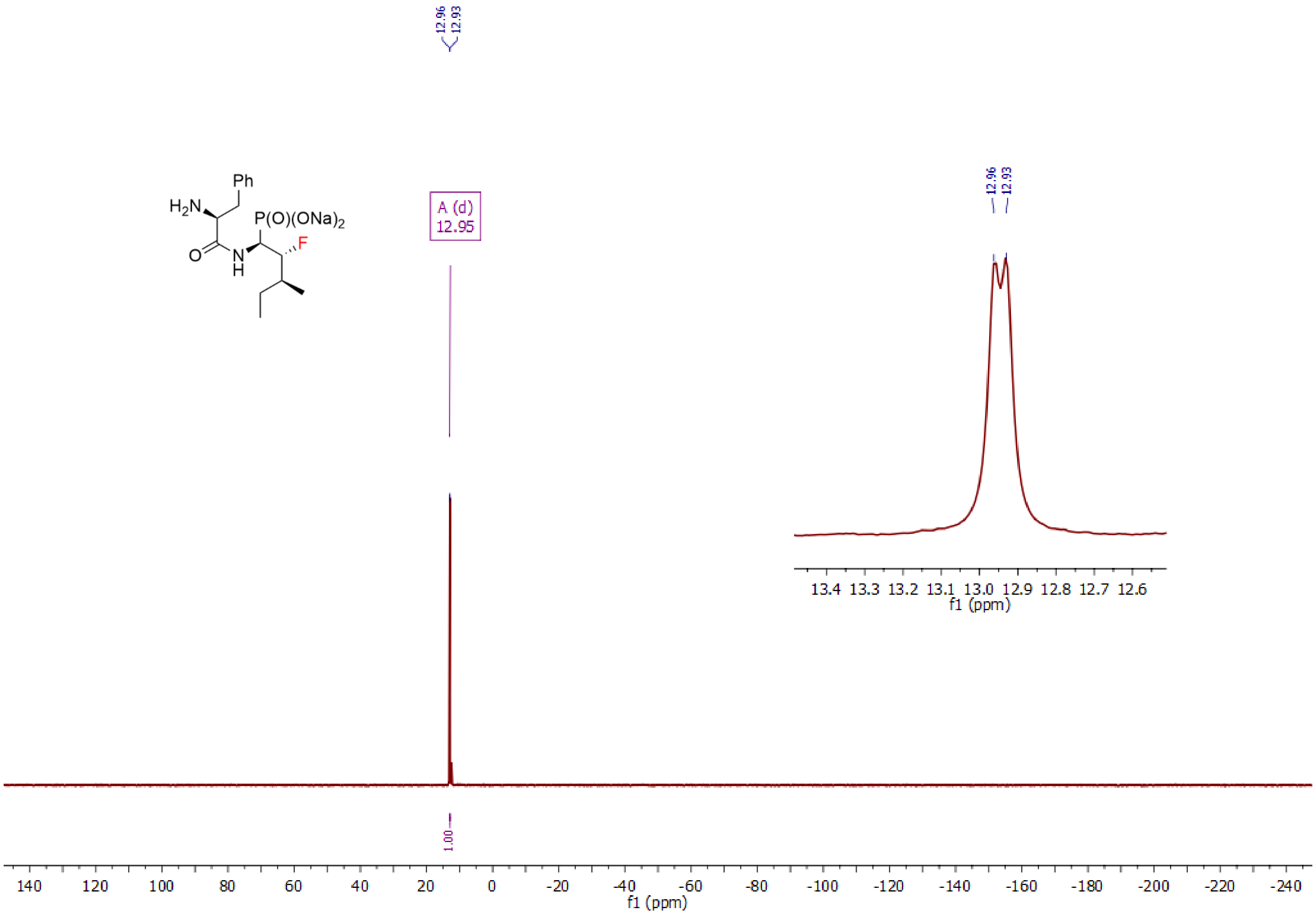

$^{31}P\{/^{1}H\}$ NMR (162 MHz D_2O) of **11d**.

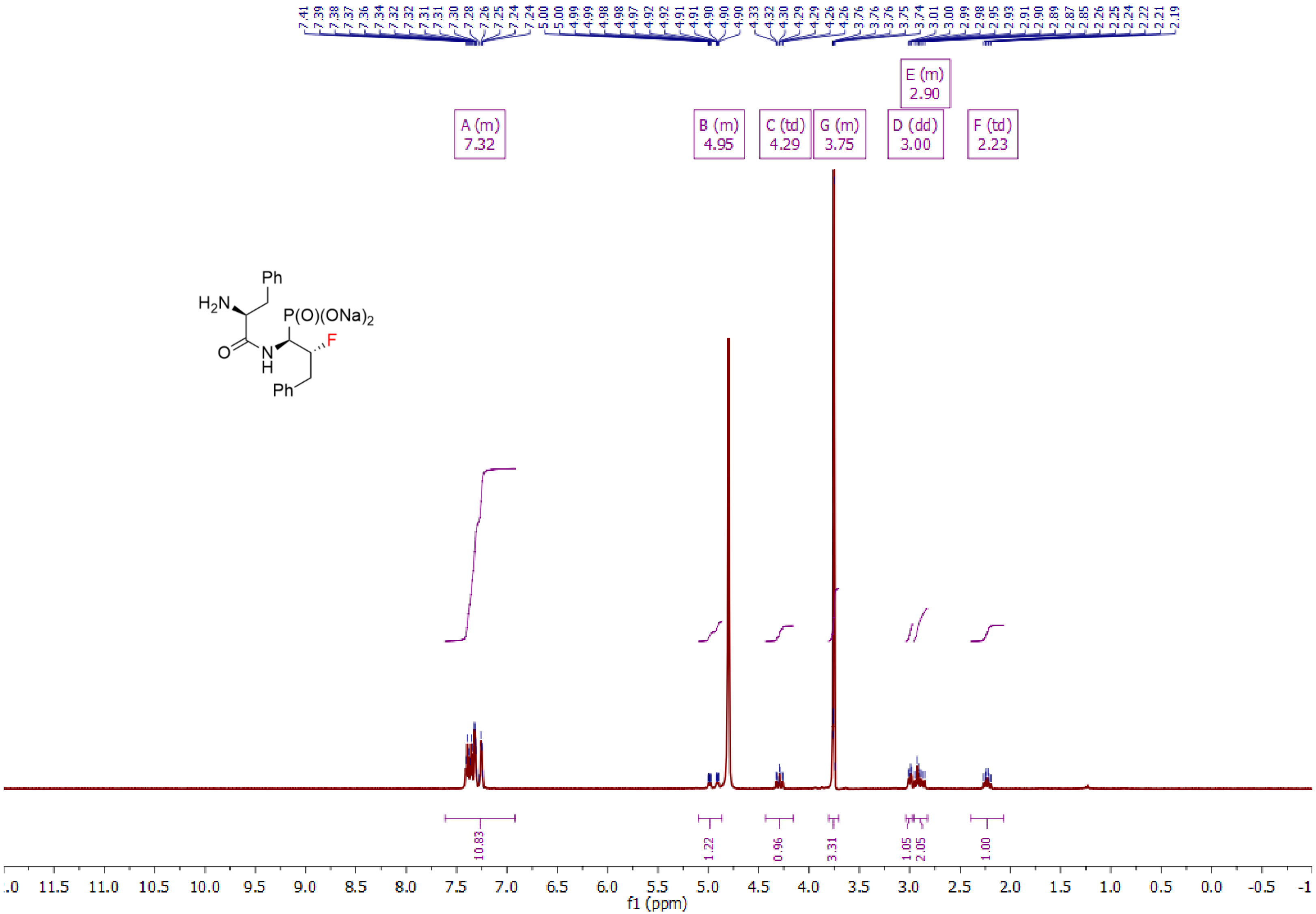

^{1}H NMR (600 MHz, D_2O) of **11e**.

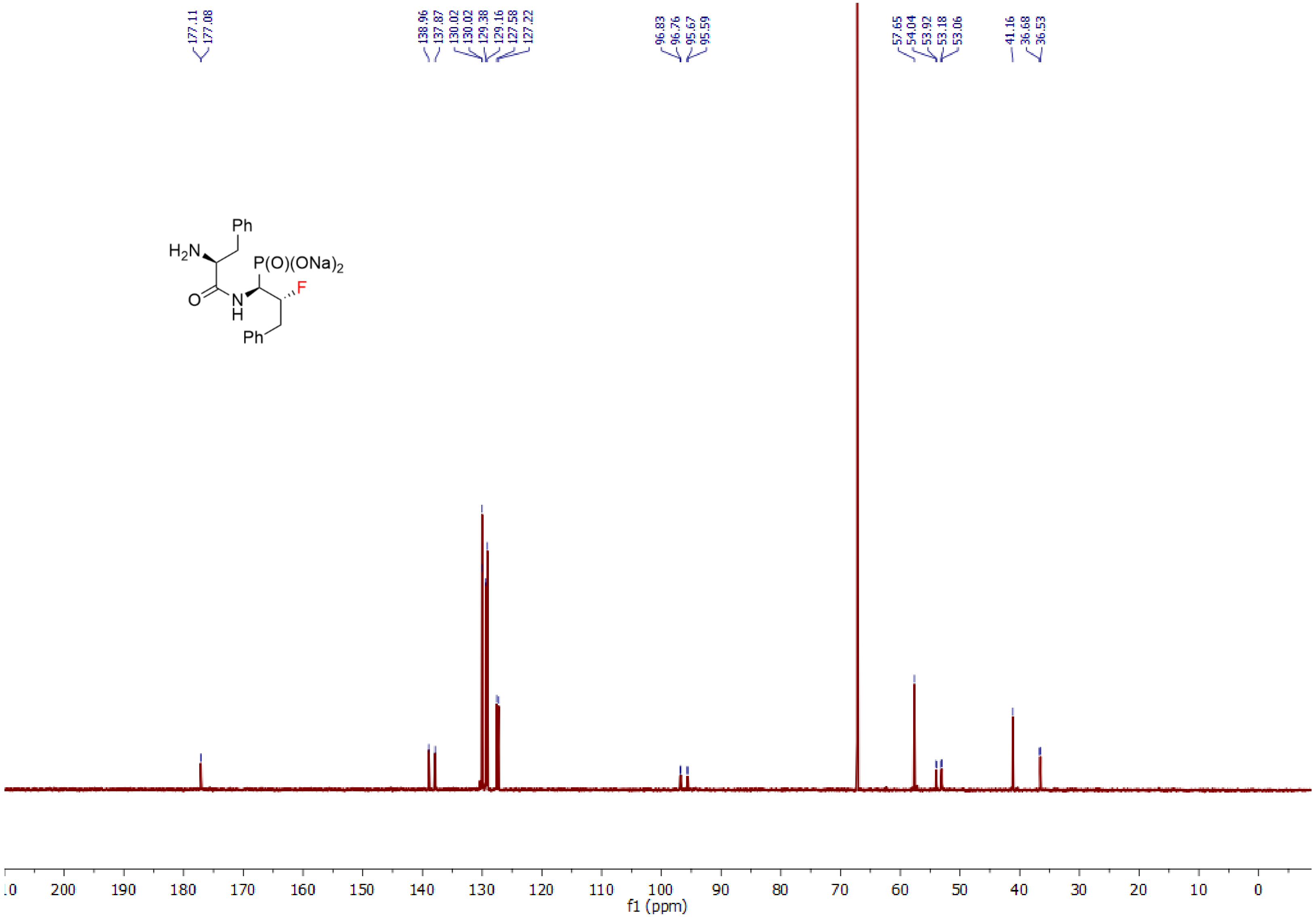

^{13}C NMR (151 MHz, D_2O) of **11e**.

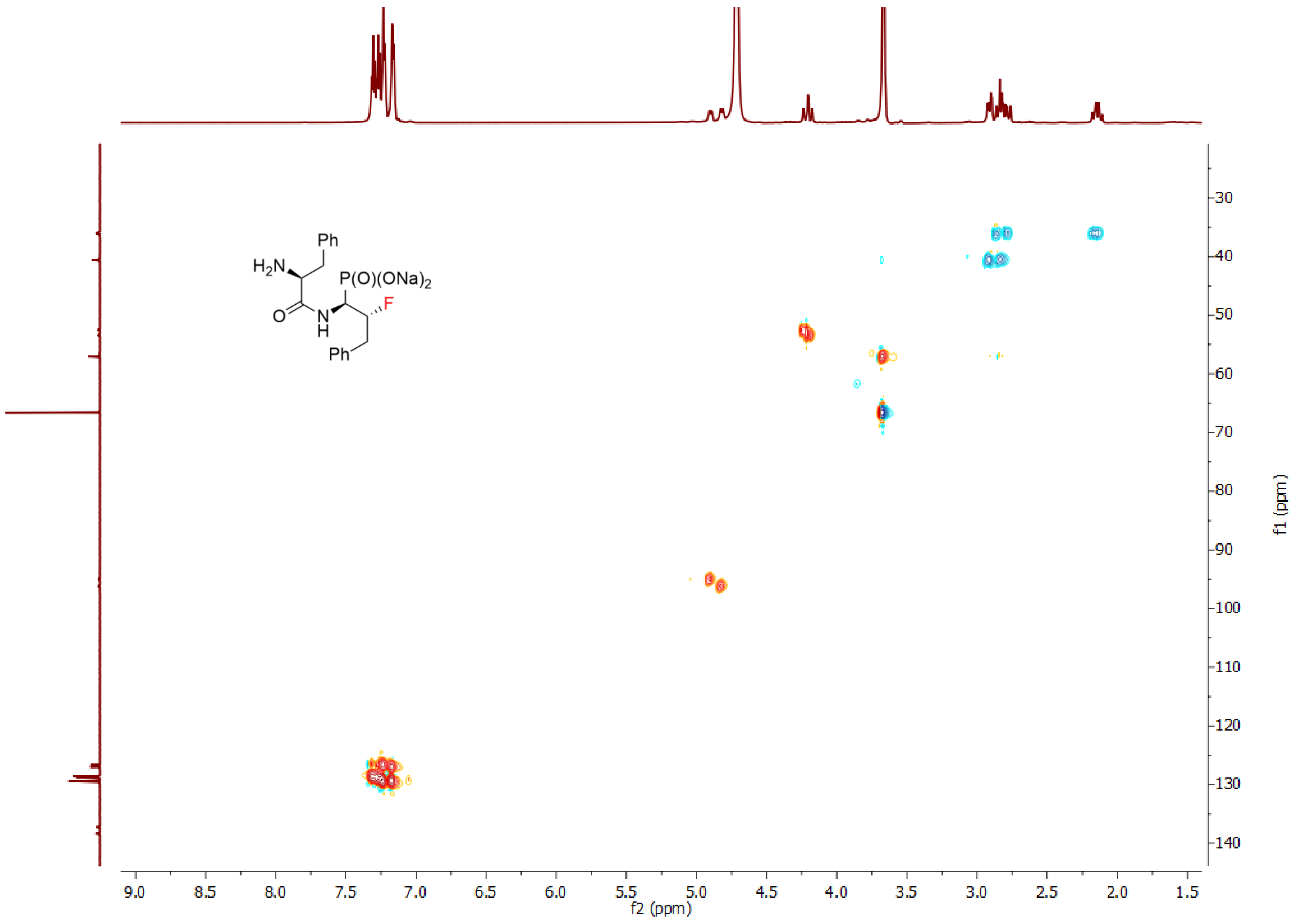

^{1}H-^{13}C HSQC (600 MHz, D_2O) of **11e**.

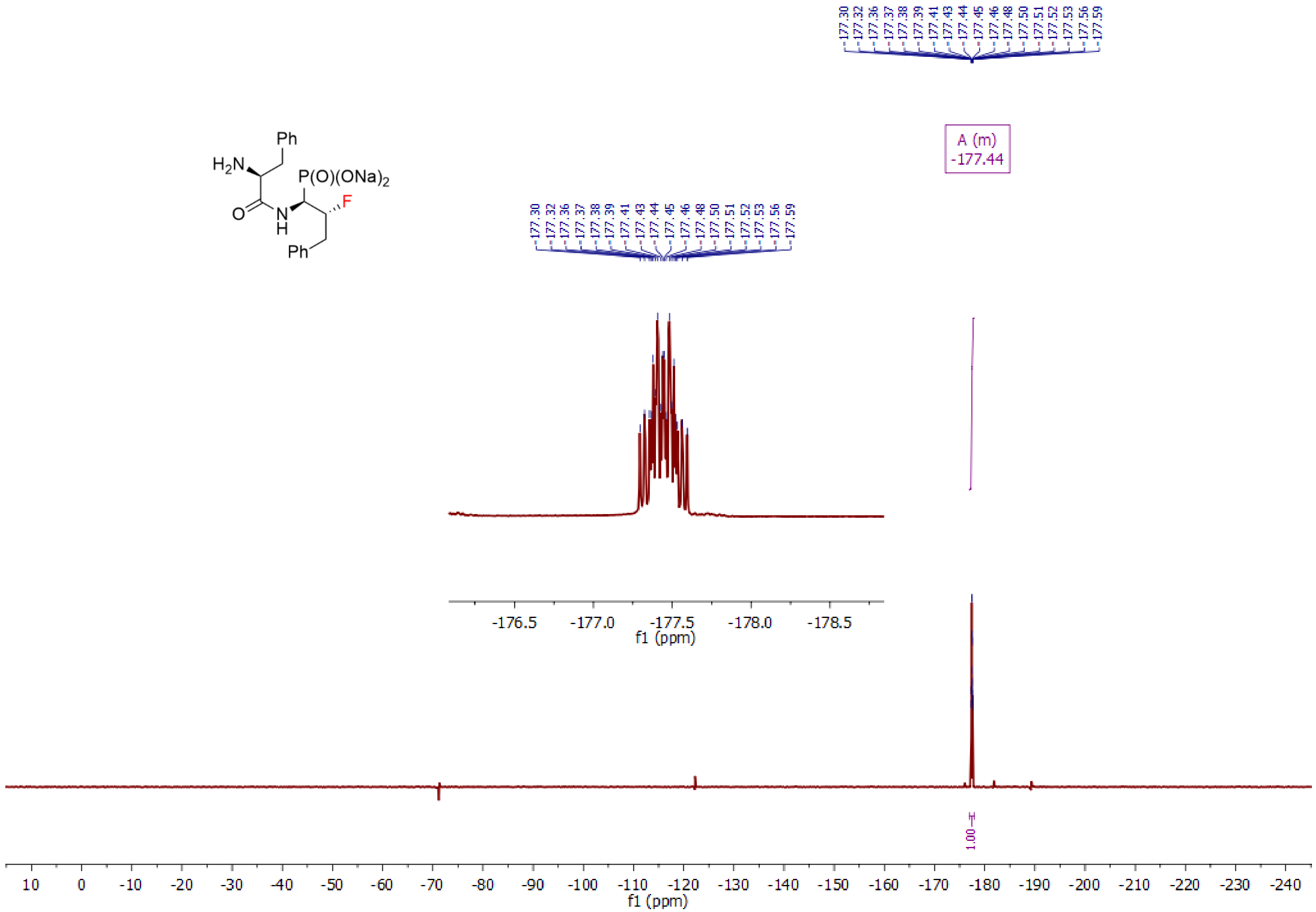

^{19}F NMR (565 MHz, D_2O) of **11e**.

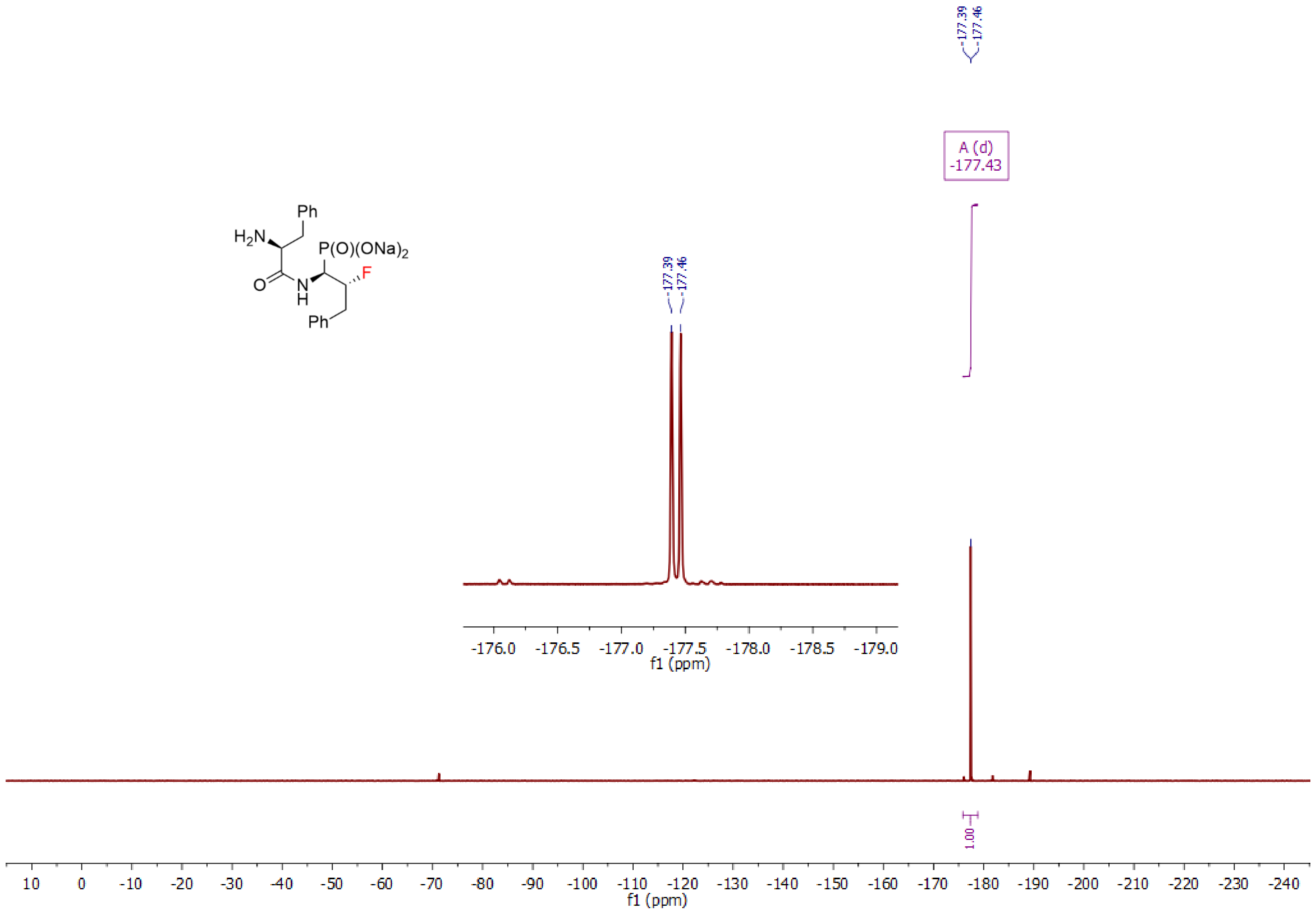

$^{19}F\{/^{1}H\}$ NMR (565 MHz, D_2O) of **11e**.

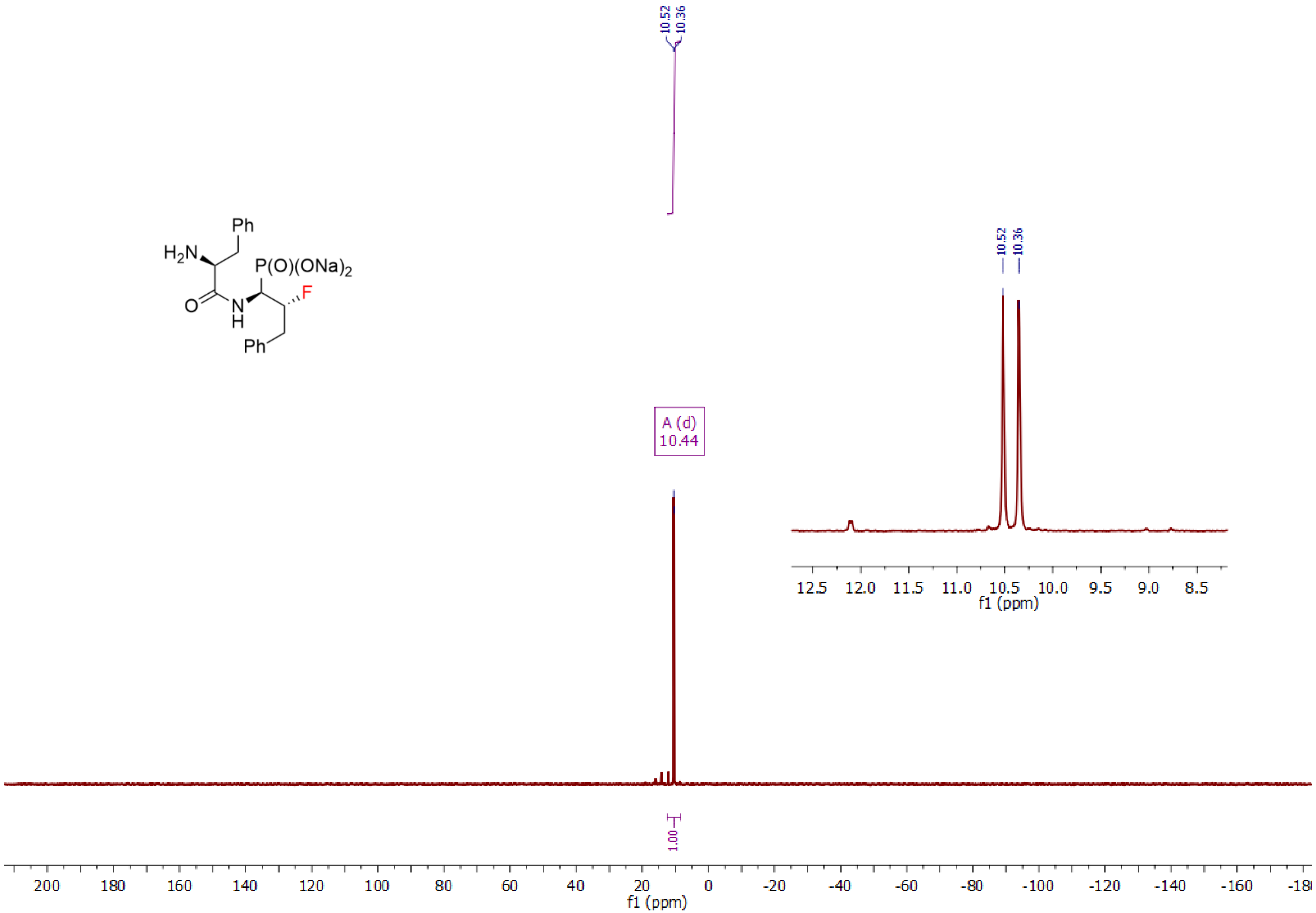

$^{31}P\{/^{1}H\}$ NMR (243 MHz, D_2O) of **11**.
